# Supplementary figures and images for: A systematic bi-genomic split-GFP assay illuminates the mitochondrial matrix proteome and protein targeting routes
Source: eLife. 2025 Dec 16;13:RP98889. doi: 10.7554/eLife.98889 (PMC12707816; doi:10.7554/eLife.98889)

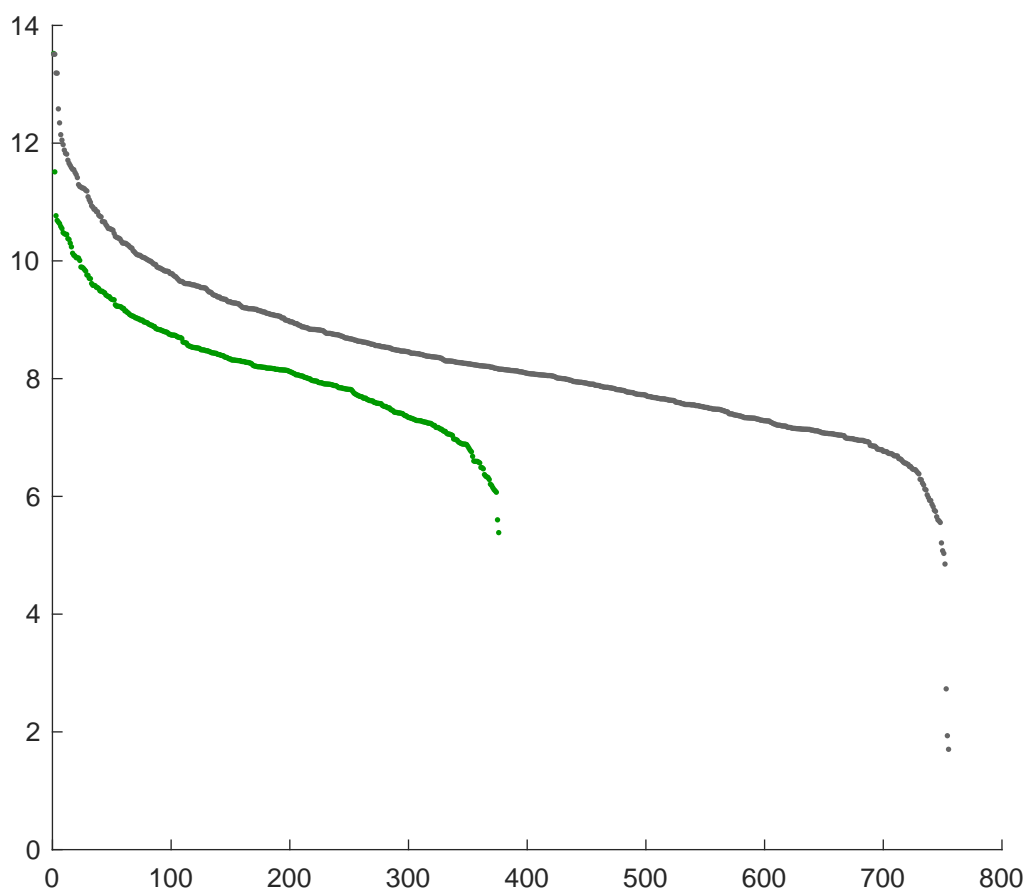

Supplement: Figure 2—source data 1. [file elife-98889-fig2-data1.zip › SourceData-Fig3/F1_Mito_HITS.pdf]

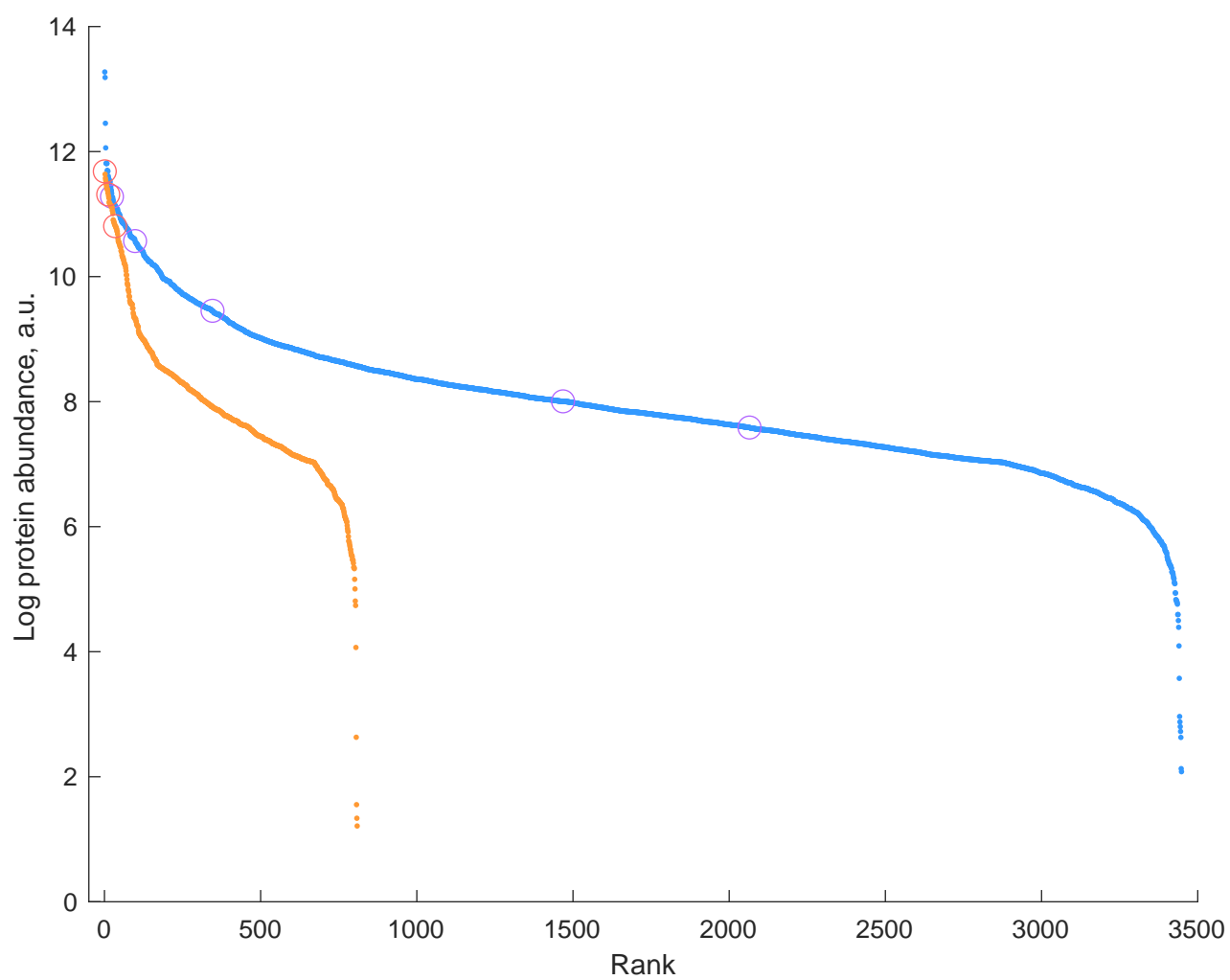

Supplement: Figure 2—source data 1. [file elife-98889-fig2-data1.zip › SourceData-Fig3/F1_nonMito_hits.pdf]

# ARC1

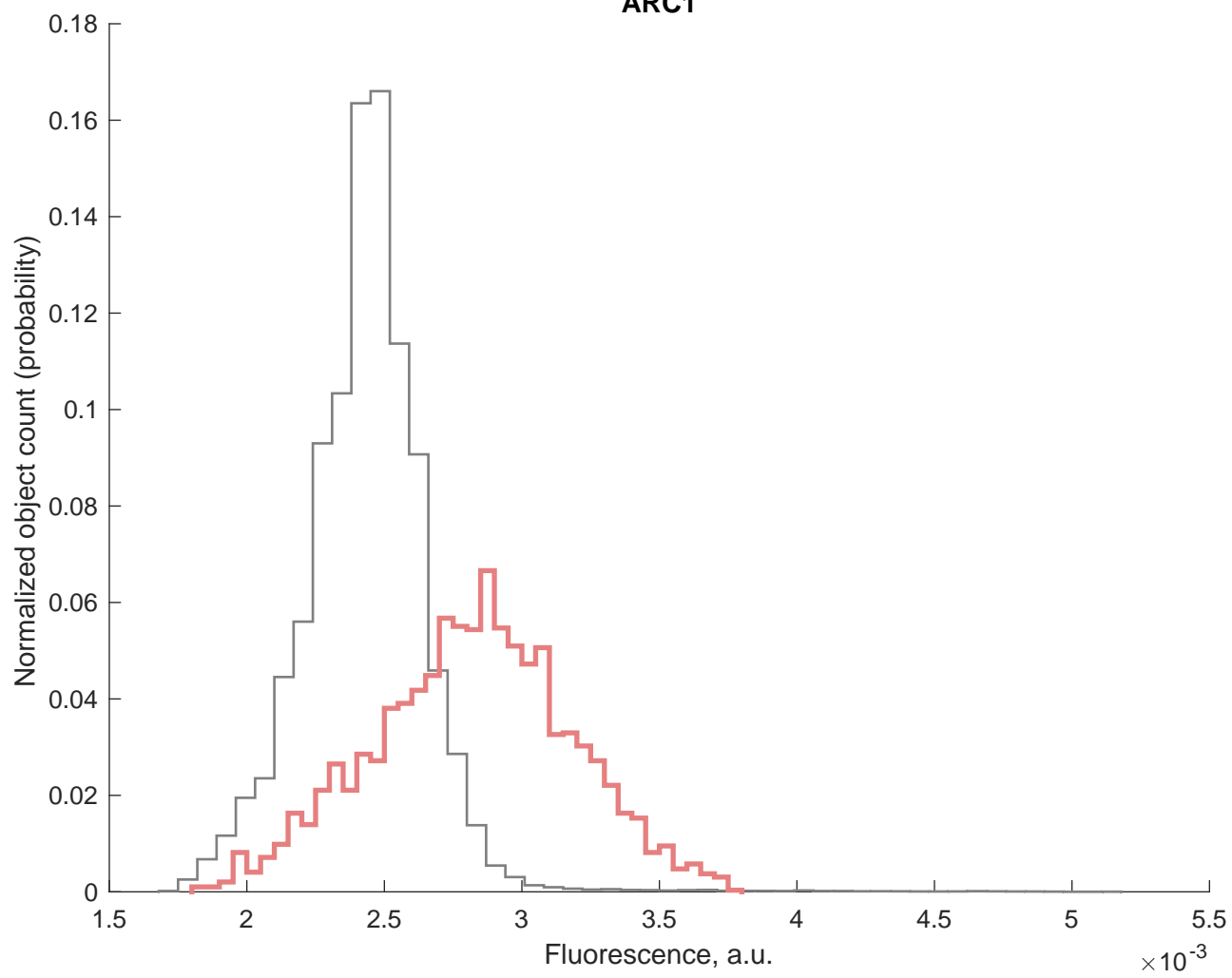

Supplement: Figure 2—source data 2. — The complete image dataset used for quantification is available on the BioImage Archive accession number S-BIAD2409. [file elife-98889-fig2-data2.zip › SourceData-Fig2B_splitGFP/p005_w111_ARC1.pdf]

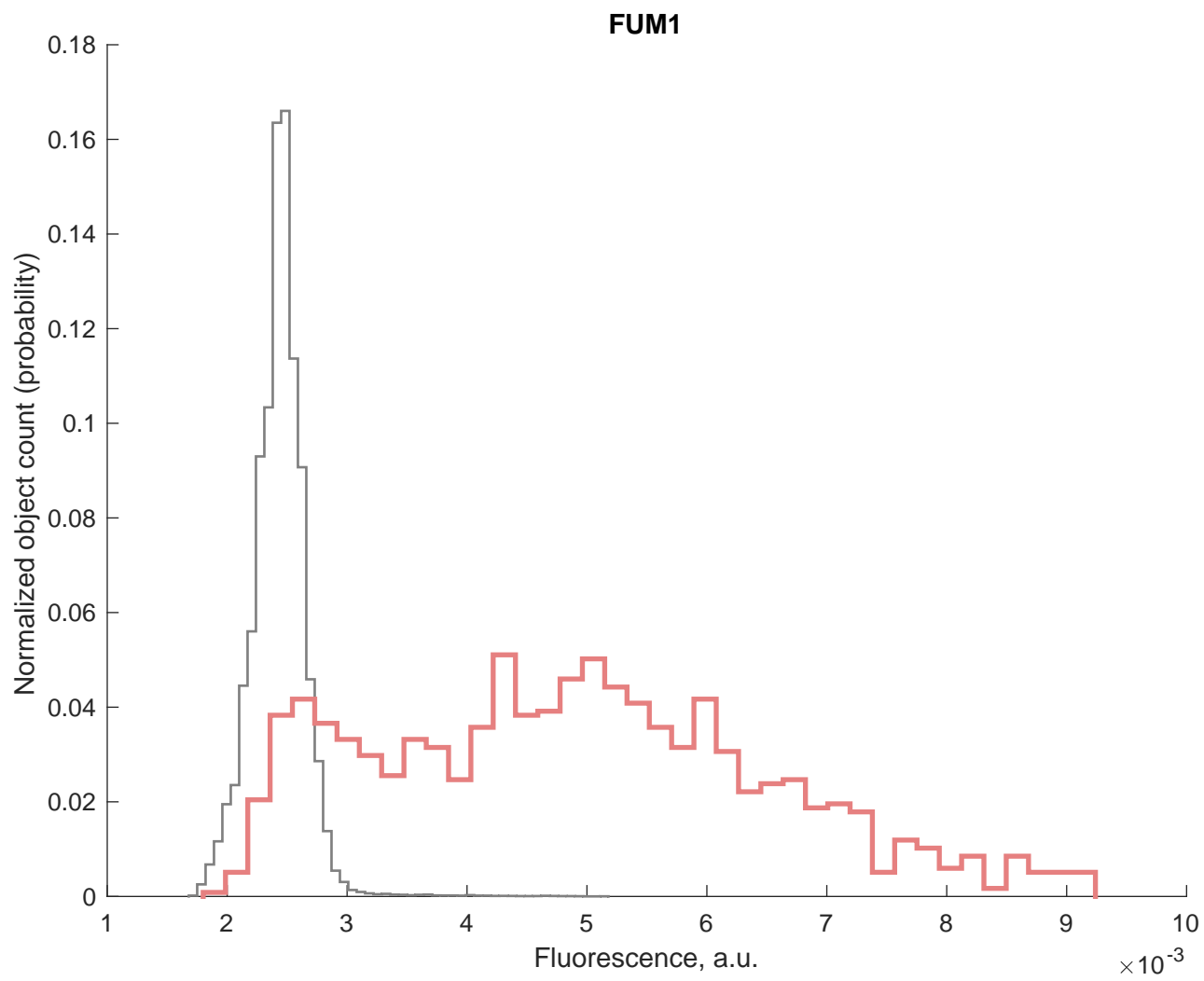

Supplement: Figure 2—source data 2. — The complete image dataset used for quantification is available on the BioImage Archive accession number S-BIAD2409. [file elife-98889-fig2-data2.zip › SourceData-Fig2B_splitGFP/p005_w31_FUM1.pdf]

**ILV6**

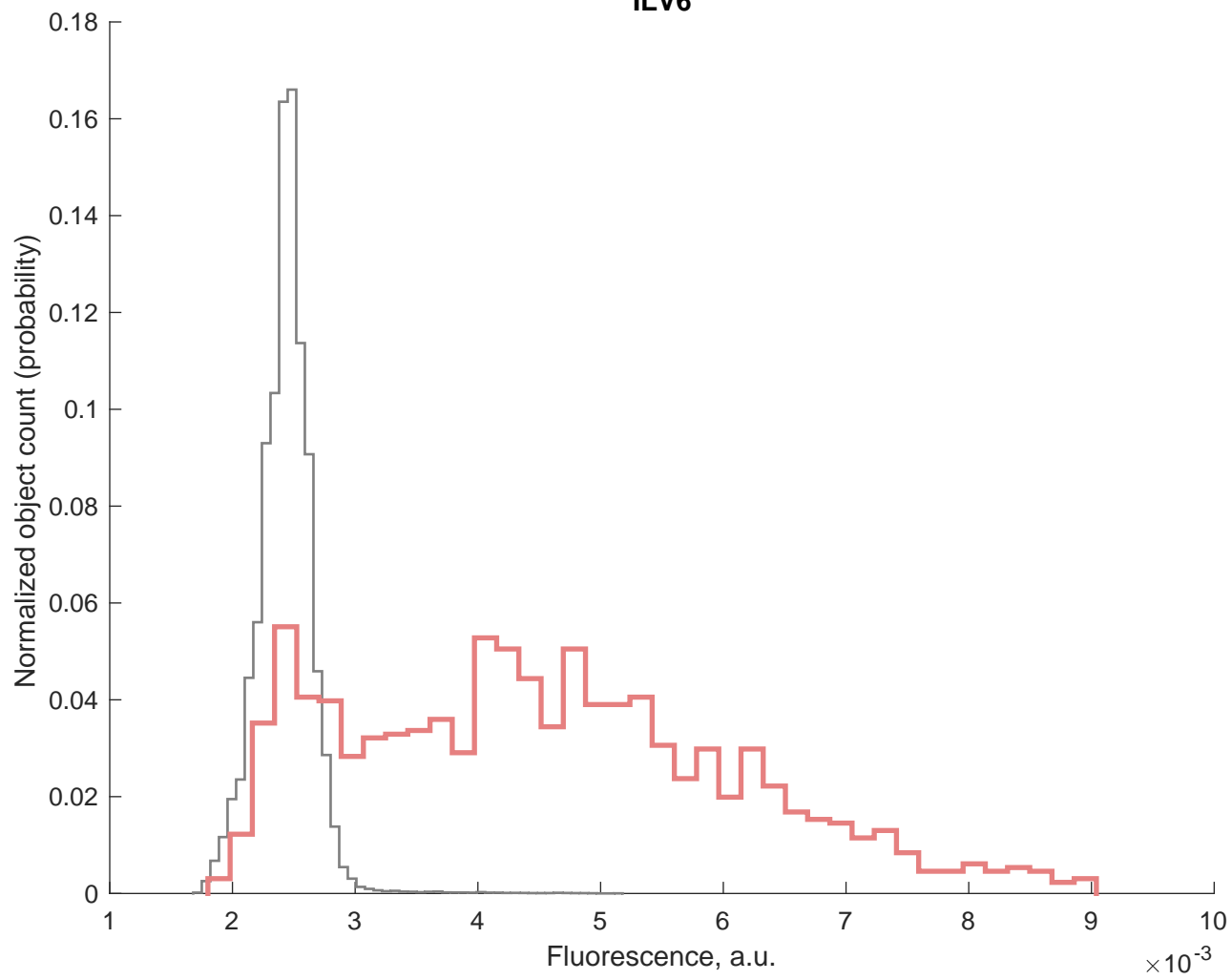

Supplement: Figure 2—source data 2. — The complete image dataset used for quantification is available on the BioImage Archive accession number S-BIAD2409. [file elife-98889-fig2-data2.zip › SourceData-Fig2B_splitGFP/p005_w219_ILV6.pdf]

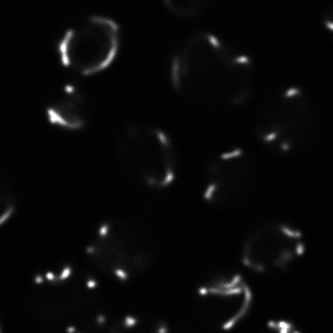

Supplement: Figure 2—source data 2. — The complete image dataset used for quantification is available on the BioImage Archive accession number S-BIAD2409. [file elife-98889-fig2-data2.zip › SourceData-Fig2B_splitGFP/Control_no_GFP11/GFP11/Control_mCH.tif]

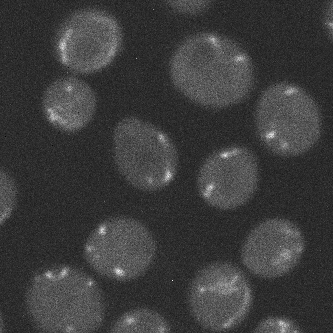

Supplement: Figure 2—source data 2. — The complete image dataset used for quantification is available on the BioImage Archive accession number S-BIAD2409. [file elife-98889-fig2-data2.zip › SourceData-Fig2B_splitGFP/Control_no_GFP11/GFP11/Control_GFP11.tif]

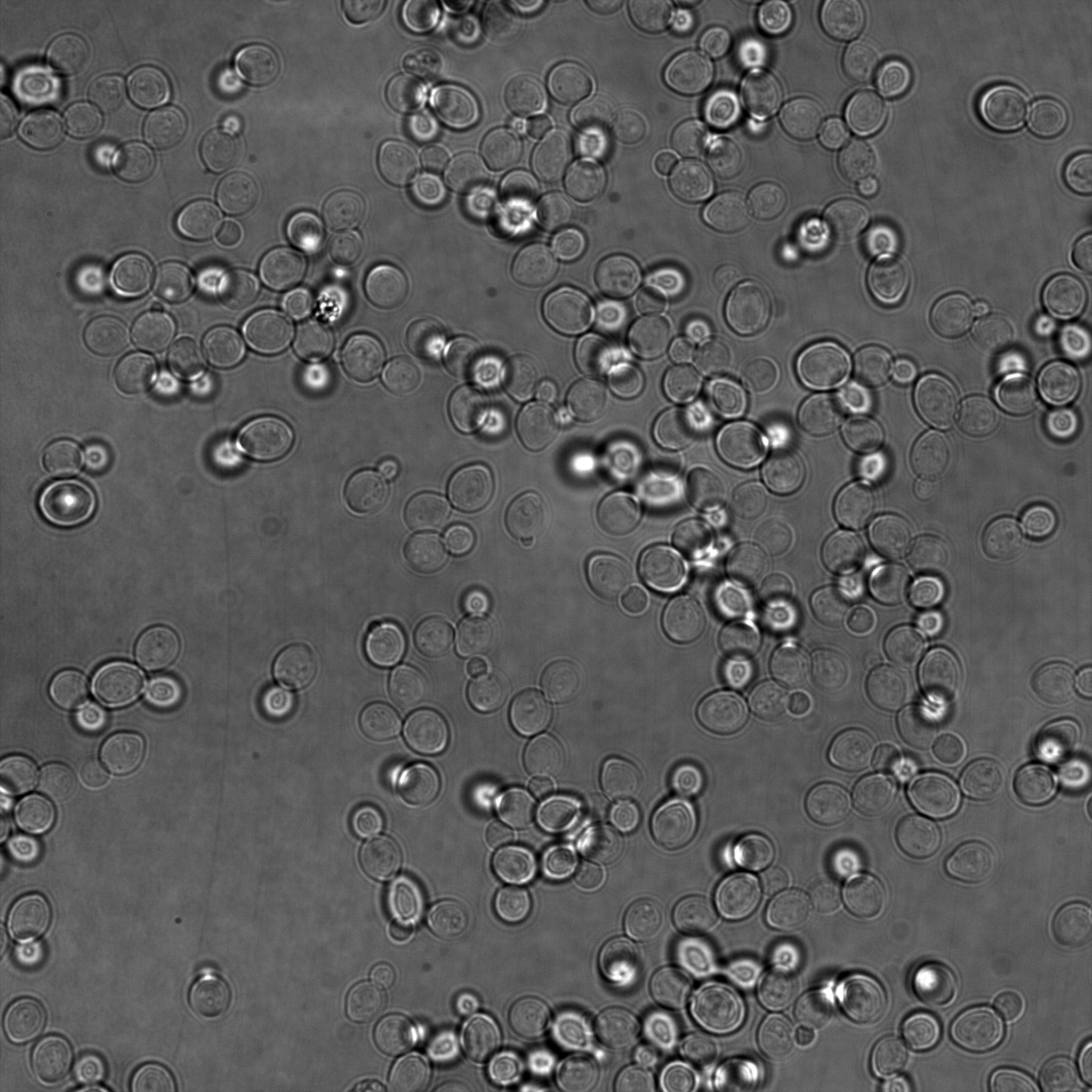

Supplement: Figure 2—source data 2. — The complete image dataset used for quantification is available on the BioImage Archive accession number S-BIAD2409. [file elife-98889-fig2-data2.zip › SourceData-Fig2B_splitGFP/Control_no_GFP11/GFP11/B6--W00030--P00001--Z00000--T00000--TransCon.tif]

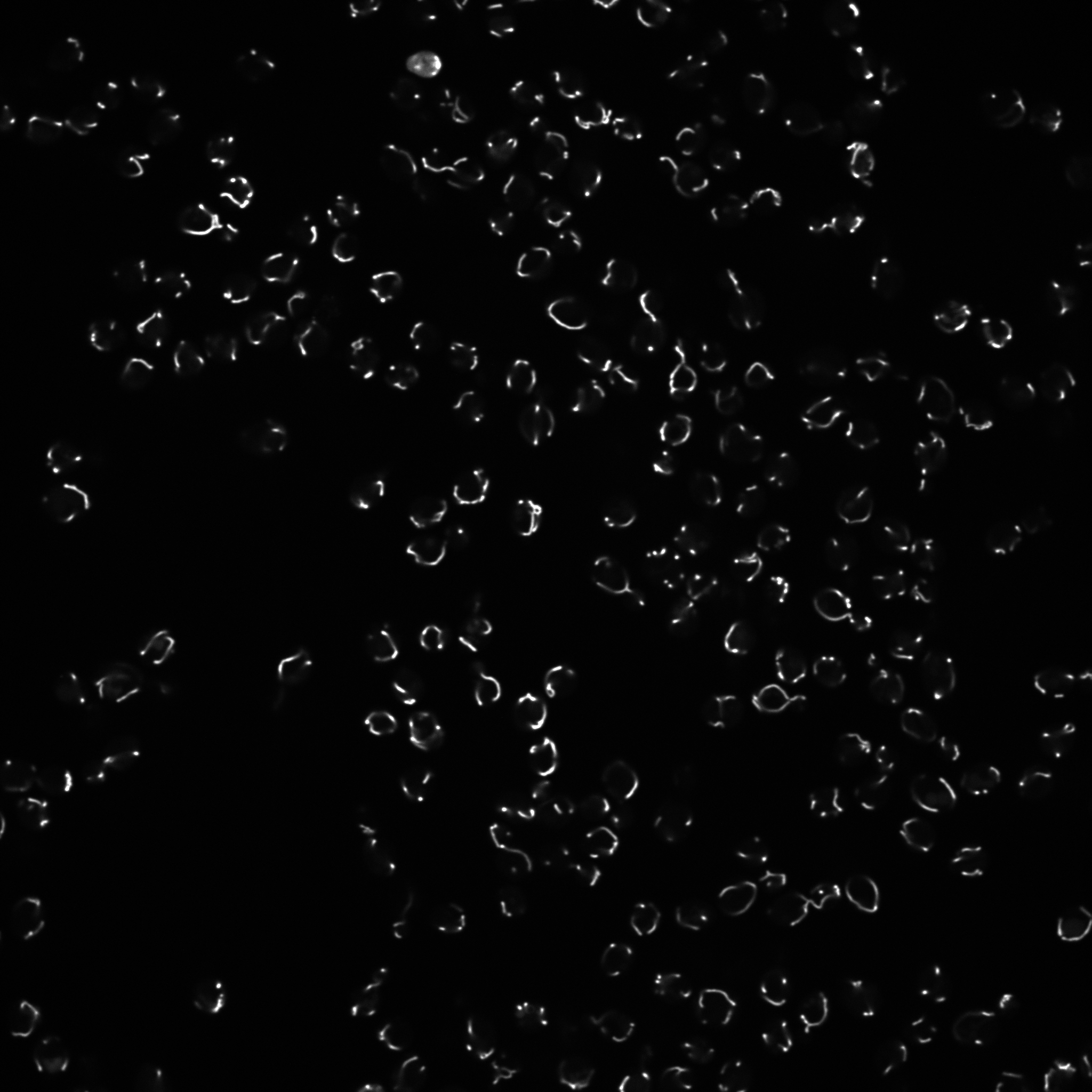

Supplement: Figure 2—source data 2. — The complete image dataset used for quantification is available on the BioImage Archive accession number S-BIAD2409. [file elife-98889-fig2-data2.zip › SourceData-Fig2B_splitGFP/Control_no_GFP11/GFP11/B6--W00030--P00001--Z00000--T00000--561nm.tif]

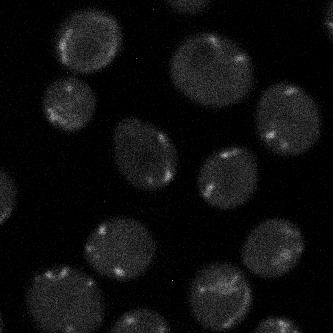

Supplement: Figure 2—source data 2. — The complete image dataset used for quantification is available on the BioImage Archive accession number S-BIAD2409. [file elife-98889-fig2-data2.zip › SourceData-Fig2B_splitGFP/Control_no_GFP11/GFP11/Control_GFP11.png]

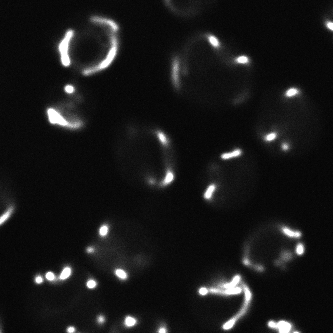

Supplement: Figure 2—source data 2. — The complete image dataset used for quantification is available on the BioImage Archive accession number S-BIAD2409. [file elife-98889-fig2-data2.zip › SourceData-Fig2B_splitGFP/Control_no_GFP11/GFP11/Control_mCH.png]

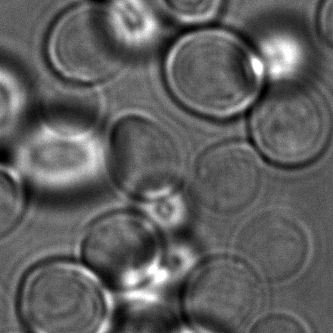

Supplement: Figure 2—source data 2. — The complete image dataset used for quantification is available on the BioImage Archive accession number S-BIAD2409. [file elife-98889-fig2-data2.zip › SourceData-Fig2B_splitGFP/Control_no_GFP11/GFP11/Control_GFP11_Tr.png]

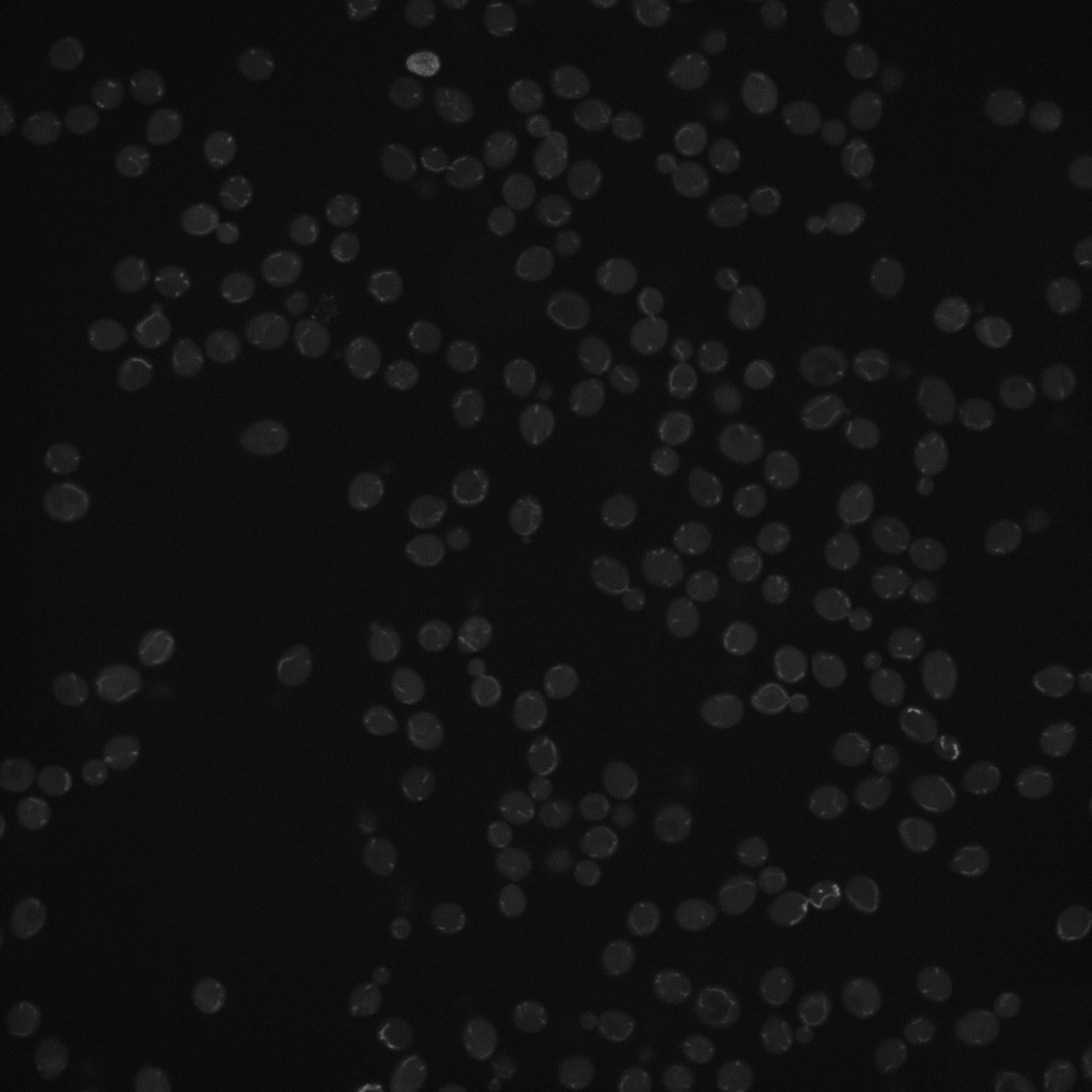

Supplement: Figure 2—source data 2. — The complete image dataset used for quantification is available on the BioImage Archive accession number S-BIAD2409. [file elife-98889-fig2-data2.zip › SourceData-Fig2B_splitGFP/Control_no_GFP11/GFP11/B6--W00030--P00001--Z00000--T00000--488nm.tif]

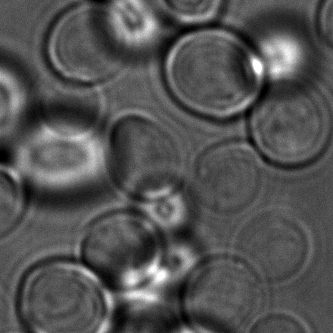

Supplement: Figure 2—source data 2. — The complete image dataset used for quantification is available on the BioImage Archive accession number S-BIAD2409. [file elife-98889-fig2-data2.zip › SourceData-Fig2B_splitGFP/Control_no_GFP11/GFP11/Control_GFP11_Tr.tif]

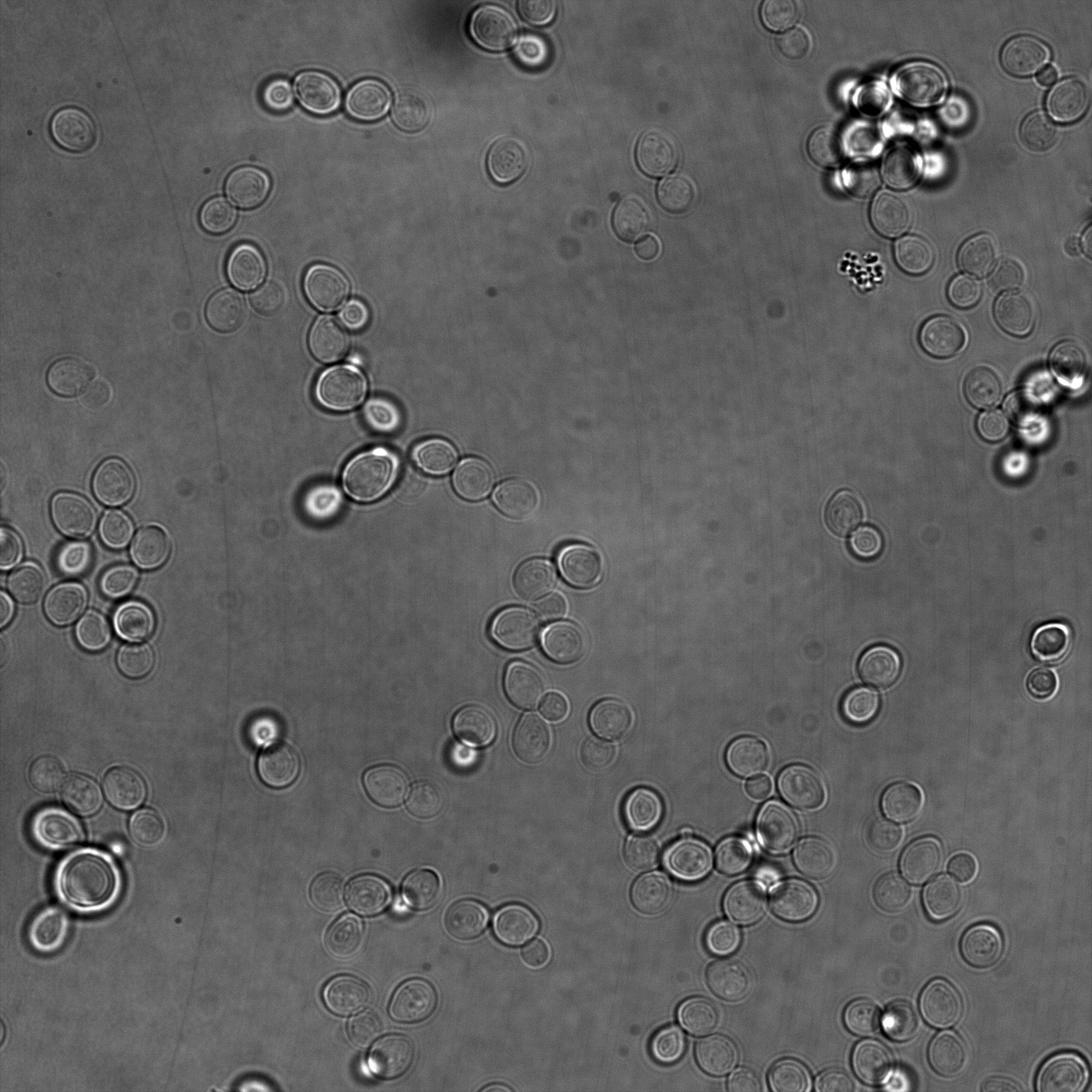

Supplement: Figure 2—source data 2. — The complete image dataset used for quantification is available on the BioImage Archive accession number S-BIAD2409. [file elife-98889-fig2-data2.zip › SourceData-Fig2B_splitGFP/Genuine_mito_proteins/ILV6/GFP11/J3--W00219--P00002--Z00000--T00000--TransCon.tif]

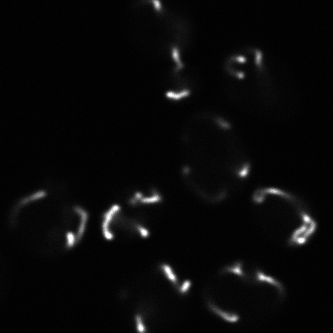

Supplement: Figure 2—source data 2. — The complete image dataset used for quantification is available on the BioImage Archive accession number S-BIAD2409. [file elife-98889-fig2-data2.zip › SourceData-Fig2B_splitGFP/Genuine_mito_proteins/ILV6/GFP11/ILV6_mCH.tif]

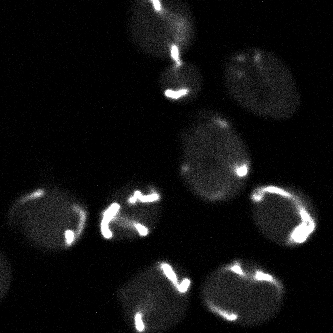

Supplement: Figure 2—source data 2. — The complete image dataset used for quantification is available on the BioImage Archive accession number S-BIAD2409. [file elife-98889-fig2-data2.zip › SourceData-Fig2B_splitGFP/Genuine_mito_proteins/ILV6/GFP11/ILV6_GFP11.png]

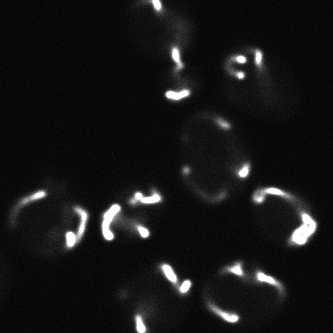

Supplement: Figure 2—source data 2. — The complete image dataset used for quantification is available on the BioImage Archive accession number S-BIAD2409. [file elife-98889-fig2-data2.zip › SourceData-Fig2B_splitGFP/Genuine_mito_proteins/ILV6/GFP11/ILV6_mCH.png]

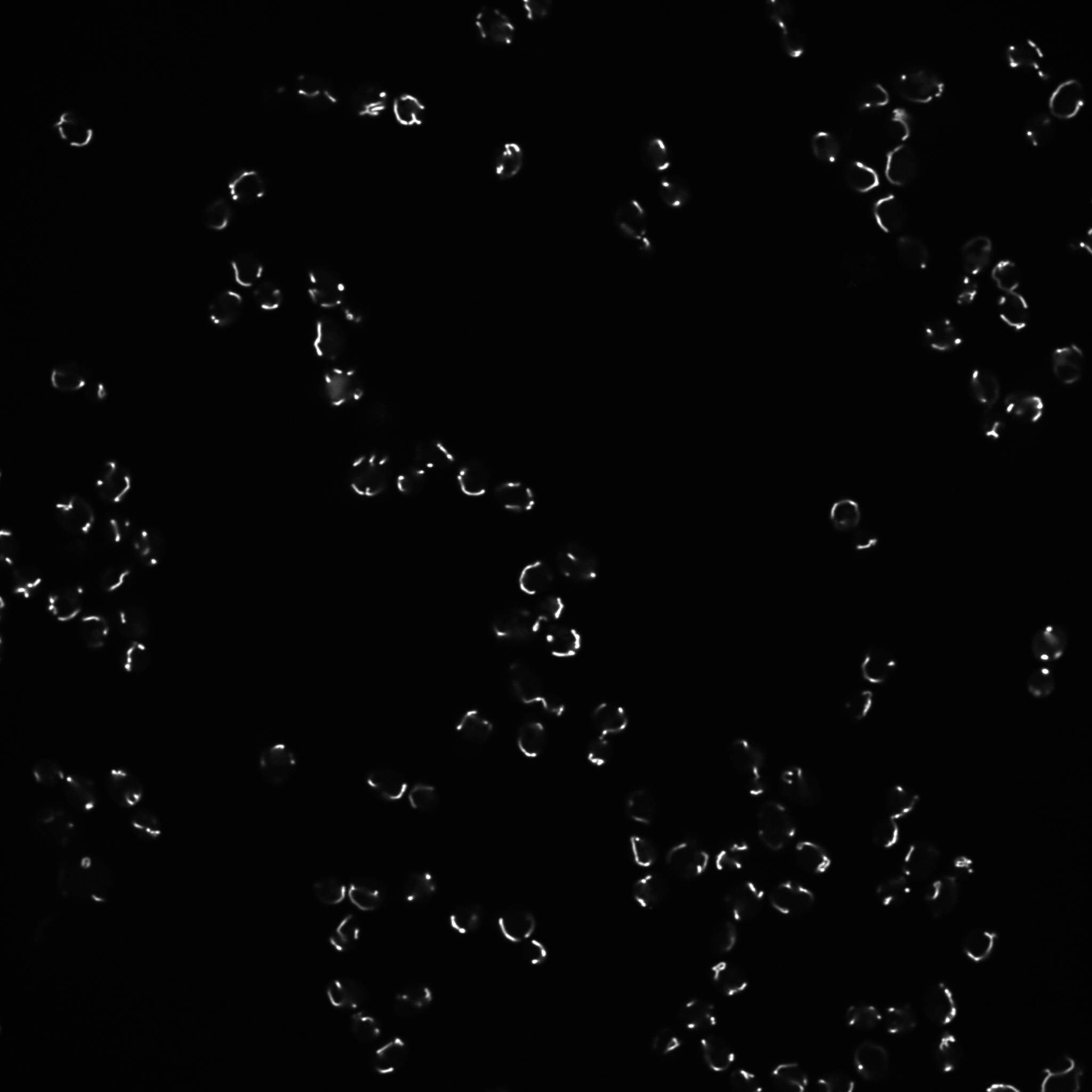

Supplement: Figure 2—source data 2. — The complete image dataset used for quantification is available on the BioImage Archive accession number S-BIAD2409. [file elife-98889-fig2-data2.zip › SourceData-Fig2B_splitGFP/Genuine_mito_proteins/ILV6/GFP11/J3--W00219--P00002--Z00000--T00000--561nm.tif]

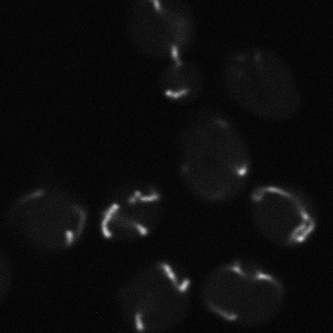

Supplement: Figure 2—source data 2. — The complete image dataset used for quantification is available on the BioImage Archive accession number S-BIAD2409. [file elife-98889-fig2-data2.zip › SourceData-Fig2B_splitGFP/Genuine_mito_proteins/ILV6/GFP11/ILV6_GFP11.tif]

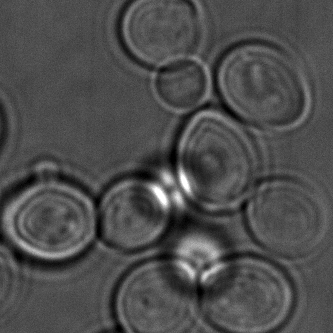

Supplement: Figure 2—source data 2. — The complete image dataset used for quantification is available on the BioImage Archive accession number S-BIAD2409. [file elife-98889-fig2-data2.zip › SourceData-Fig2B_splitGFP/Genuine_mito_proteins/ILV6/GFP11/ILV6_GFP11_Tr.tif]

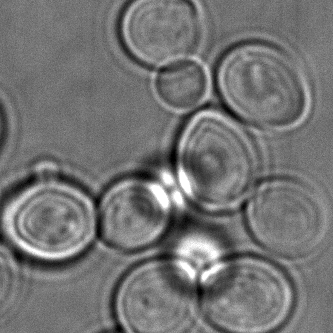

Supplement: Figure 2—source data 2. — The complete image dataset used for quantification is available on the BioImage Archive accession number S-BIAD2409. [file elife-98889-fig2-data2.zip › SourceData-Fig2B_splitGFP/Genuine_mito_proteins/ILV6/GFP11/ILV6_GFP11_Tr.png]

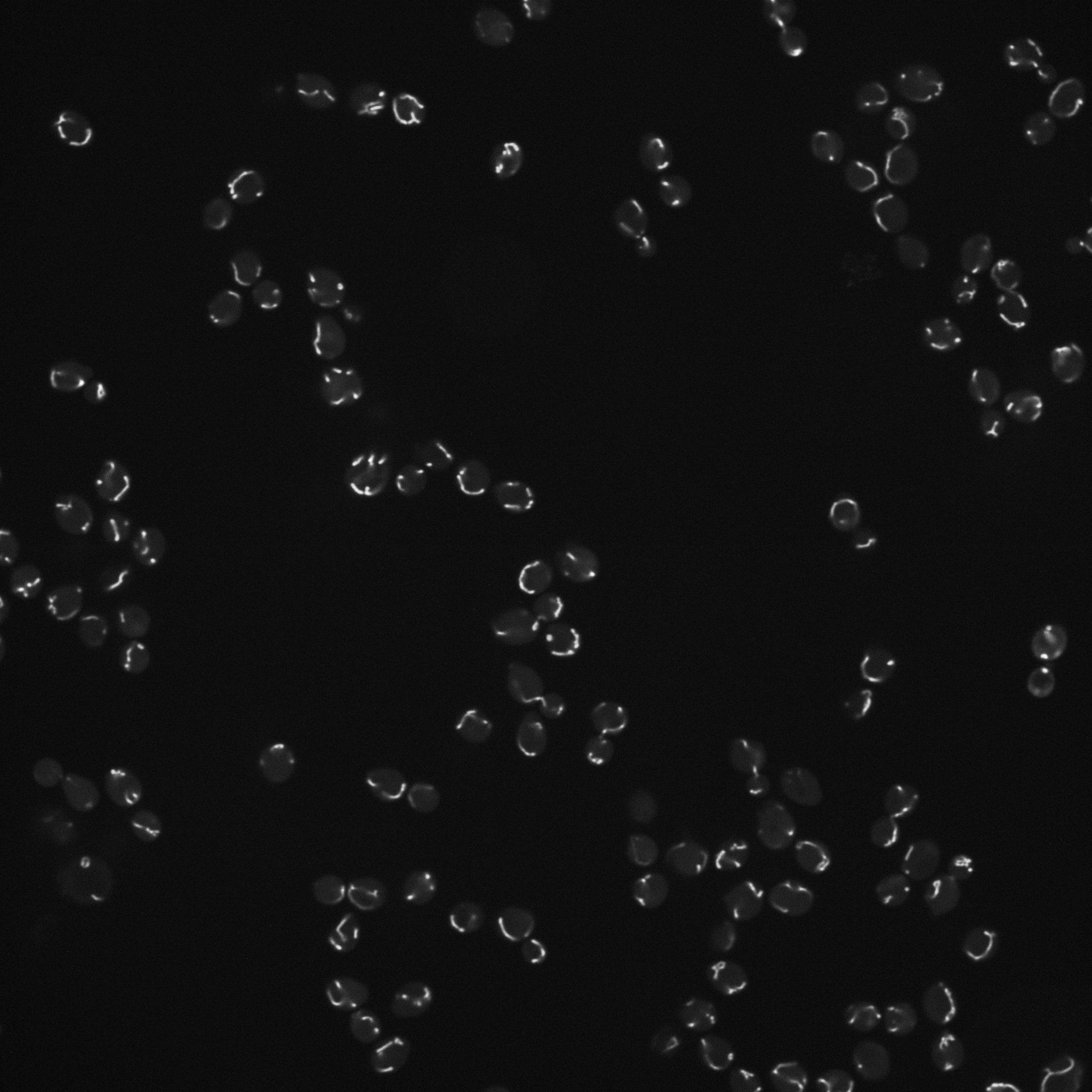

Supplement: Figure 2—source data 2. — The complete image dataset used for quantification is available on the BioImage Archive accession number S-BIAD2409. [file elife-98889-fig2-data2.zip › SourceData-Fig2B_splitGFP/Genuine_mito_proteins/ILV6/GFP11/J3--W00219--P00002--Z00000--T00000--488nm.tif]

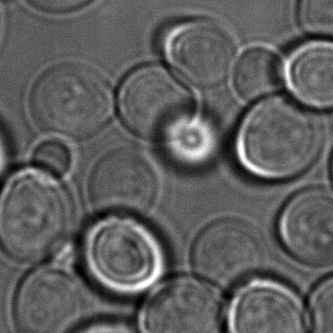

Supplement: Figure 2—source data 2. — The complete image dataset used for quantification is available on the BioImage Archive accession number S-BIAD2409. [file elife-98889-fig2-data2.zip › SourceData-Fig2B_splitGFP/Dual_no_mito/ARC1/GFP11/ARC1_GFP11_tr.tif]

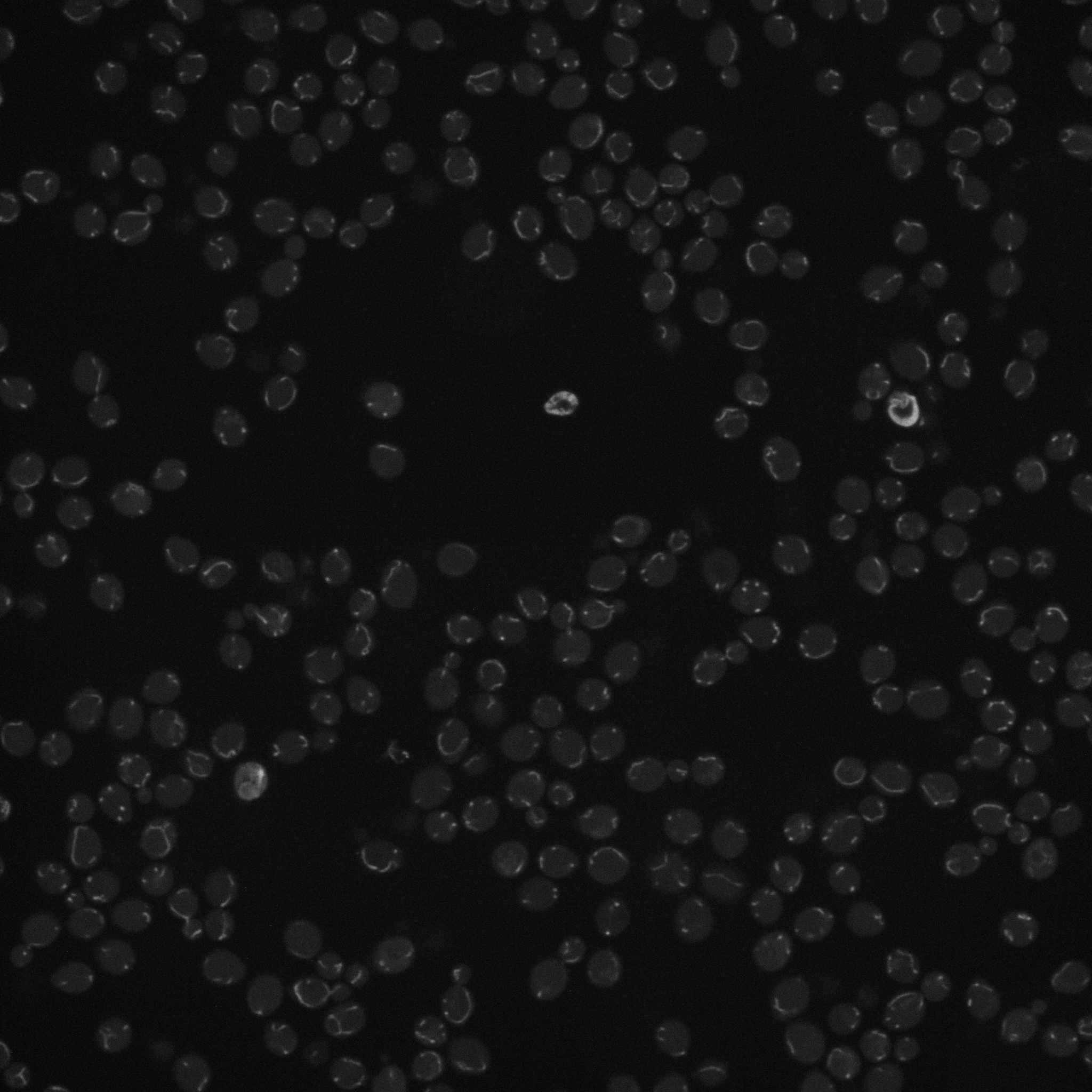

Supplement: Figure 2—source data 2. — The complete image dataset used for quantification is available on the BioImage Archive accession number S-BIAD2409. [file elife-98889-fig2-data2.zip › SourceData-Fig2B_splitGFP/Dual_no_mito/ARC1/GFP11/E15--W00111--P00001--Z00000--T00000--488nm.tif]

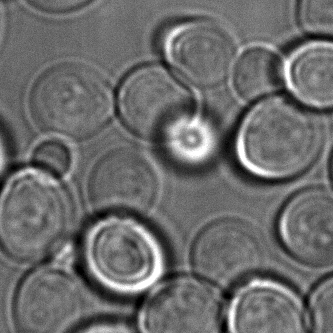

Supplement: Figure 2—source data 2. — The complete image dataset used for quantification is available on the BioImage Archive accession number S-BIAD2409. [file elife-98889-fig2-data2.zip › SourceData-Fig2B_splitGFP/Dual_no_mito/ARC1/GFP11/ARC1_GFP11_tr.png]

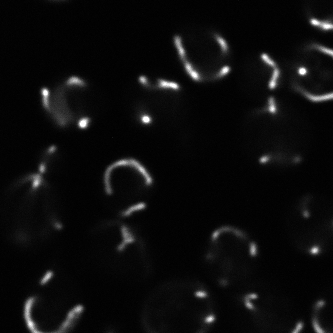

Supplement: Figure 2—source data 2. — The complete image dataset used for quantification is available on the BioImage Archive accession number S-BIAD2409. [file elife-98889-fig2-data2.zip › SourceData-Fig2B_splitGFP/Dual_no_mito/ARC1/GFP11/ARC1_mCH.tif]

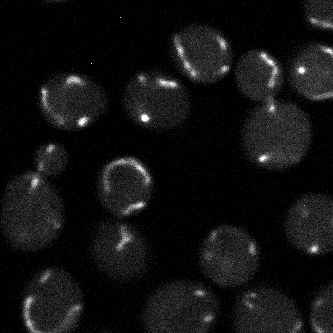

Supplement: Figure 2—source data 2. — The complete image dataset used for quantification is available on the BioImage Archive accession number S-BIAD2409. [file elife-98889-fig2-data2.zip › SourceData-Fig2B_splitGFP/Dual_no_mito/ARC1/GFP11/ARC1_GFP11.png]

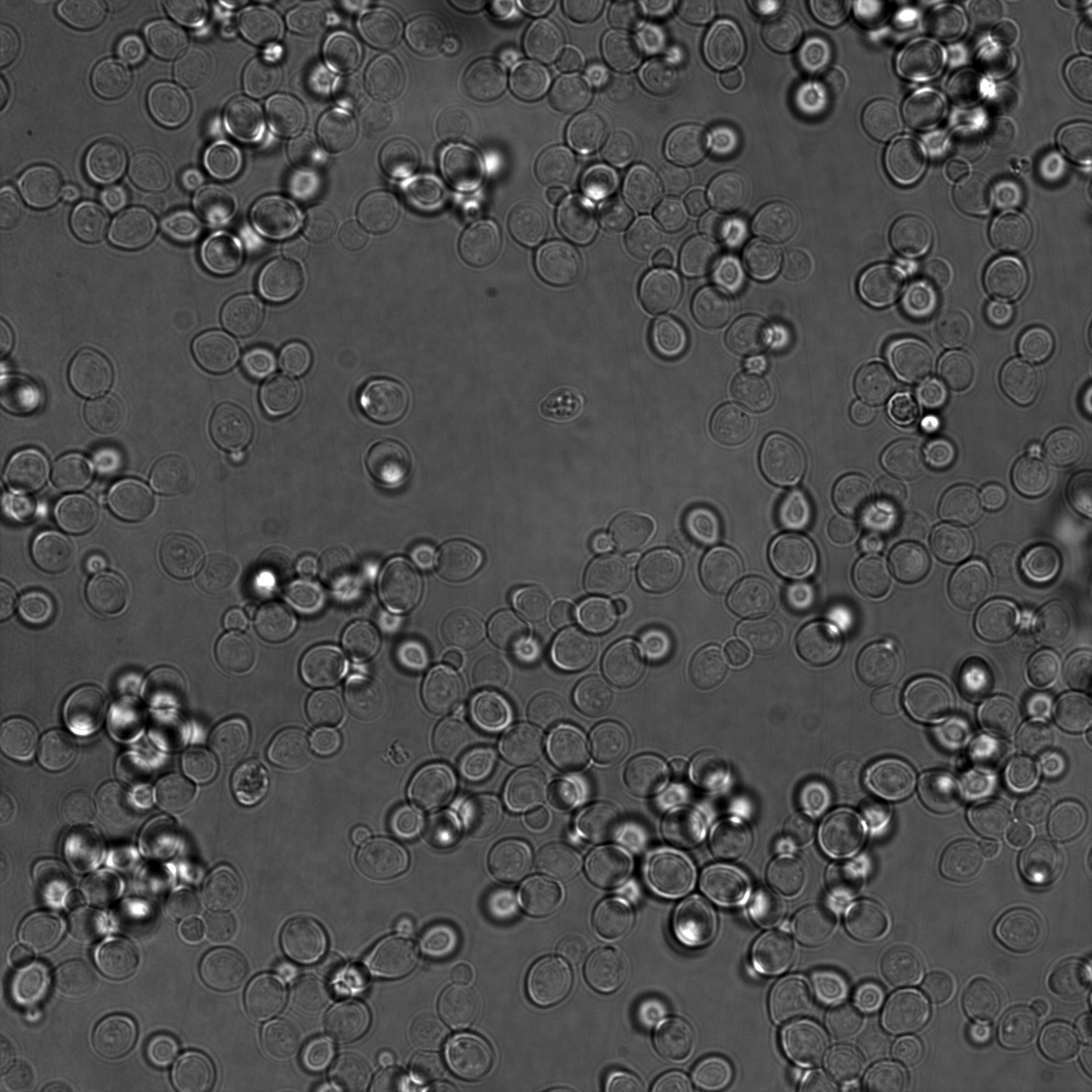

Supplement: Figure 2—source data 2. — The complete image dataset used for quantification is available on the BioImage Archive accession number S-BIAD2409. [file elife-98889-fig2-data2.zip › SourceData-Fig2B_splitGFP/Dual_no_mito/ARC1/GFP11/E15--W00111--P00001--Z00000--T00000--TransCon.tif]

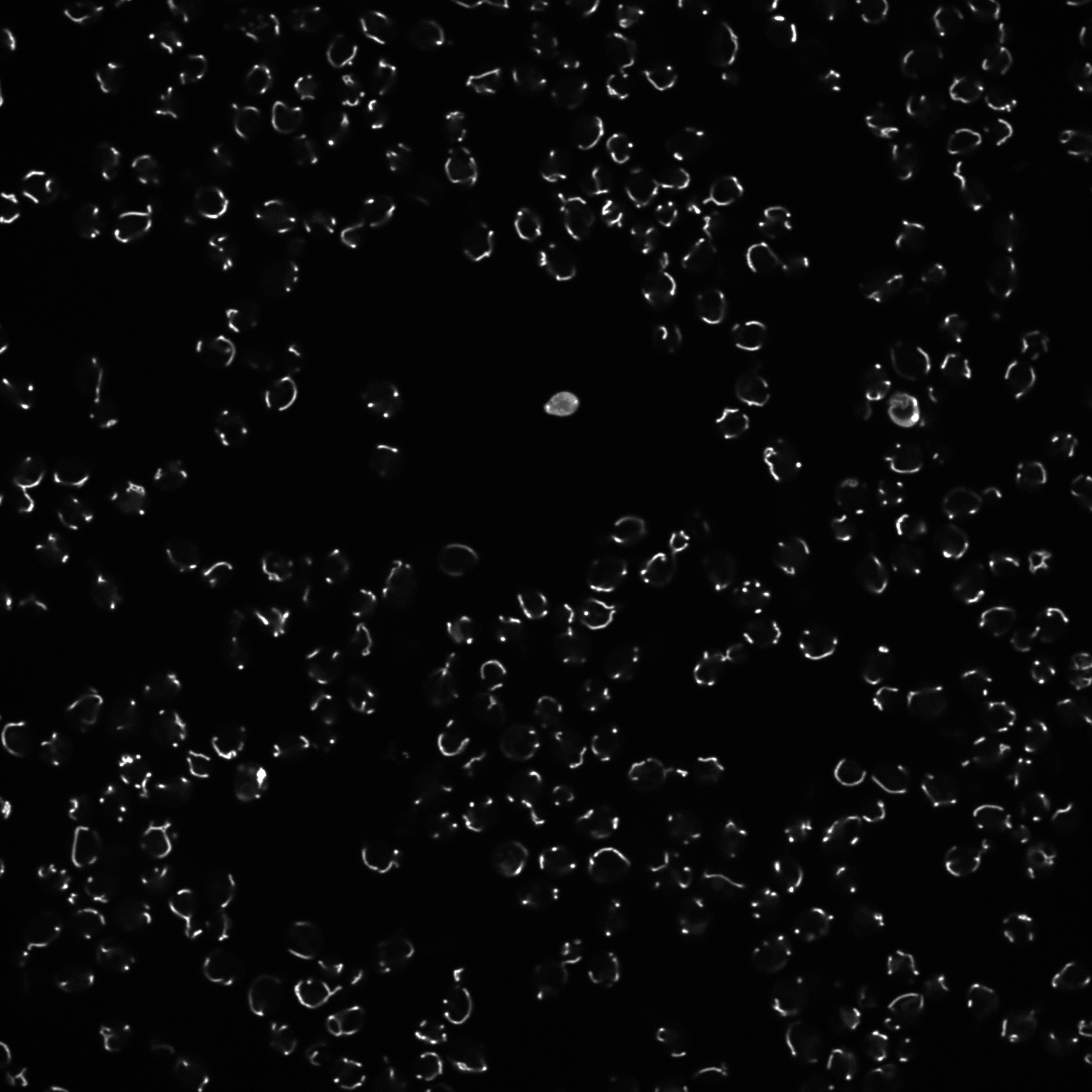

Supplement: Figure 2—source data 2. — The complete image dataset used for quantification is available on the BioImage Archive accession number S-BIAD2409. [file elife-98889-fig2-data2.zip › SourceData-Fig2B_splitGFP/Dual_no_mito/ARC1/GFP11/E15--W00111--P00001--Z00000--T00000--561nm.tif]

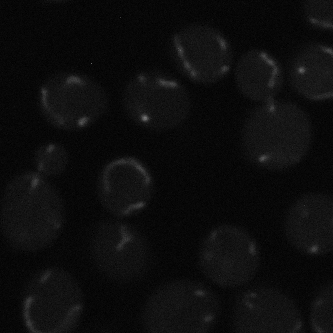

Supplement: Figure 2—source data 2. — The complete image dataset used for quantification is available on the BioImage Archive accession number S-BIAD2409. [file elife-98889-fig2-data2.zip › SourceData-Fig2B_splitGFP/Dual_no_mito/ARC1/GFP11/ARC1_GFP11.tif]

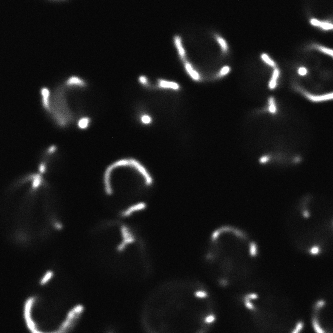

Supplement: Figure 2—source data 2. — The complete image dataset used for quantification is available on the BioImage Archive accession number S-BIAD2409. [file elife-98889-fig2-data2.zip › SourceData-Fig2B_splitGFP/Dual_no_mito/ARC1/GFP11/ARC1_mCH.png]

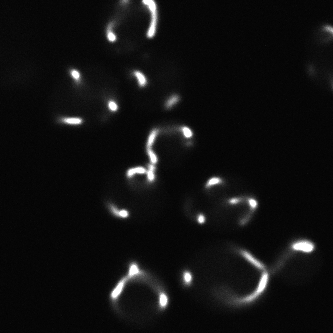

Supplement: Figure 2—source data 2. — The complete image dataset used for quantification is available on the BioImage Archive accession number S-BIAD2409. [file elife-98889-fig2-data2.zip › SourceData-Fig2B_splitGFP/Dual_with_strong_mito/FUM1/GFP11/FUM1_mCH.png]

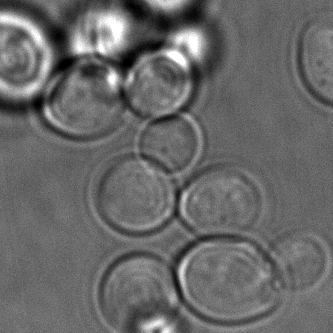

Supplement: Figure 2—source data 2. — The complete image dataset used for quantification is available on the BioImage Archive accession number S-BIAD2409. [file elife-98889-fig2-data2.zip › SourceData-Fig2B_splitGFP/Dual_with_strong_mito/FUM1/GFP11/FUM1_GFP11_Tr.png]

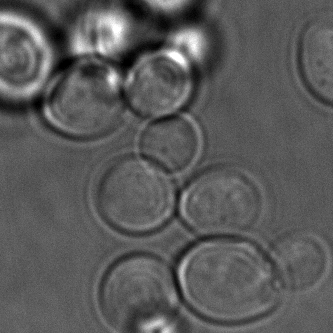

Supplement: Figure 2—source data 2. — The complete image dataset used for quantification is available on the BioImage Archive accession number S-BIAD2409. [file elife-98889-fig2-data2.zip › SourceData-Fig2B_splitGFP/Dual_with_strong_mito/FUM1/GFP11/FUM1_GFP11_Tr.tif]

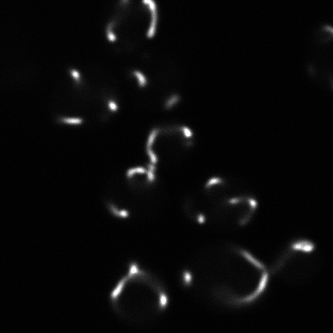

Supplement: Figure 2—source data 2. — The complete image dataset used for quantification is available on the BioImage Archive accession number S-BIAD2409. [file elife-98889-fig2-data2.zip › SourceData-Fig2B_splitGFP/Dual_with_strong_mito/FUM1/GFP11/FUM1_mCH.tif]

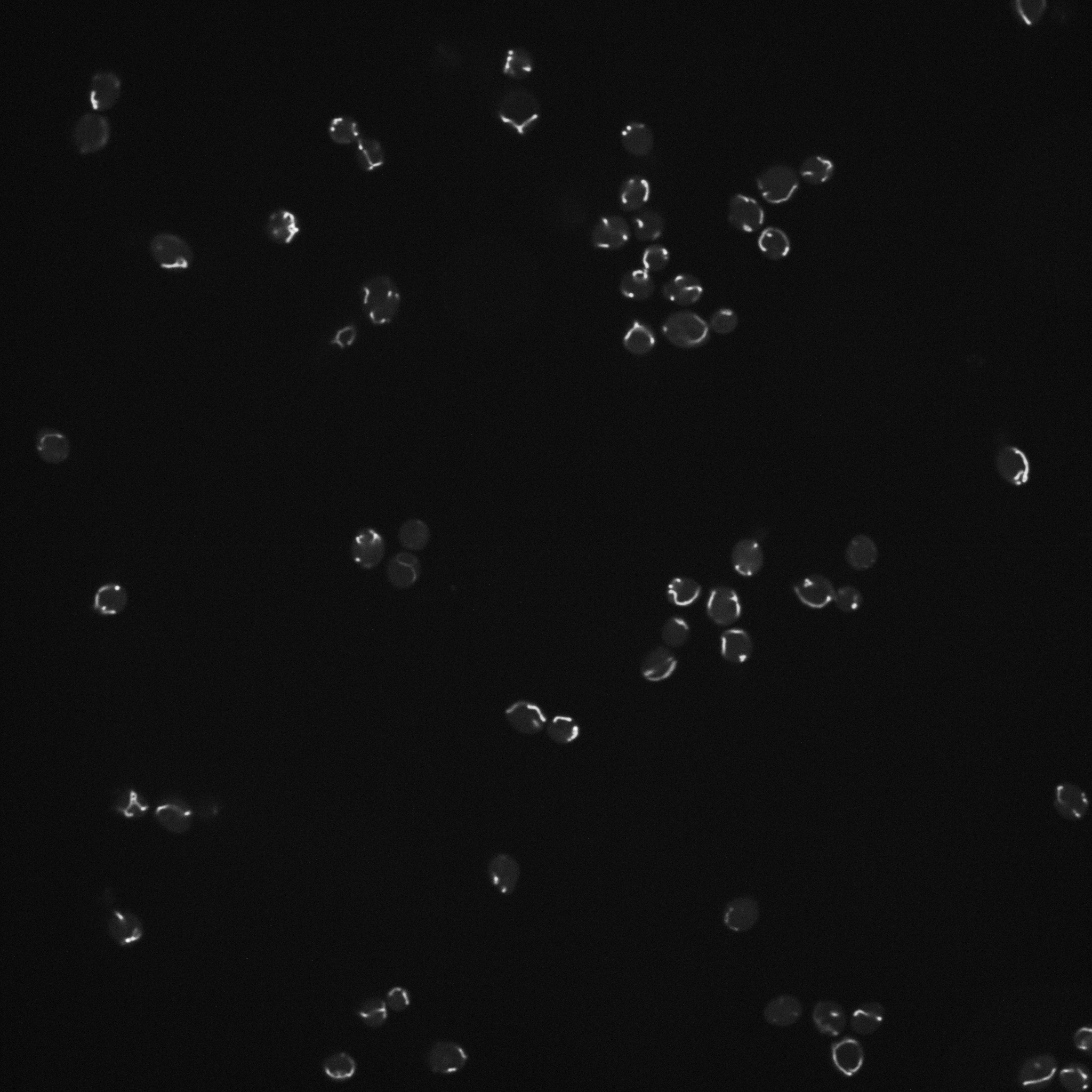

Supplement: Figure 2—source data 2. — The complete image dataset used for quantification is available on the BioImage Archive accession number S-BIAD2409. [file elife-98889-fig2-data2.zip › SourceData-Fig2B_splitGFP/Dual_with_strong_mito/FUM1/GFP11/B7--W00031--P00002--Z00000--T00000--488nm.tif]

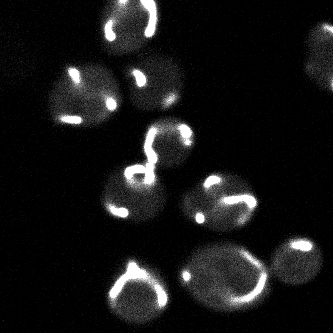

Supplement: Figure 2—source data 2. — The complete image dataset used for quantification is available on the BioImage Archive accession number S-BIAD2409. [file elife-98889-fig2-data2.zip › SourceData-Fig2B_splitGFP/Dual_with_strong_mito/FUM1/GFP11/FUM1_GFP11.png]

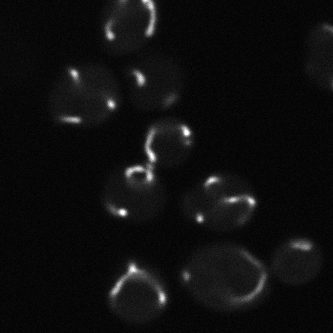

Supplement: Figure 2—source data 2. — The complete image dataset used for quantification is available on the BioImage Archive accession number S-BIAD2409. [file elife-98889-fig2-data2.zip › SourceData-Fig2B_splitGFP/Dual_with_strong_mito/FUM1/GFP11/FUM1_GFP11.tif]

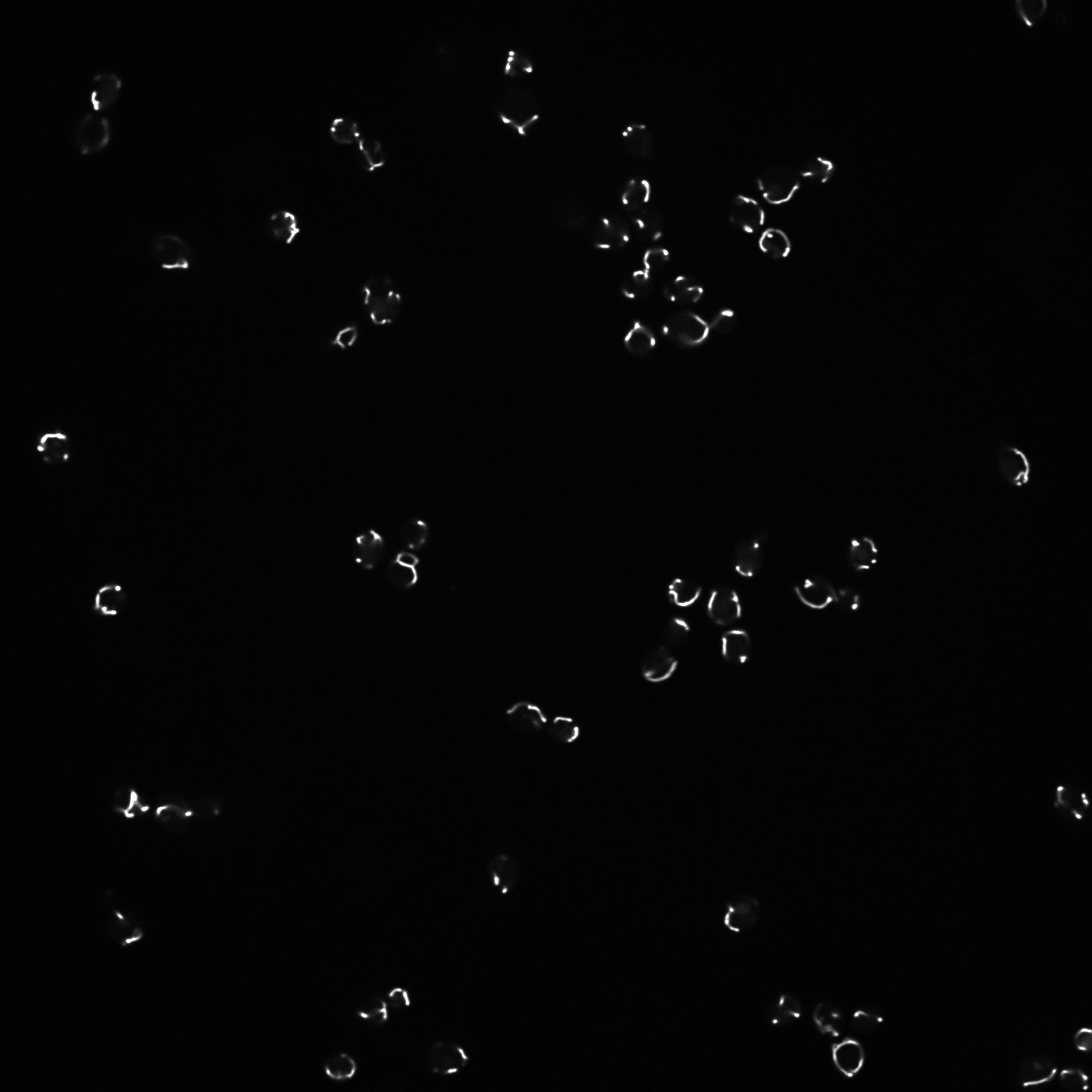

Supplement: Figure 2—source data 2. — The complete image dataset used for quantification is available on the BioImage Archive accession number S-BIAD2409. [file elife-98889-fig2-data2.zip › SourceData-Fig2B_splitGFP/Dual_with_strong_mito/FUM1/GFP11/B7--W00031--P00002--Z00000--T00000--561nm.tif]

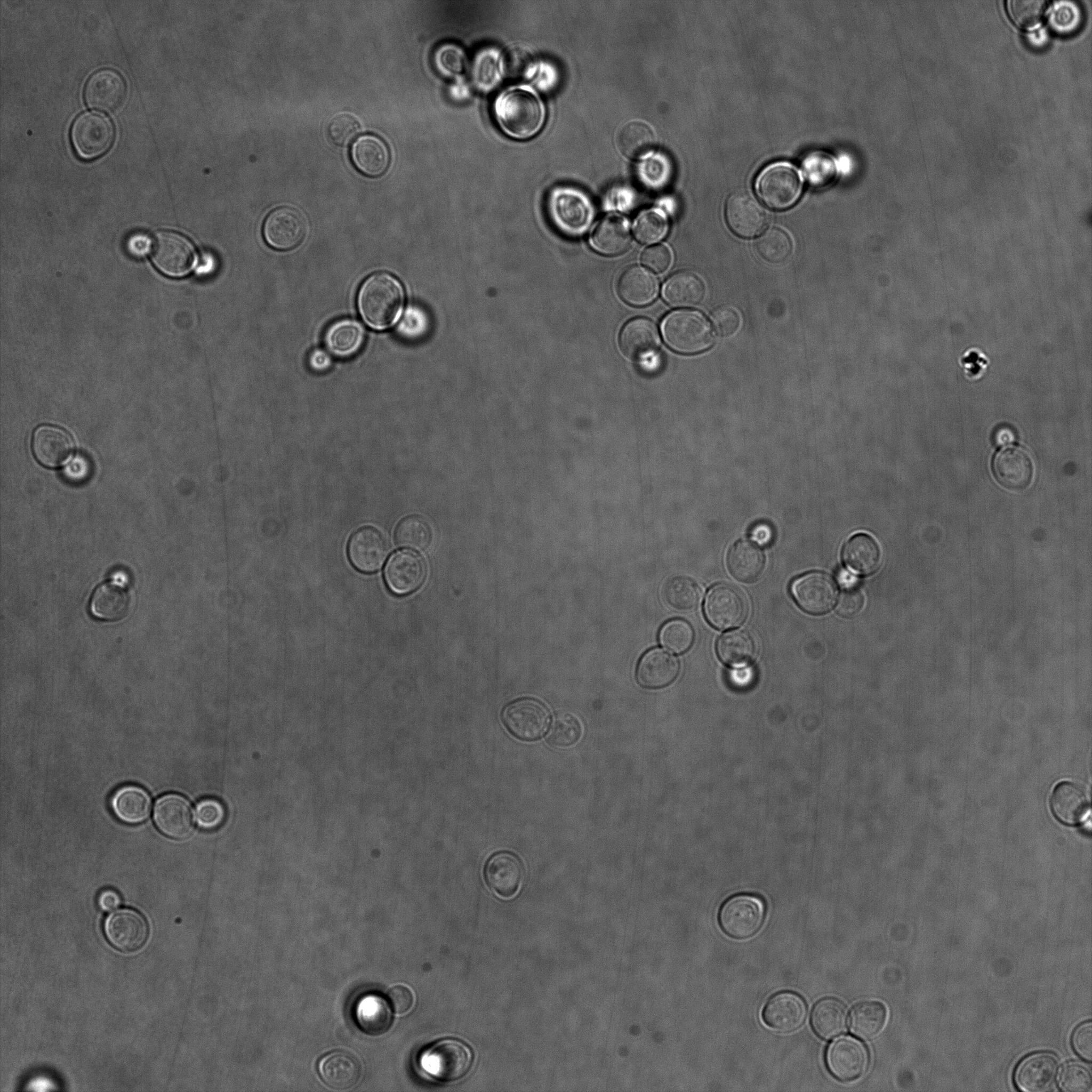

Supplement: Figure 2—source data 2. — The complete image dataset used for quantification is available on the BioImage Archive accession number S-BIAD2409. [file elife-98889-fig2-data2.zip › SourceData-Fig2B_splitGFP/Dual_with_strong_mito/FUM1/GFP11/B7--W00031--P00002--Z00000--T00000--TransCon.tif]

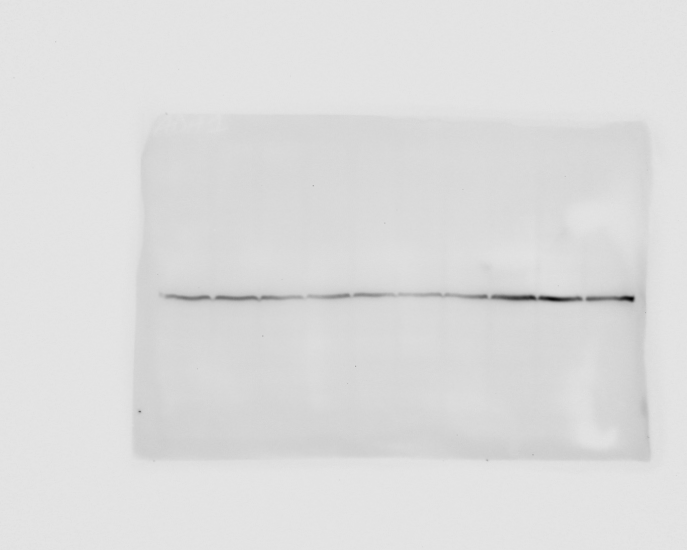

Supplement: Figure 3—figure supplement 2—source data 2. [file elife-98889-fig3-figsupp2-data2.zip › Fig3supp2-sourcedata2/ADH1 ECL.tif]

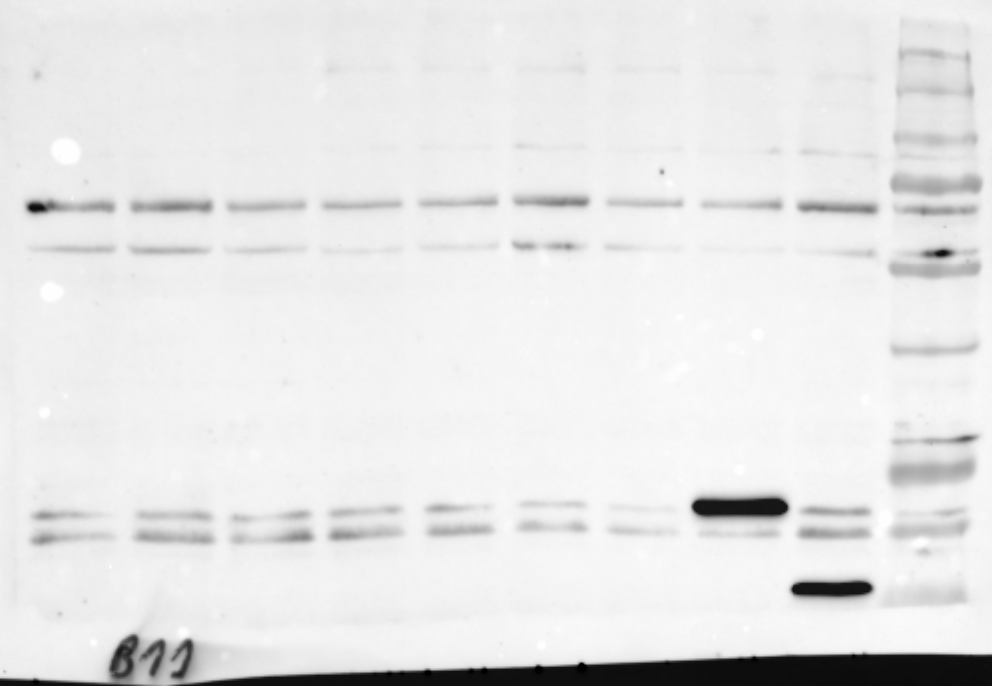

Supplement: Figure 3—figure supplement 2—source data 2. [file elife-98889-fig3-figsupp2-data2.zip › Fig3supp2-sourcedata2/B11 merge.tif]

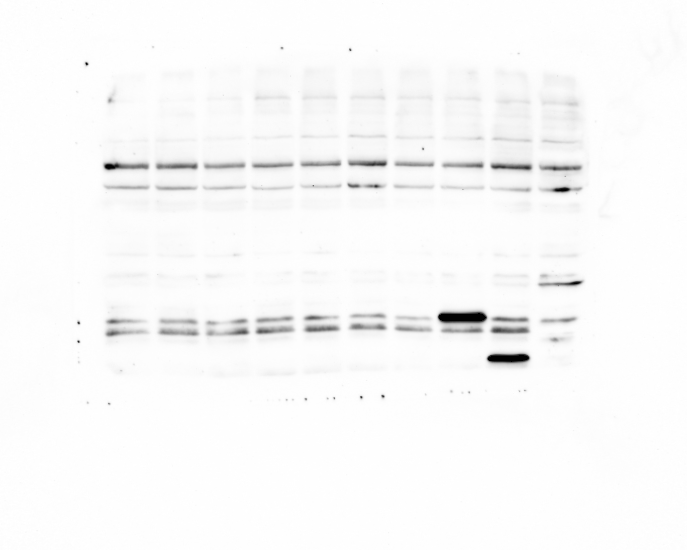

Supplement: Figure 3—figure supplement 2—source data 2. [file elife-98889-fig3-figsupp2-data2.zip › Fig3supp2-sourcedata2/B11 ECL.tif]

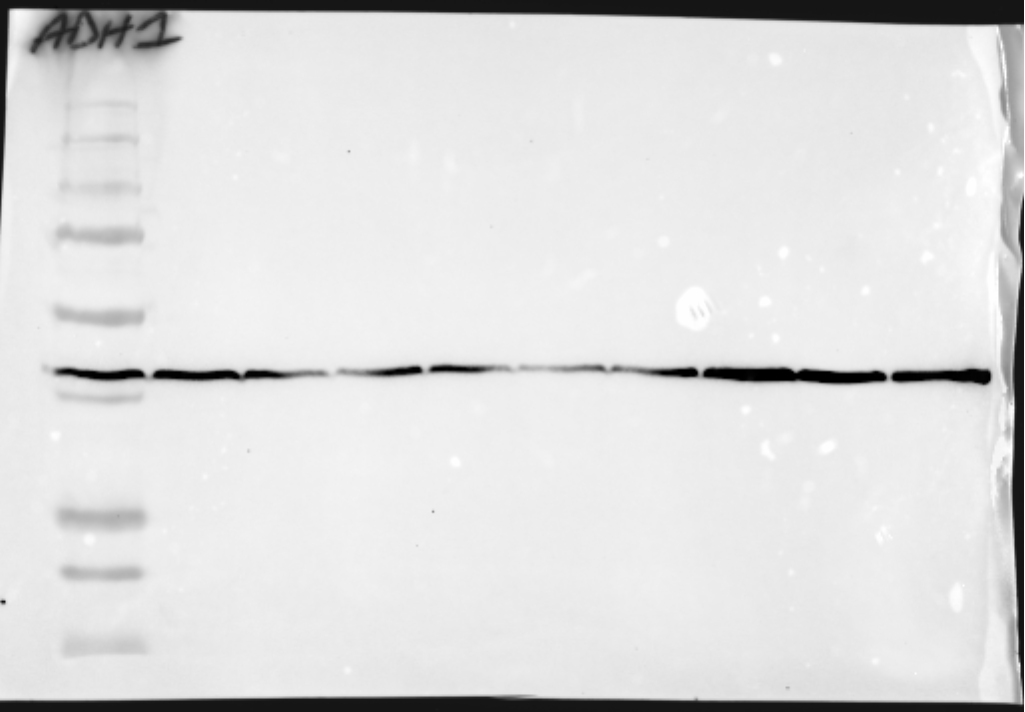

Supplement: Figure 3—figure supplement 2—source data 2. [file elife-98889-fig3-figsupp2-data2.zip › Fig3supp2-sourcedata2/ADH1 merge.tif]

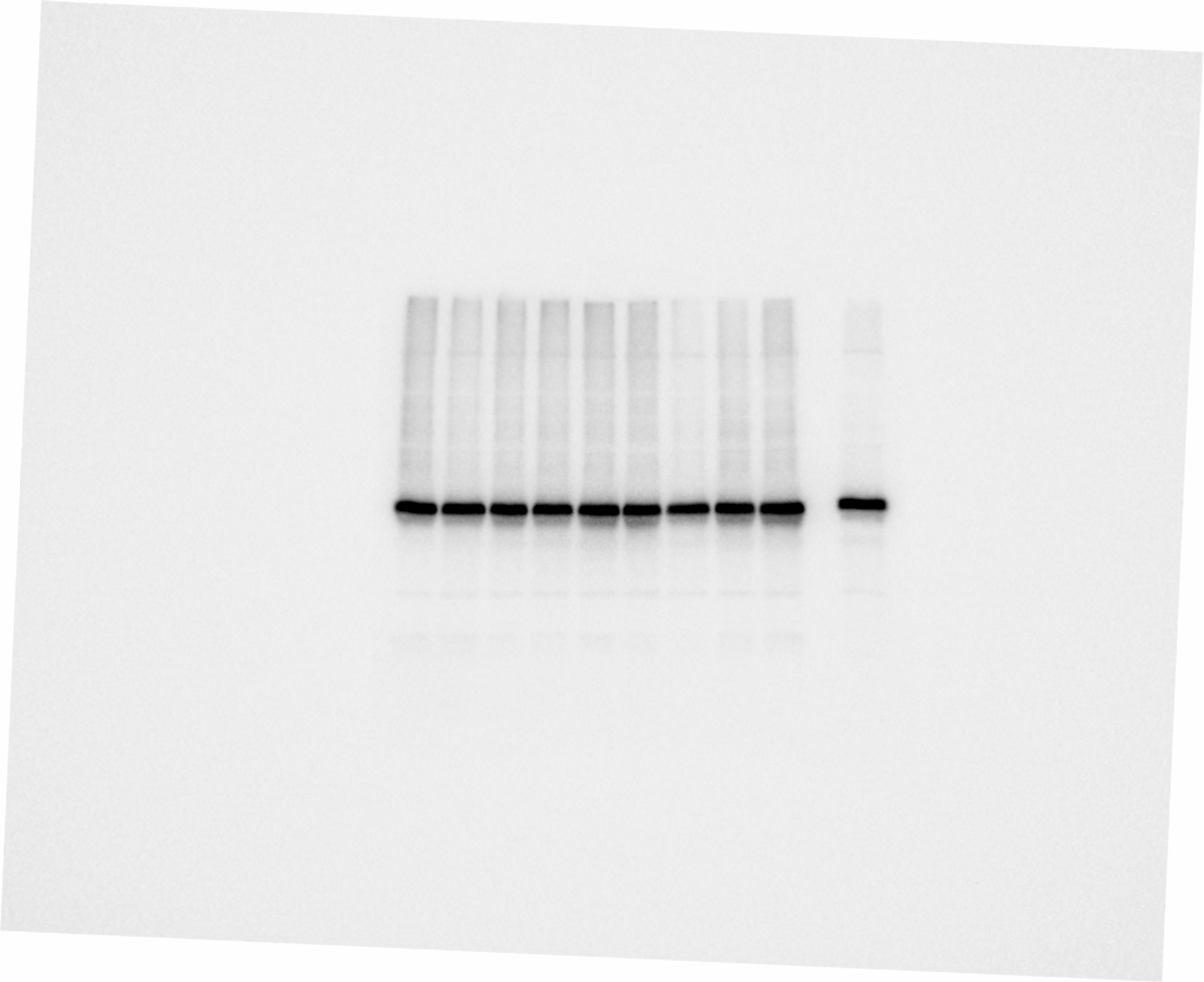

Supplement: Figure 4—source data 2. [file elife-98889-fig4-data2.zip › Fig4sourcedata2/ADH1 ECL.tif]

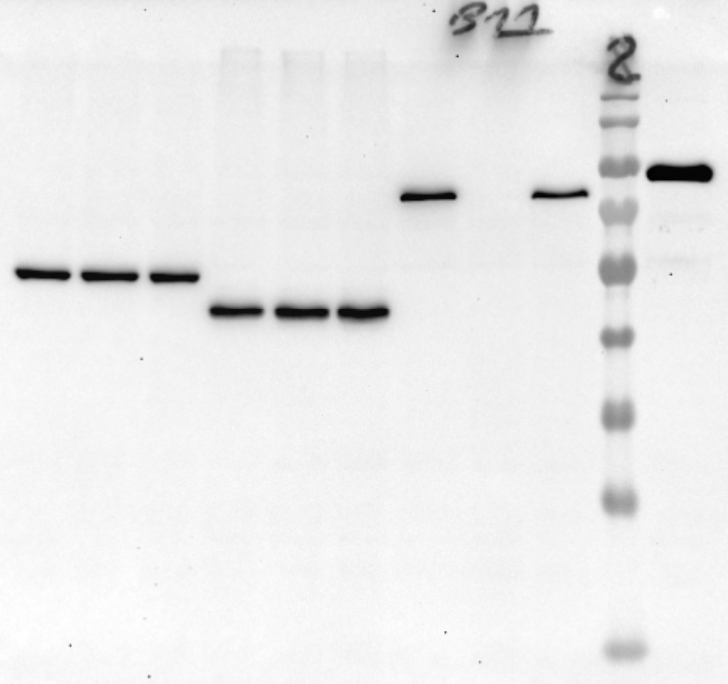

Supplement: Figure 4—source data 2. [file elife-98889-fig4-data2.zip › Fig4sourcedata2/B11 merge.tif]

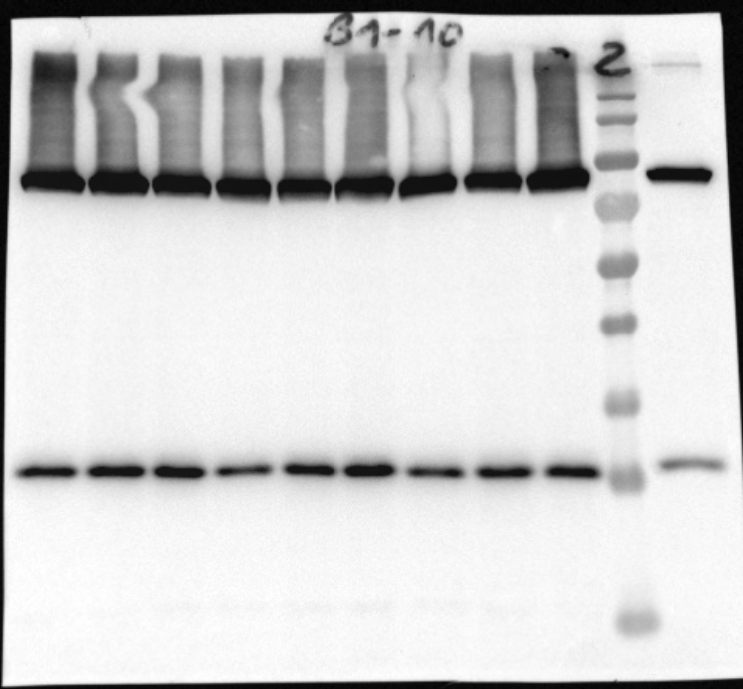

Supplement: Figure 4—source data 2. [file elife-98889-fig4-data2.zip › Fig4sourcedata2/B1-10 merge.tif]

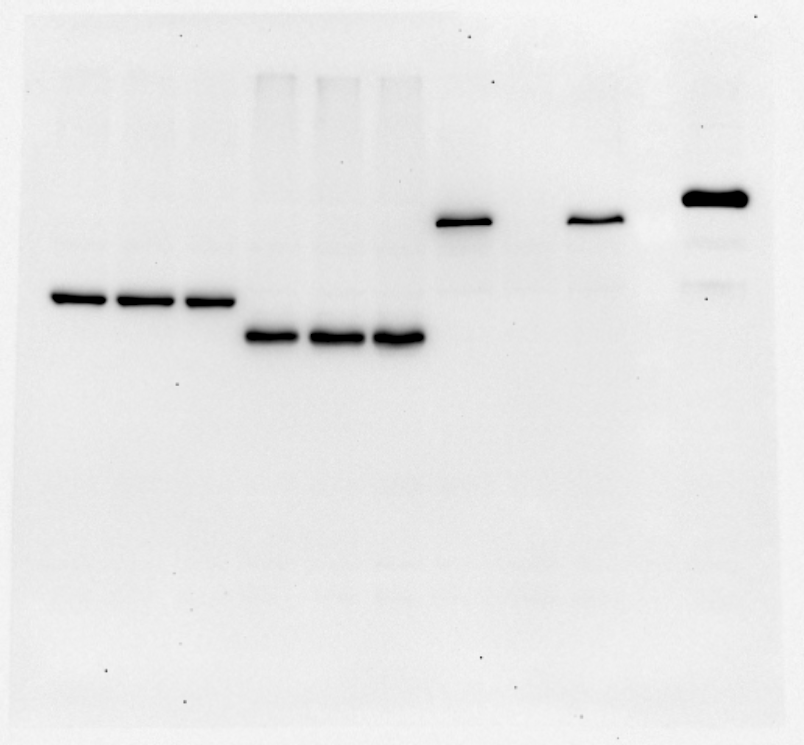

Supplement: Figure 4—source data 2. [file elife-98889-fig4-data2.zip › Fig4sourcedata2/B11 ECL.tif]

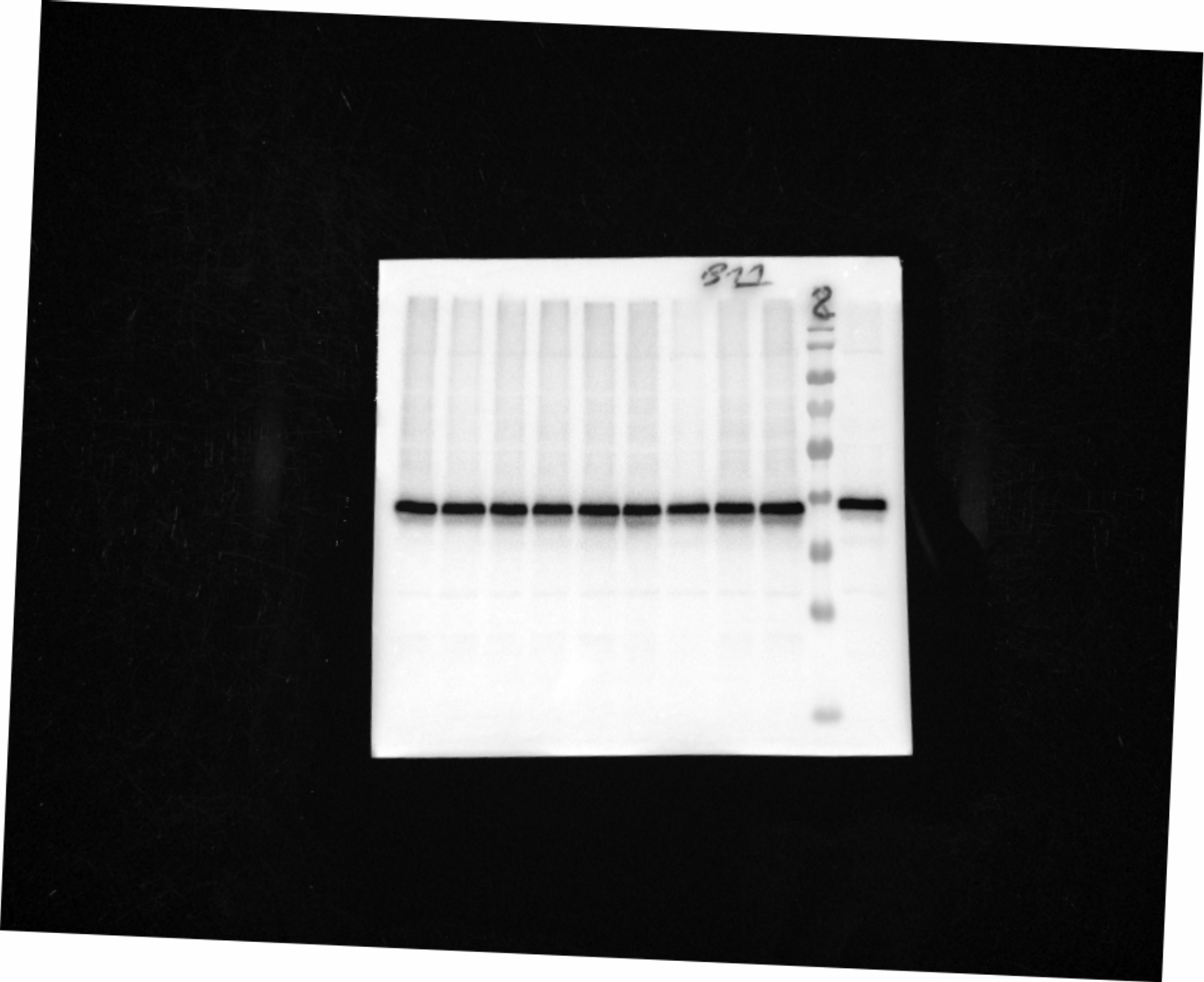

Supplement: Figure 4—source data 2. [file elife-98889-fig4-data2.zip › Fig4sourcedata2/ADH1 merge.tif]

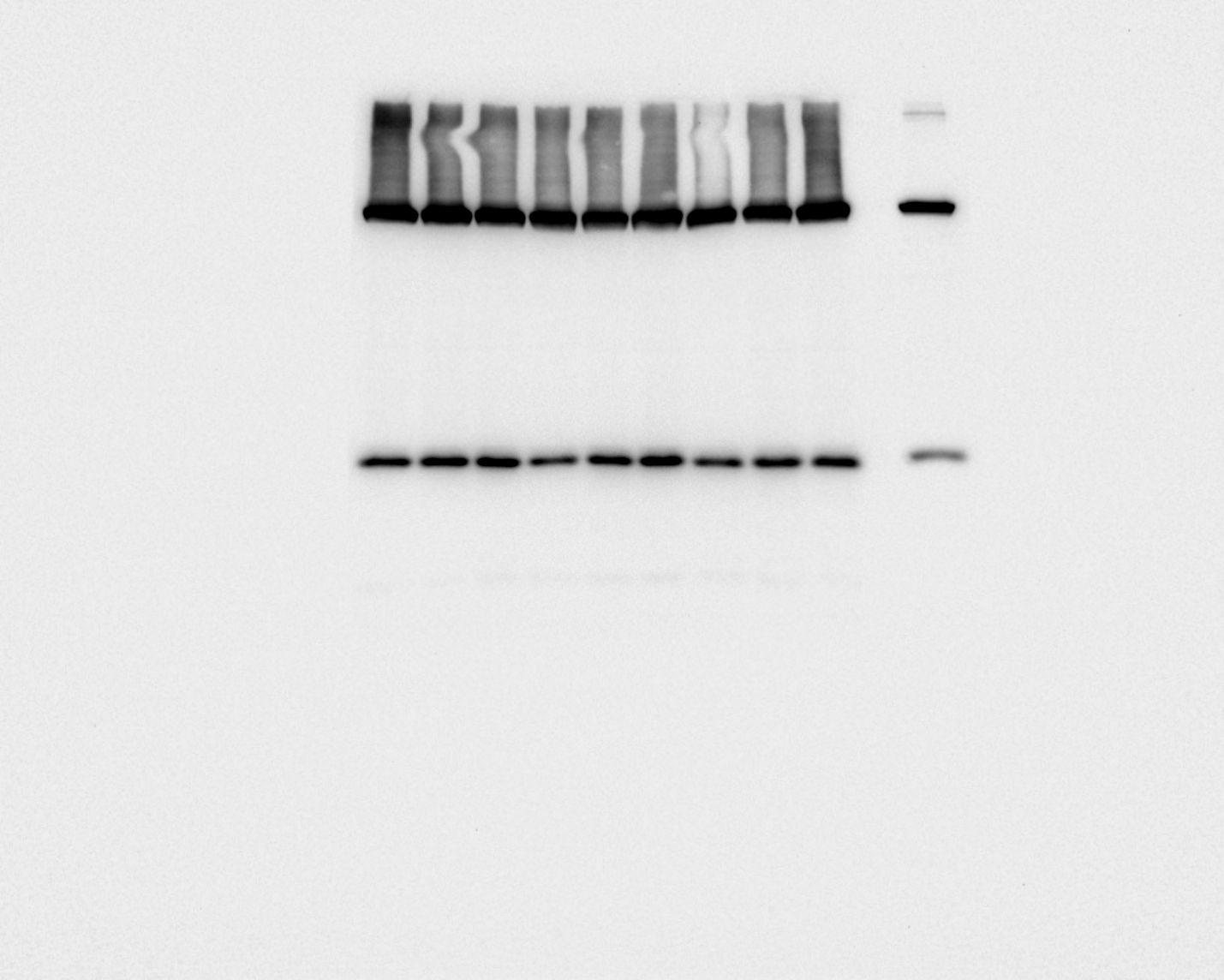

Supplement: Figure 4—source data 2. [file elife-98889-fig4-data2.zip › Fig4sourcedata2/B1-10 ECL.tif]

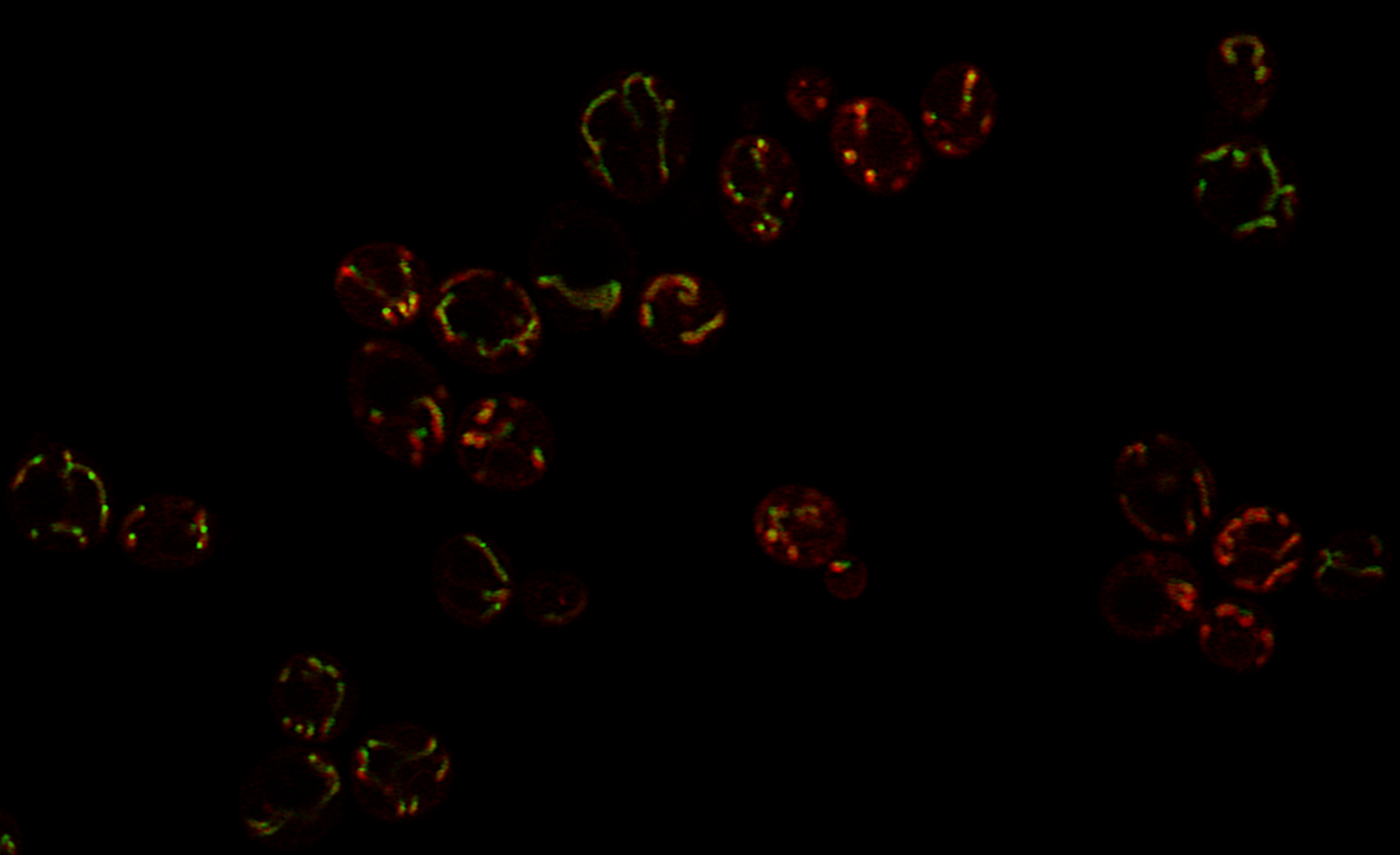

Supplement: Figure 4—source data 3. [file elife-98889-fig4-data3.zip › SourceData-Fig4D/BigMitoSplit GFP CHA1-B11 MitotrackRED-01-Airyscan Processing-03-Image Export-06/BigMitoSplit GFP CHA1-B11 MitotrackRED-01-Airyscan Processing-03-Image Export-06_c1-2.tif]

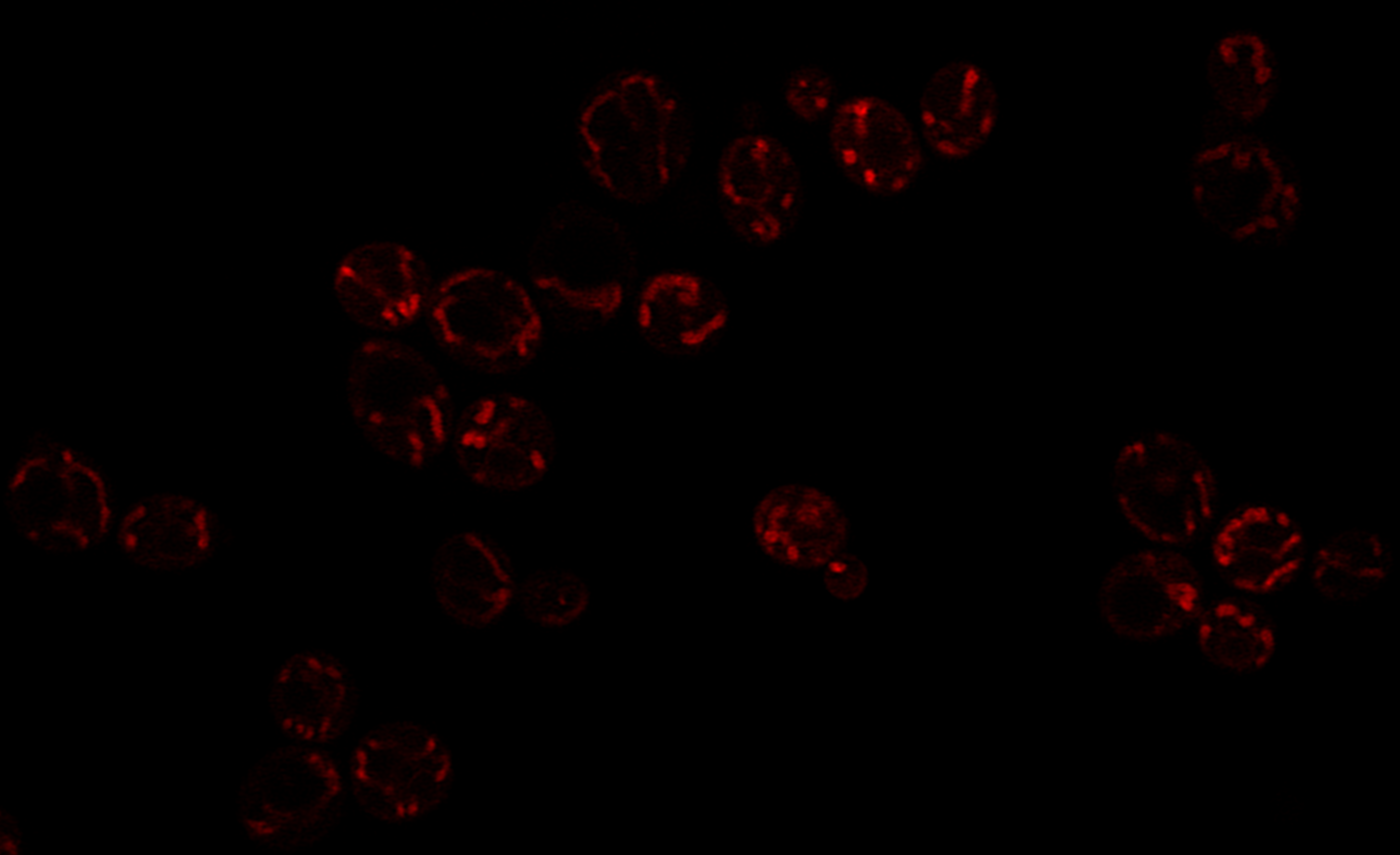

Supplement: Figure 4—source data 3. [file elife-98889-fig4-data3.zip › SourceData-Fig4D/BigMitoSplit GFP CHA1-B11 MitotrackRED-01-Airyscan Processing-03-Image Export-06/BigMitoSplit GFP CHA1-B11 MitotrackRED-01-Airyscan Processing-03-Image Export-06_c2.tif]

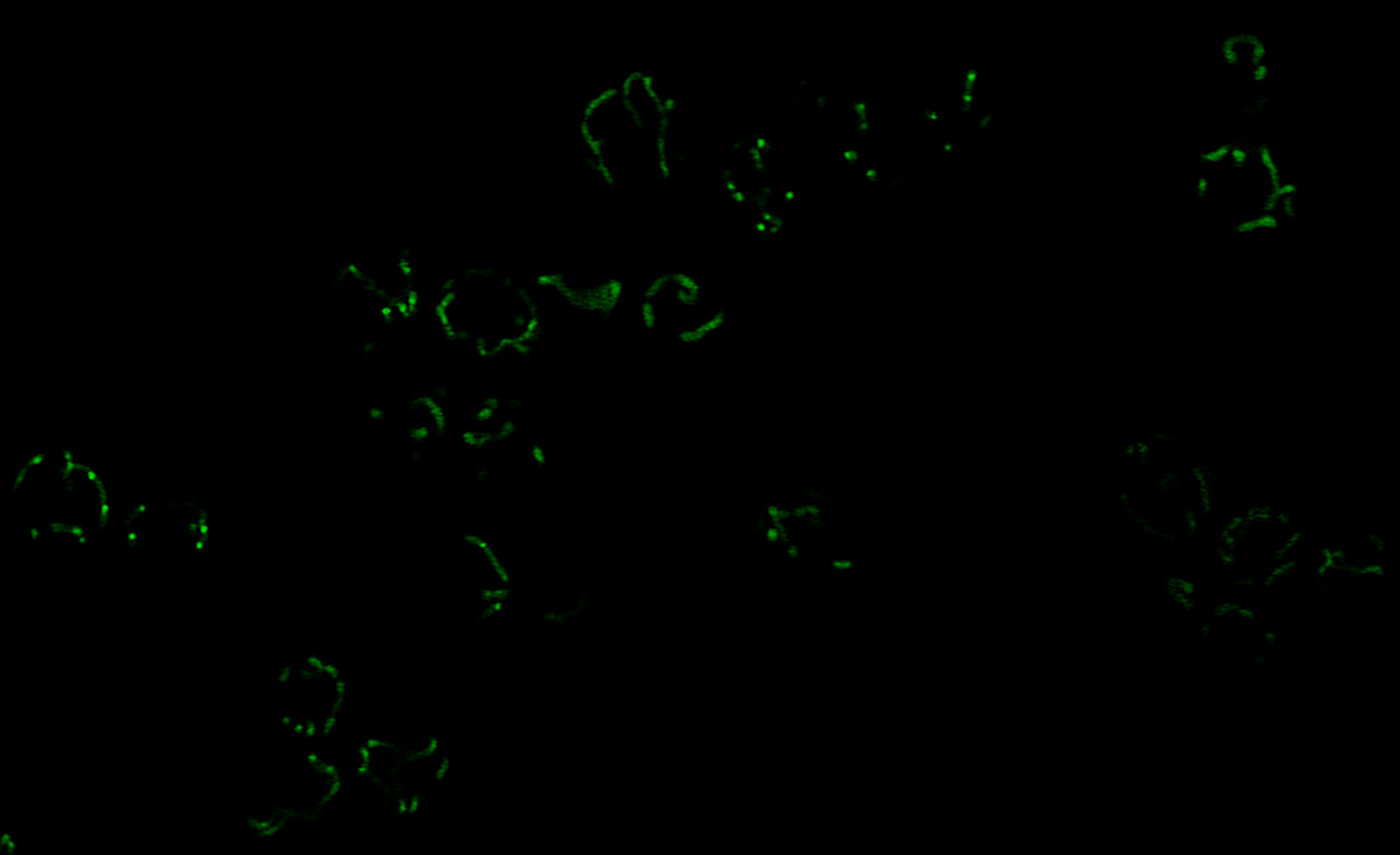

Supplement: Figure 4—source data 3. [file elife-98889-fig4-data3.zip › SourceData-Fig4D/BigMitoSplit GFP CHA1-B11 MitotrackRED-01-Airyscan Processing-03-Image Export-06/BigMitoSplit GFP CHA1-B11 MitotrackRED-01-Airyscan Processing-03-Image Export-06_c1.tif]

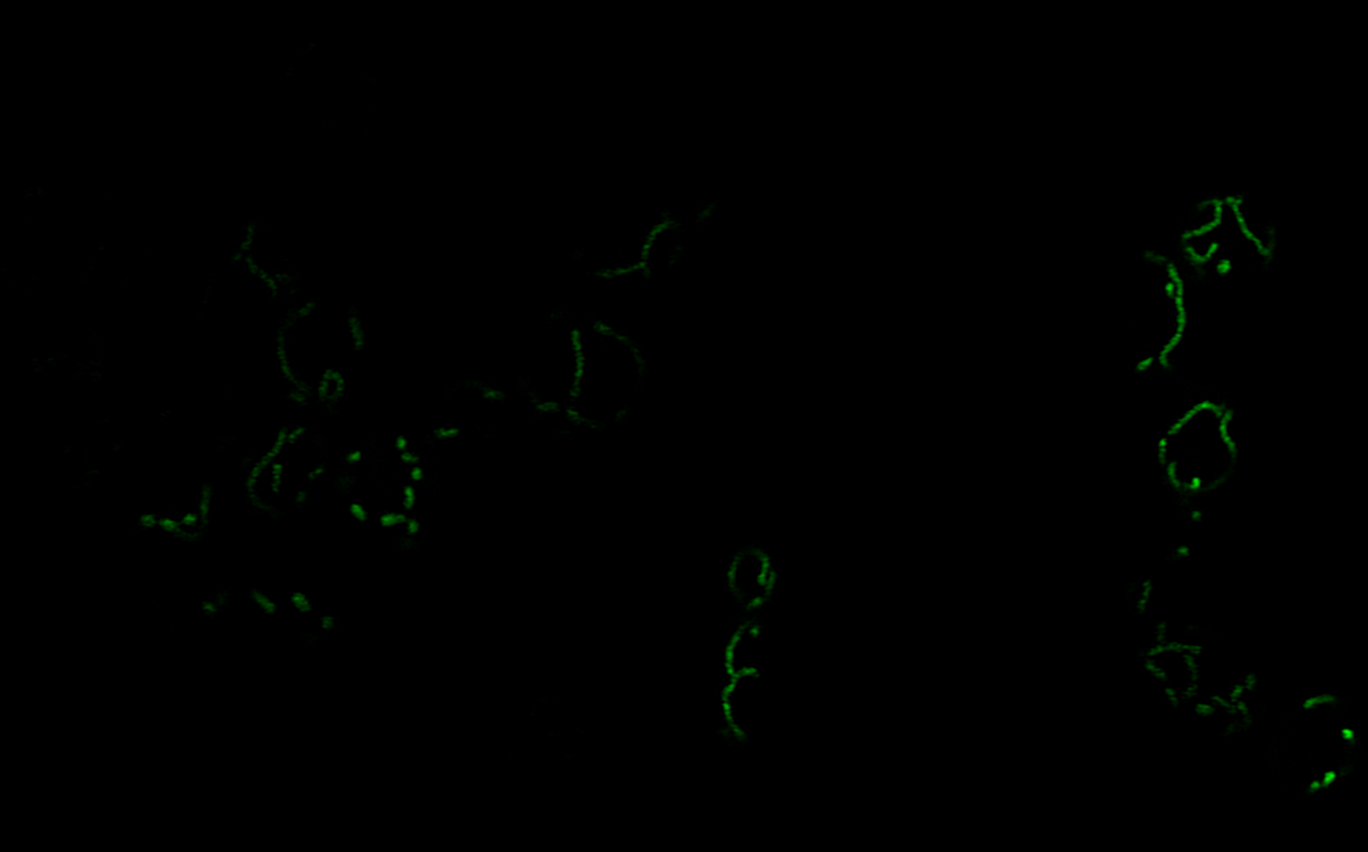

Supplement: Figure 4—source data 3. [file elife-98889-fig4-data3.zip › SourceData-Fig4D/BigMitoSplit GFP ARC1-B11 MitotrackRED-02-Airyscan Processing-02-Image Export-04/BigMitoSplit GFP ARC1-B11 MitotrackRED-02-Airyscan Processing-02-Image Export-04_c1.tif]

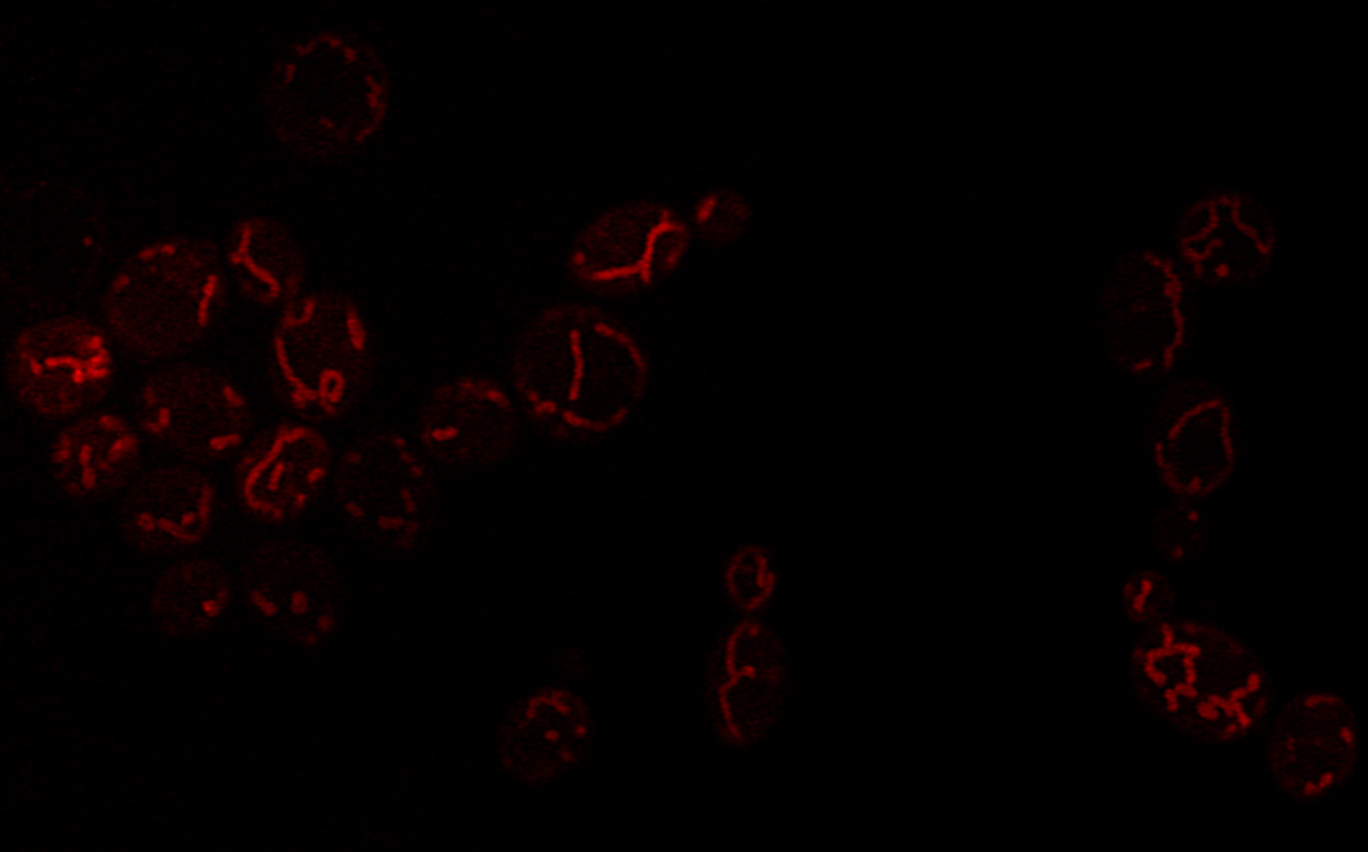

Supplement: Figure 4—source data 3. [file elife-98889-fig4-data3.zip › SourceData-Fig4D/BigMitoSplit GFP ARC1-B11 MitotrackRED-02-Airyscan Processing-02-Image Export-04/BigMitoSplit GFP ARC1-B11 MitotrackRED-02-Airyscan Processing-02-Image Export-04_c2.tif]

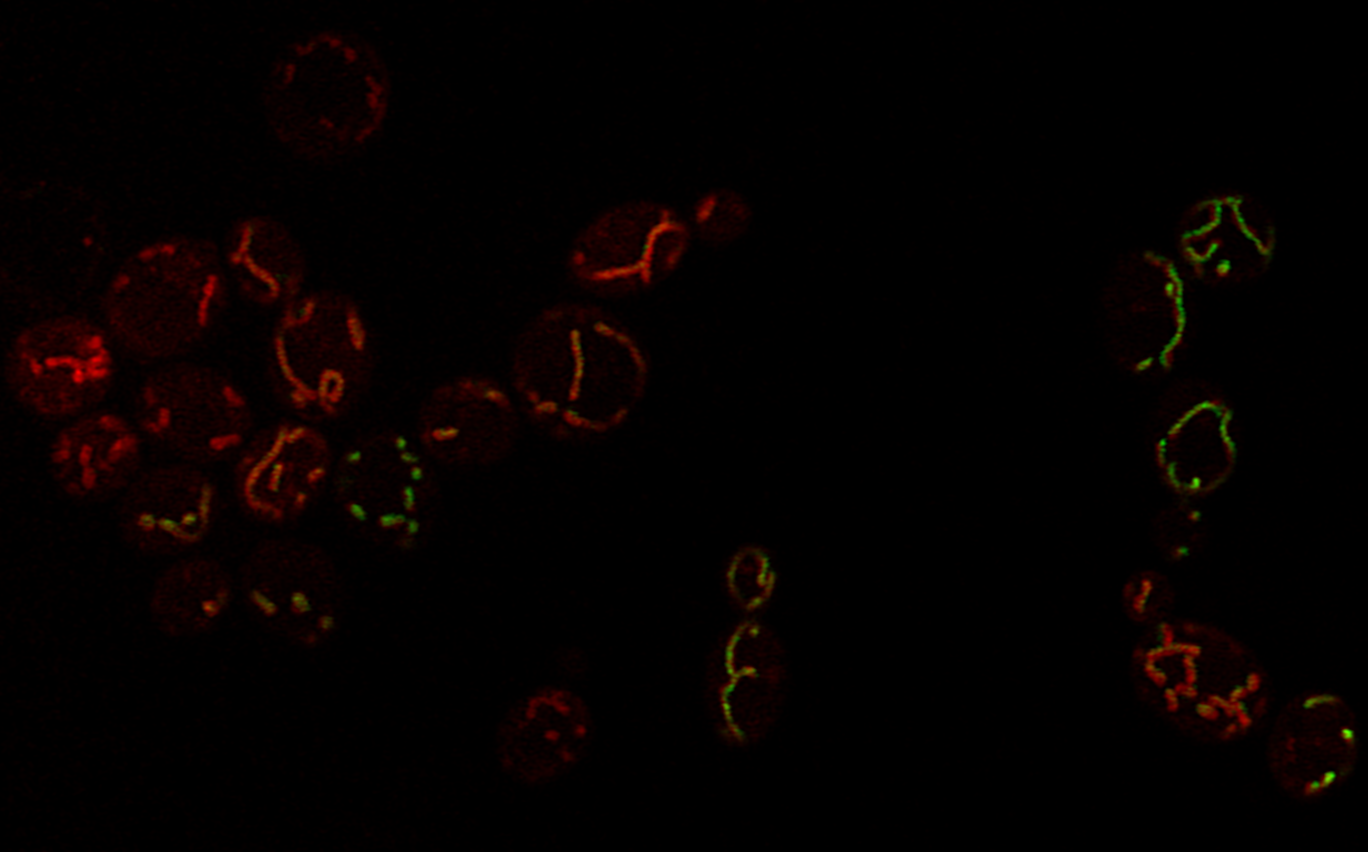

Supplement: Figure 4—source data 3. [file elife-98889-fig4-data3.zip › SourceData-Fig4D/BigMitoSplit GFP ARC1-B11 MitotrackRED-02-Airyscan Processing-02-Image Export-04/BigMitoSplit GFP ARC1-B11 MitotrackRED-02-Airyscan Processing-02-Image Export-04_c1-2.tif]

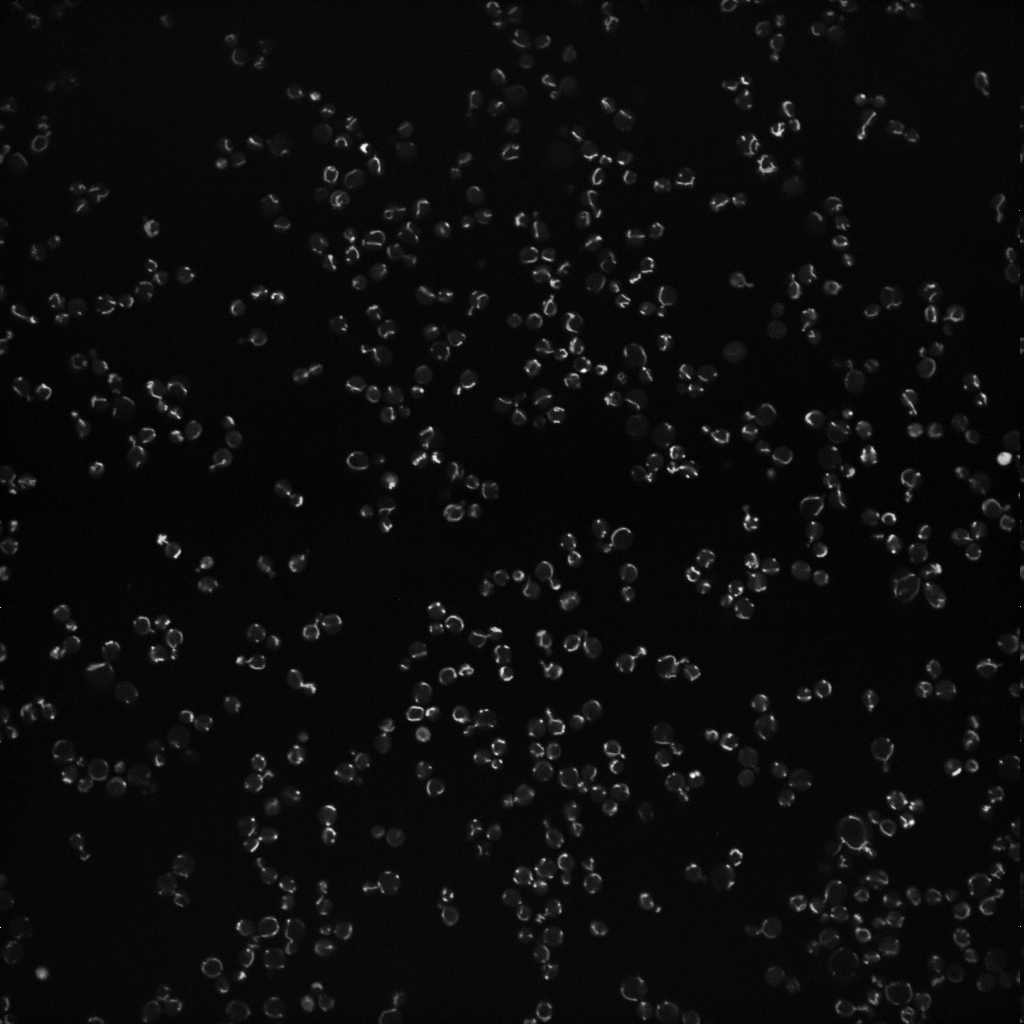

Supplement: Figure 4—source data 4. [file elife-98889-fig4-data4.zip › SourceData-Fig4F/GPP1-NATIVEpr/GPP1_NaCl_log_repeat1_w2confGFP_s40.tif]

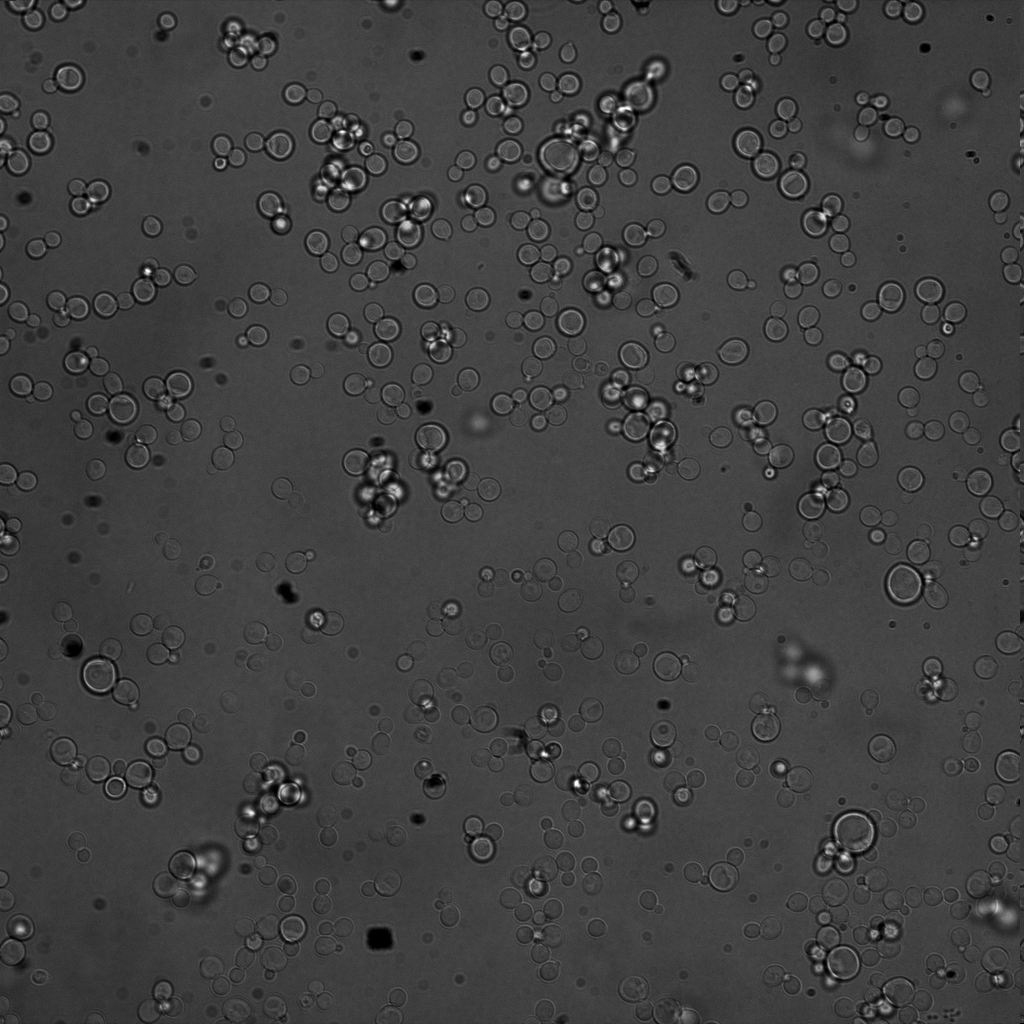

Supplement: Figure 4—source data 4. [file elife-98889-fig4-data4.zip › SourceData-Fig4F/GPP1-NATIVEpr/GPP1_NaCl_log_repeat1_w1Brightfield_s40.tif]

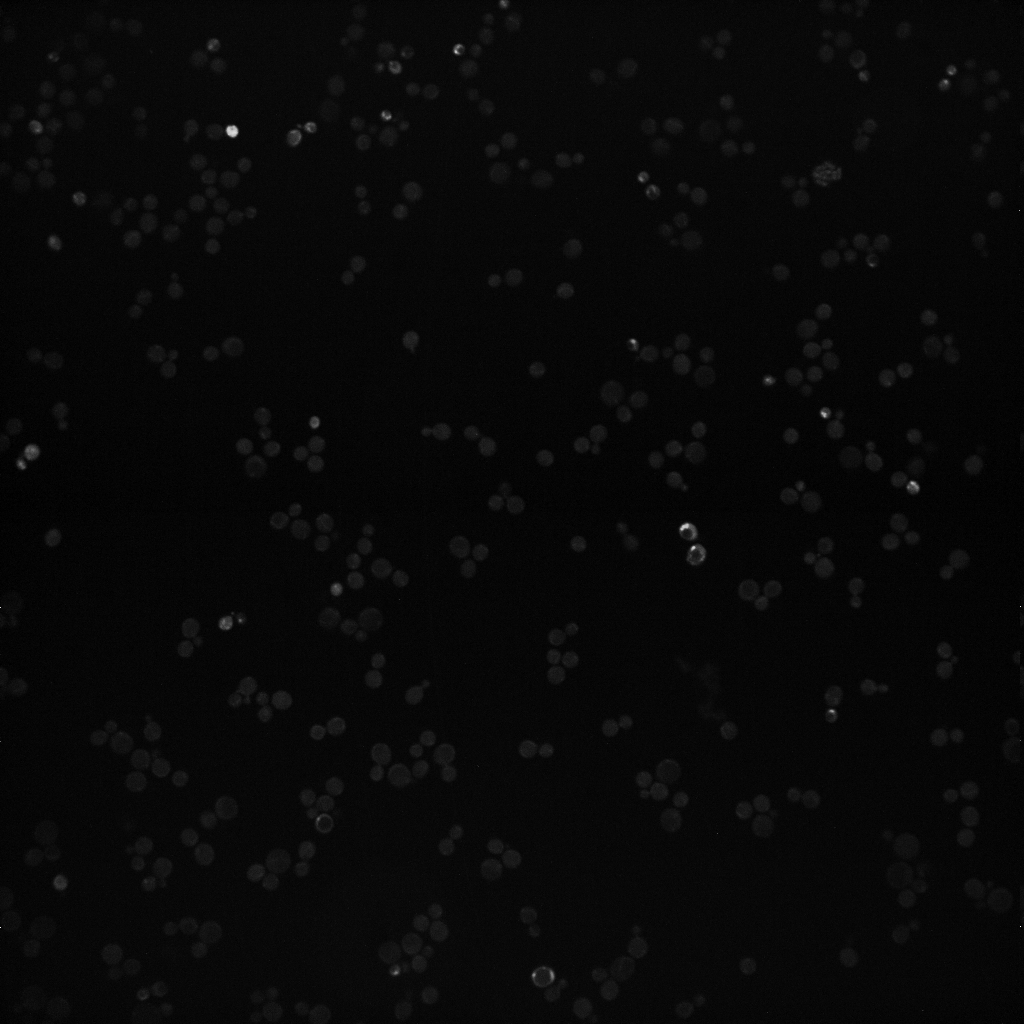

Supplement: Figure 4—source data 4. [file elife-98889-fig4-data4.zip › SourceData-Fig4F/GPP2-TEFpr-3HA/GPP1_NaCl_log_repeat1_w2confGFP_s56.tif]

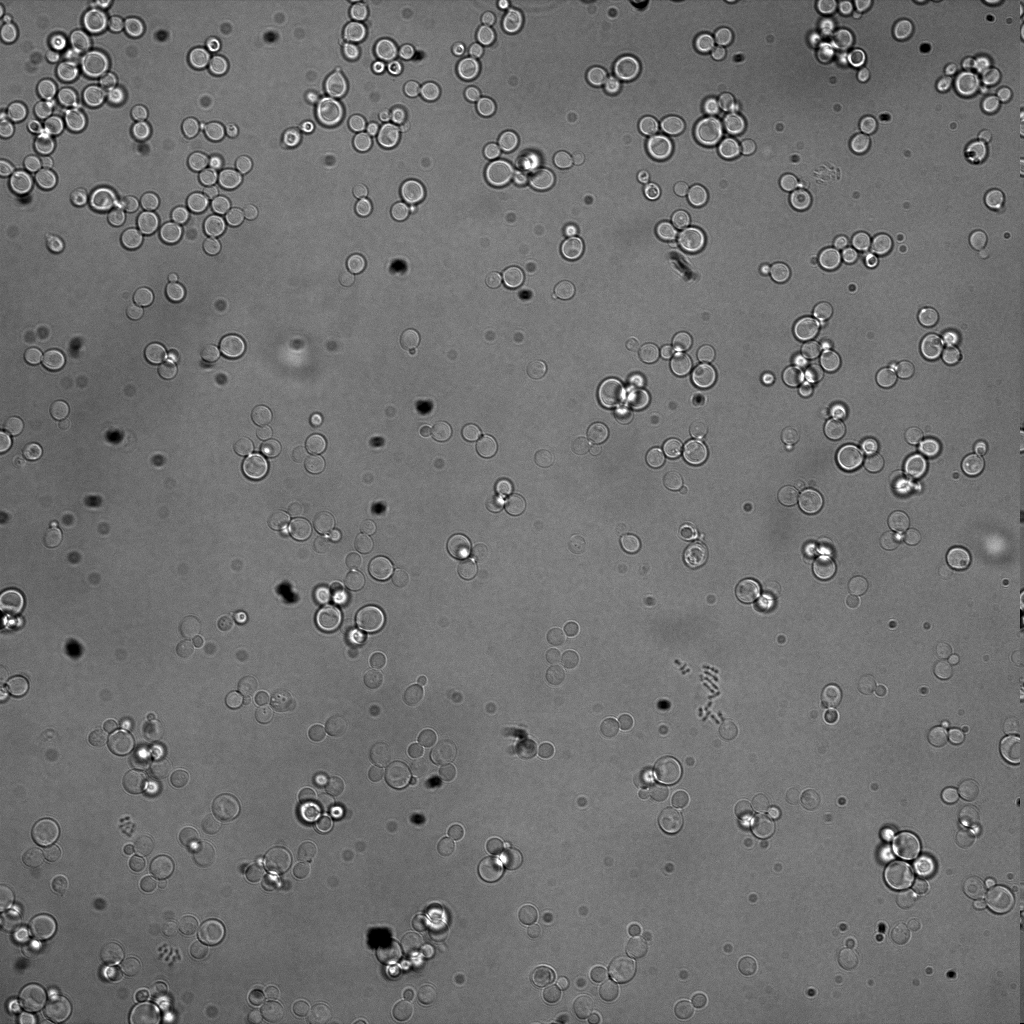

Supplement: Figure 4—source data 4. [file elife-98889-fig4-data4.zip › SourceData-Fig4F/GPP2-TEFpr-3HA/GPP1_NaCl_log_repeat1_w1Brightfield_s56.tif]

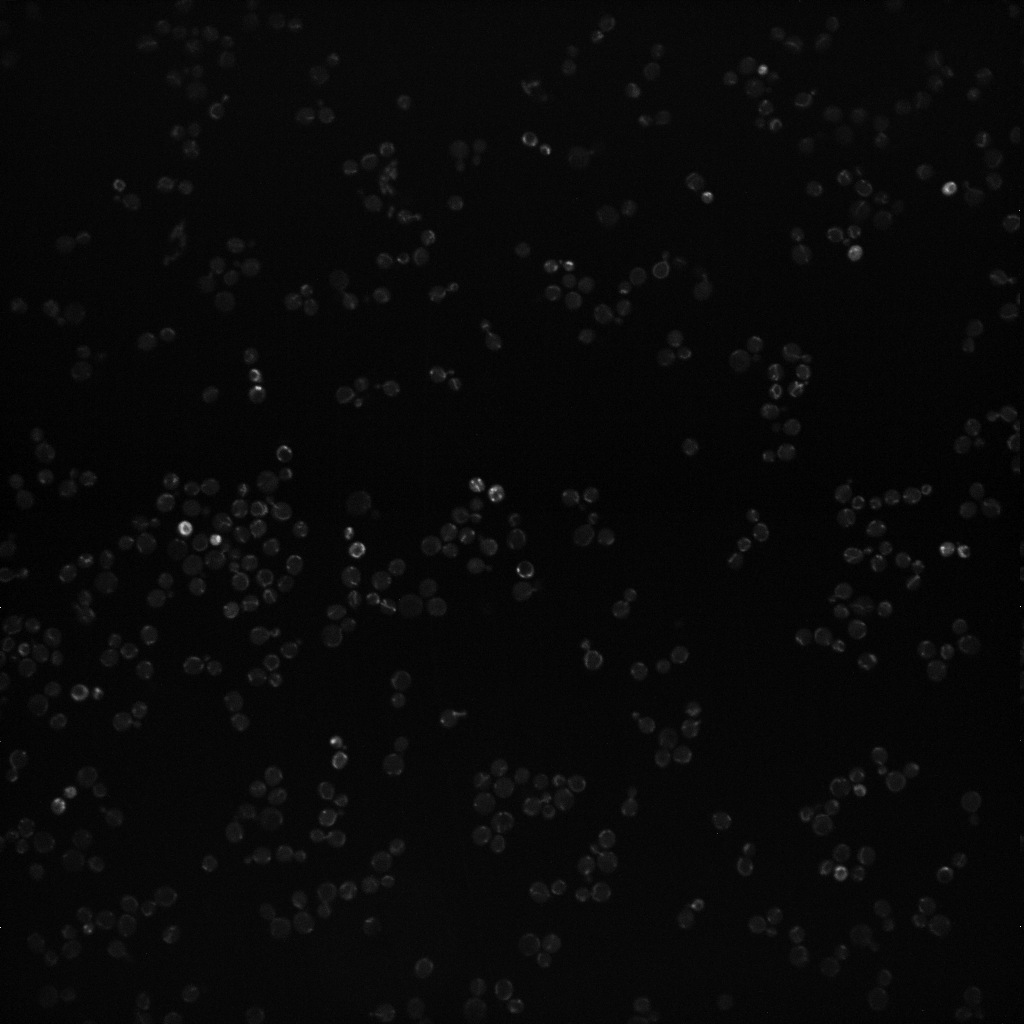

Supplement: Figure 4—source data 4. [file elife-98889-fig4-data4.zip › SourceData-Fig4F/GPP1-TEFpr/GPP1_NaCl_log_repeat1_w2confGFP_s4.tif]

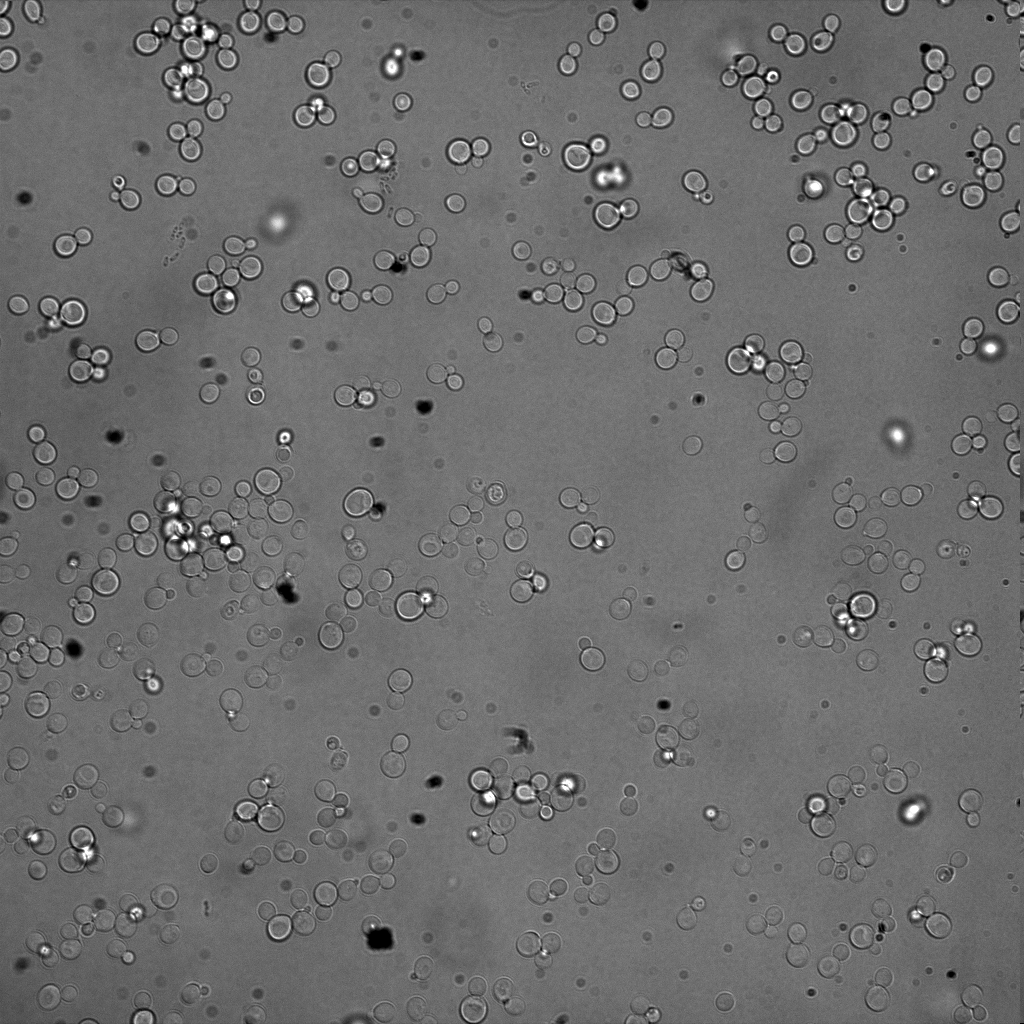

Supplement: Figure 4—source data 4. [file elife-98889-fig4-data4.zip › SourceData-Fig4F/GPP1-TEFpr/GPP1_NaCl_log_repeat1_w1Brightfield_s4.tif]

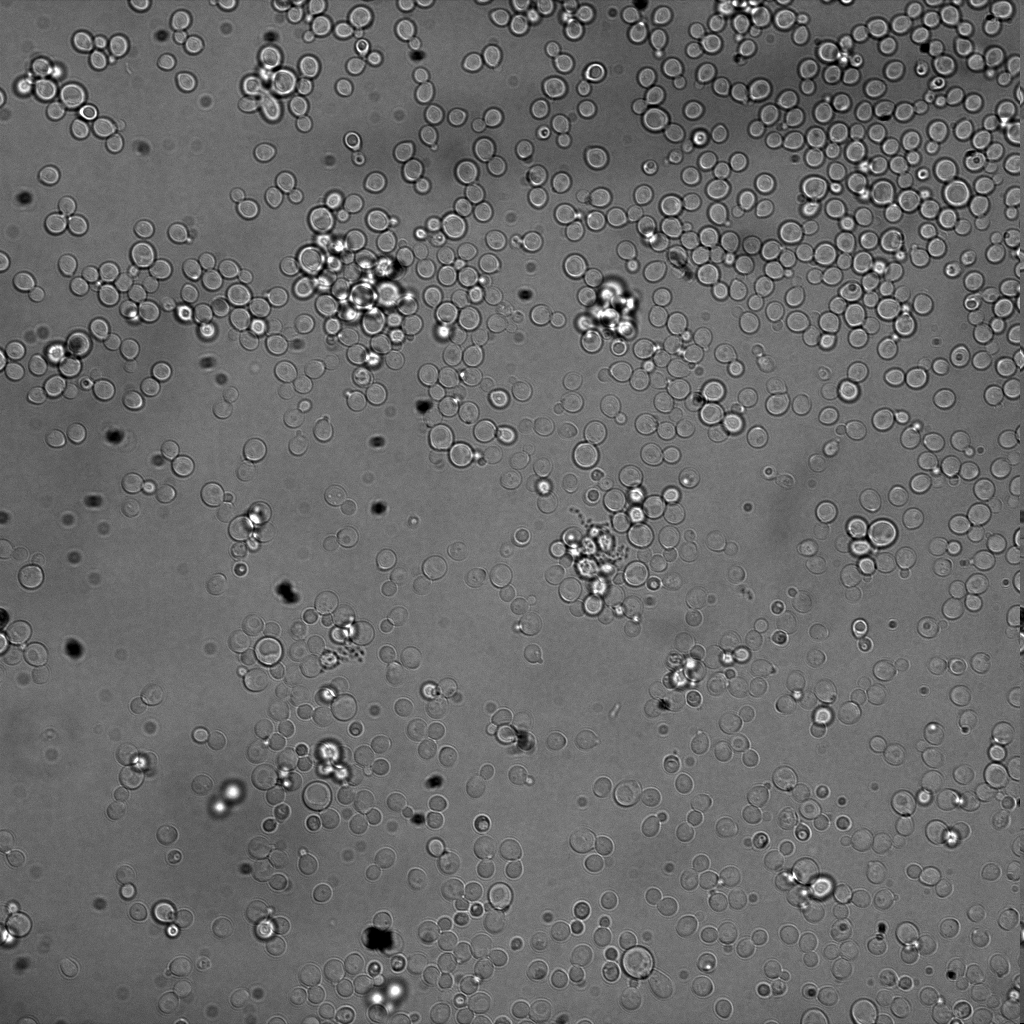

Supplement: Figure 4—source data 4. [file elife-98889-fig4-data4.zip › SourceData-Fig4F/GPP1-TEFpr-3HA/GPP1_NaCl_log_repeat1_w1Brightfield_s12.tif]

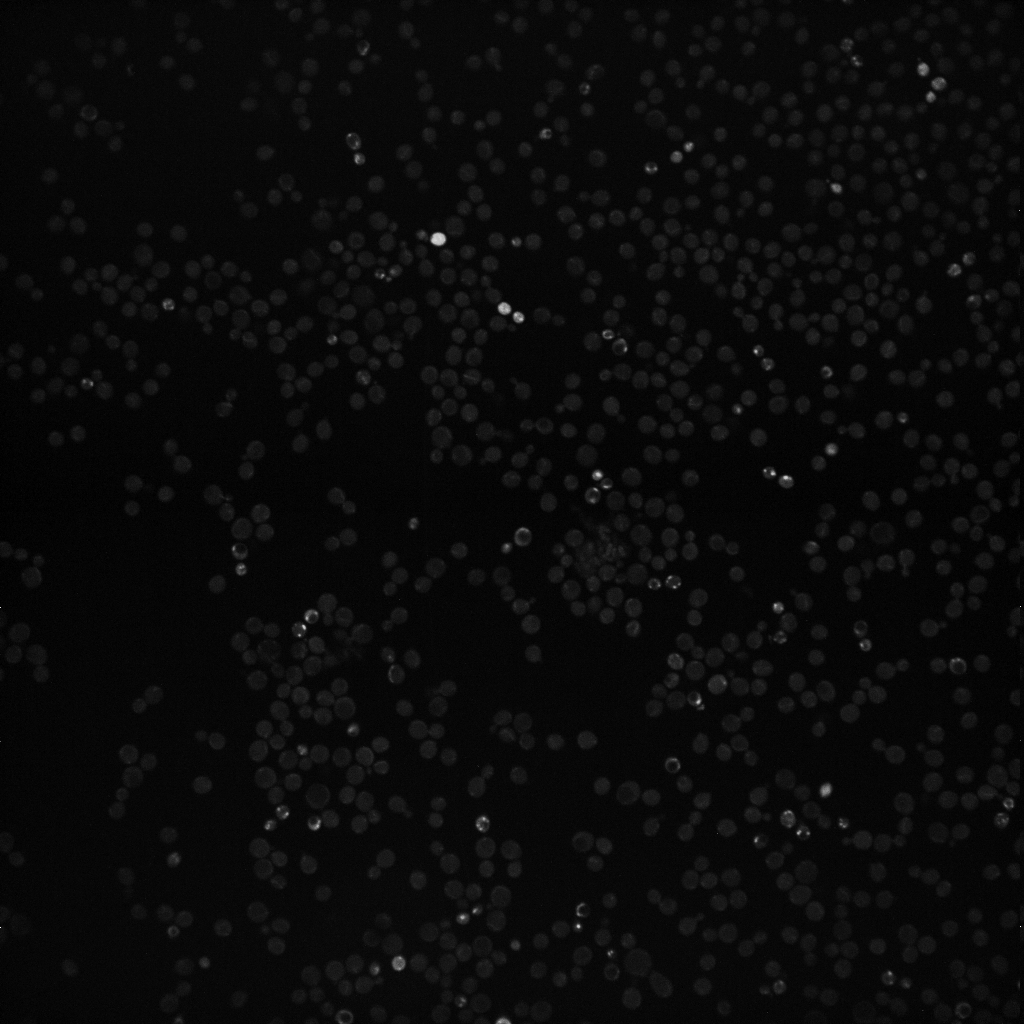

Supplement: Figure 4—source data 4. [file elife-98889-fig4-data4.zip › SourceData-Fig4F/GPP1-TEFpr-3HA/GPP1_NaCl_log_repeat1_w2confGFP_s12.tif]

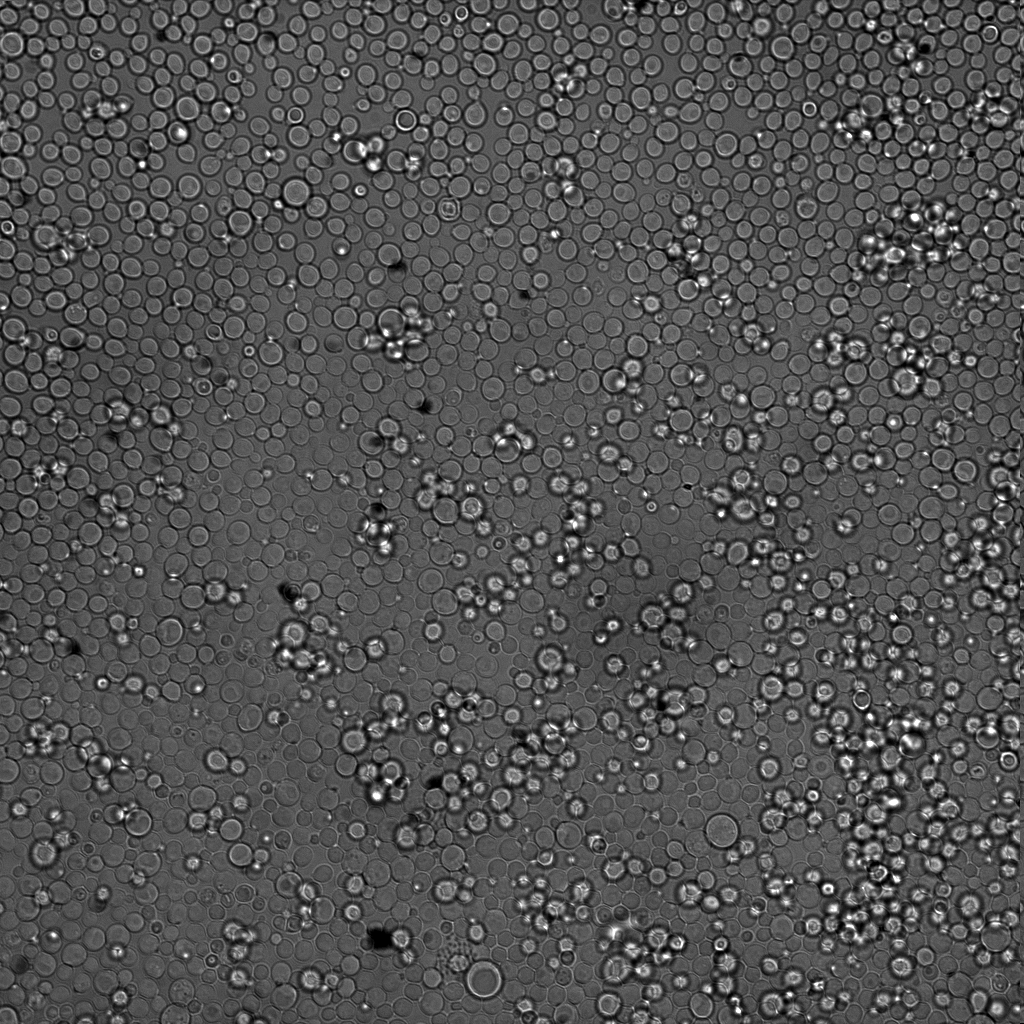

Supplement: Figure 4—source data 4. [file elife-98889-fig4-data4.zip › SourceData-Fig4F/GPP2-TEFpr/GPP1_NaCl_log_repeat1_w1Brightfield_s28.tif]

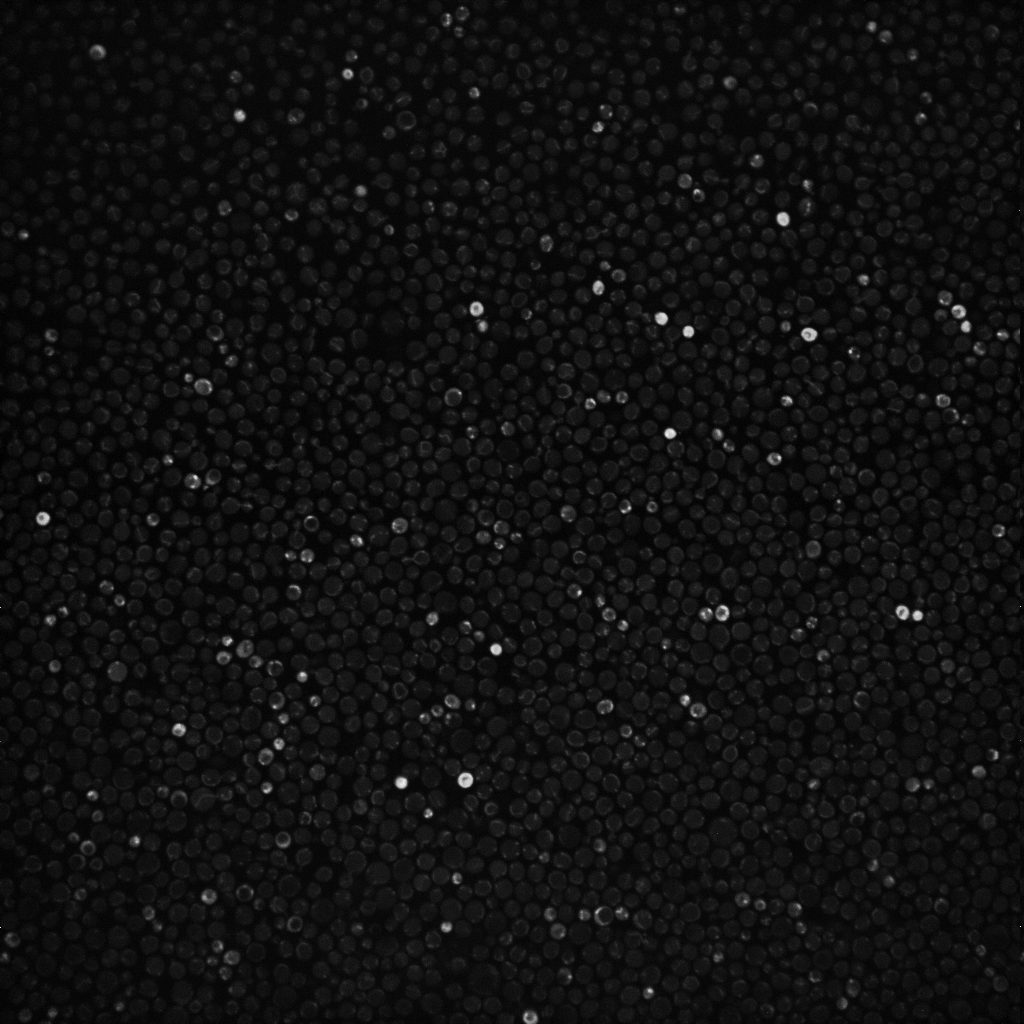

Supplement: Figure 4—source data 4. [file elife-98889-fig4-data4.zip › SourceData-Fig4F/GPP2-TEFpr/GPP1_NaCl_log_repeat1_w2confGFP_s28.tif]

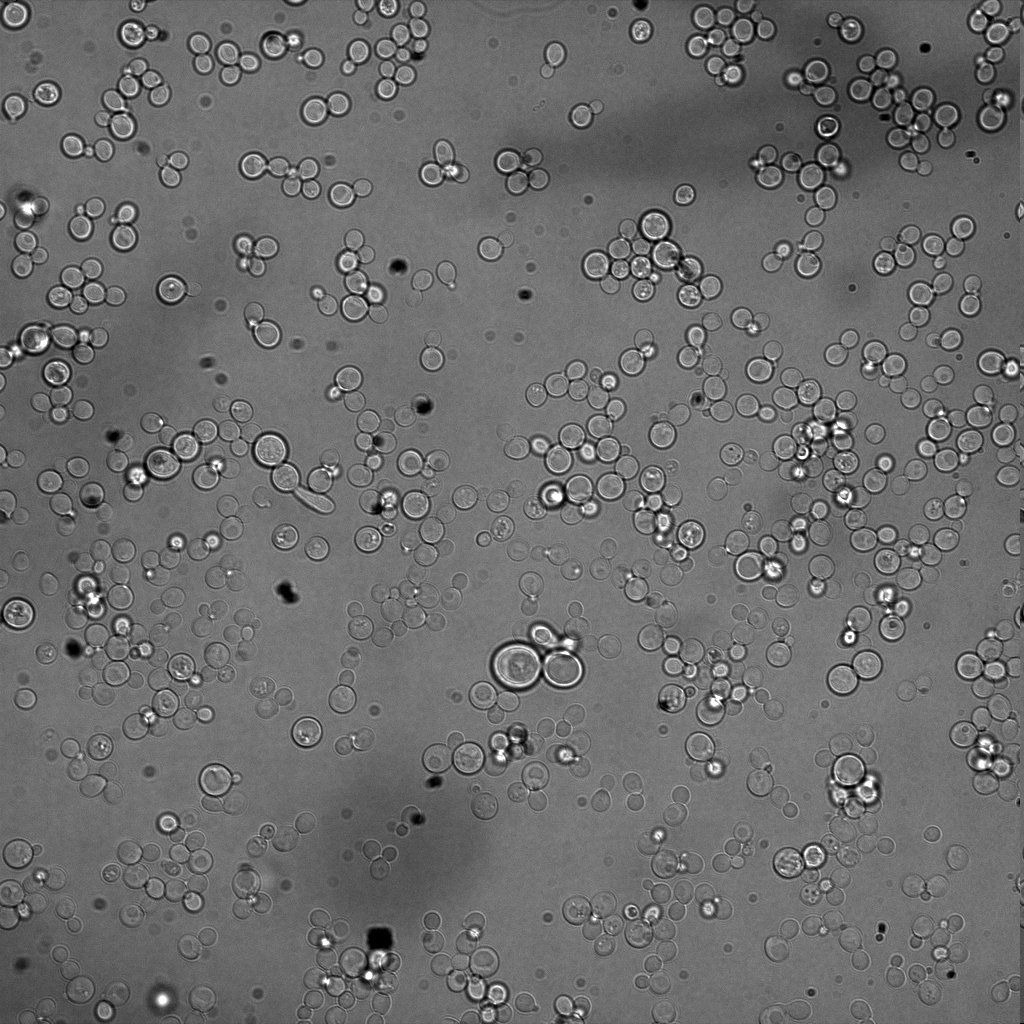

Supplement: Figure 4—source data 4. [file elife-98889-fig4-data4.zip › SourceData-Fig4F/GPP2-TEFpr-MTS-3HA/GPP1_NaCl_log_repeat1_w1Brightfield_s48.tif]

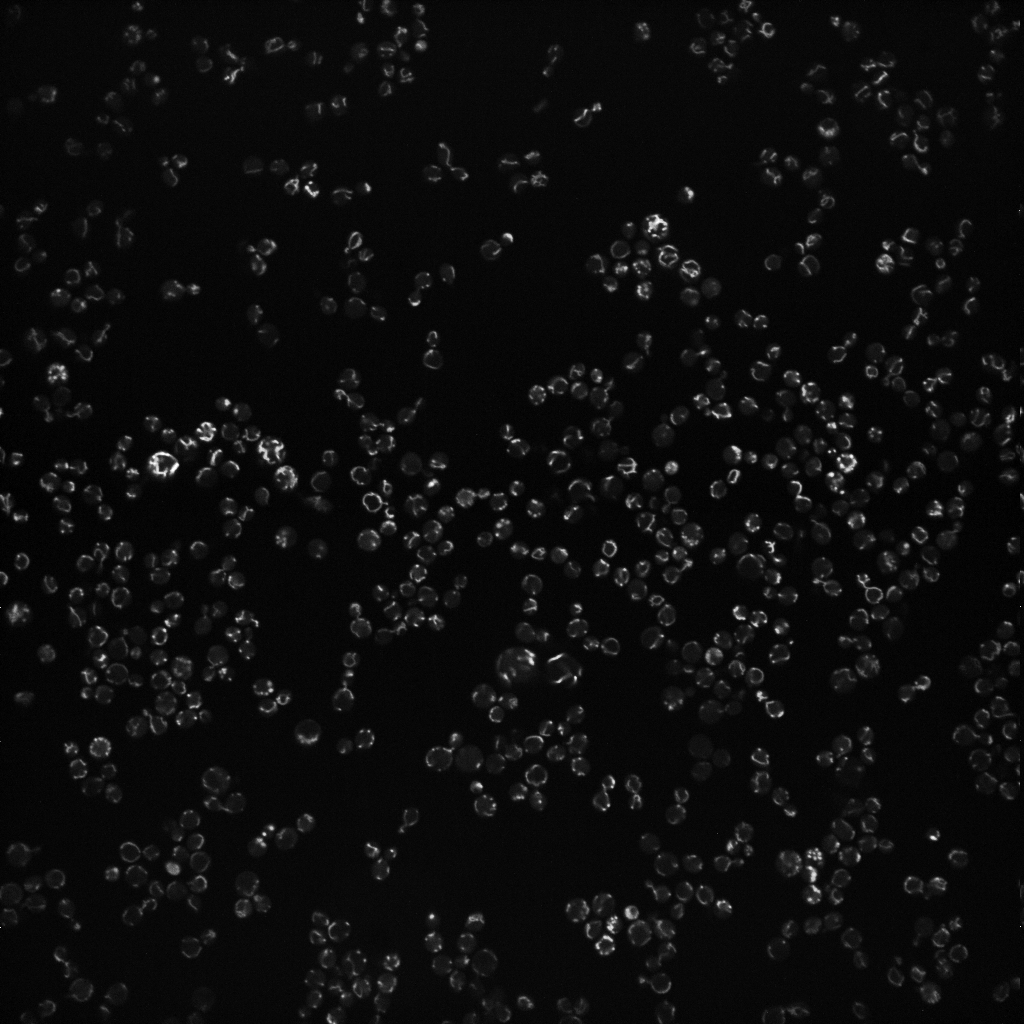

Supplement: Figure 4—source data 4. [file elife-98889-fig4-data4.zip › SourceData-Fig4F/GPP2-TEFpr-MTS-3HA/GPP1_NaCl_log_repeat1_w2confGFP_s48.tif]

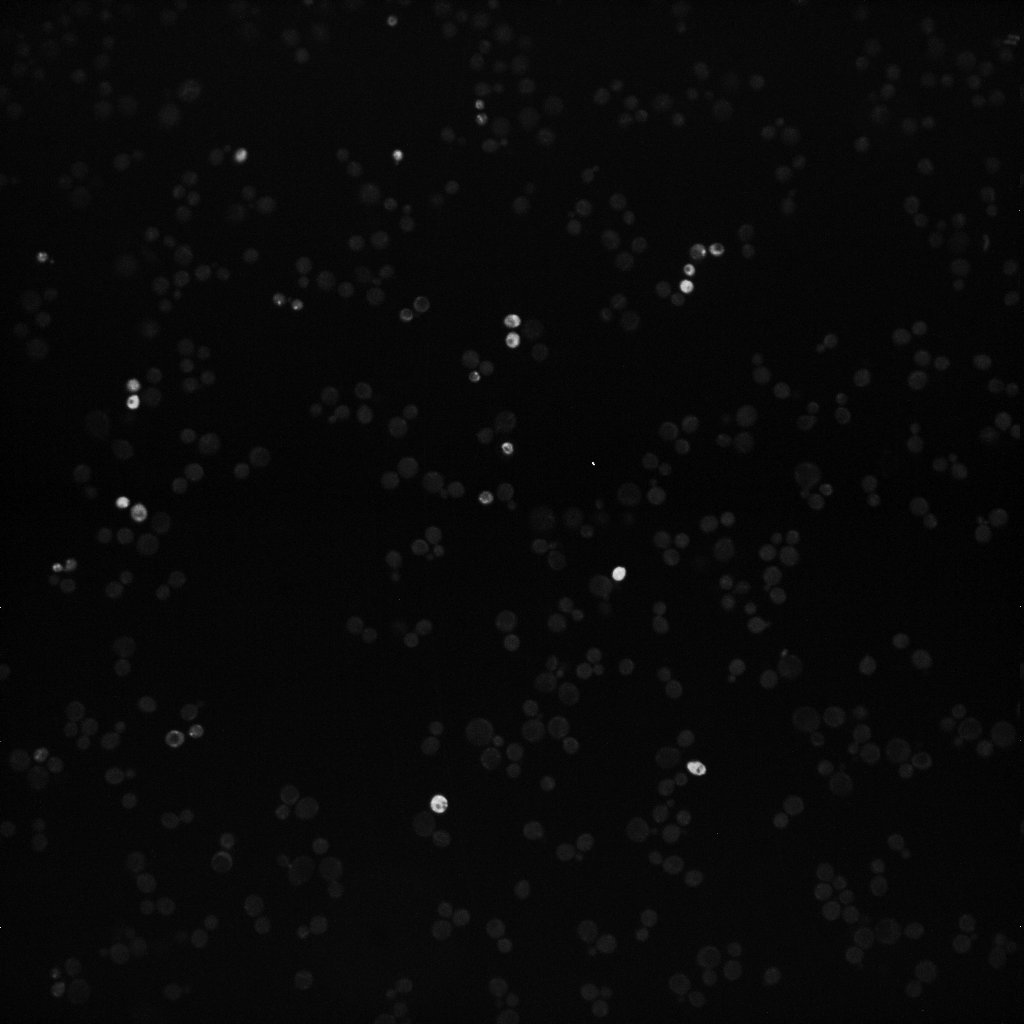

Supplement: Figure 4—source data 4. [file elife-98889-fig4-data4.zip › SourceData-Fig4F/GPP2-NATIVEpr/GPP1_NaCl_log_repeat1_w2confGFP_s36.tif]

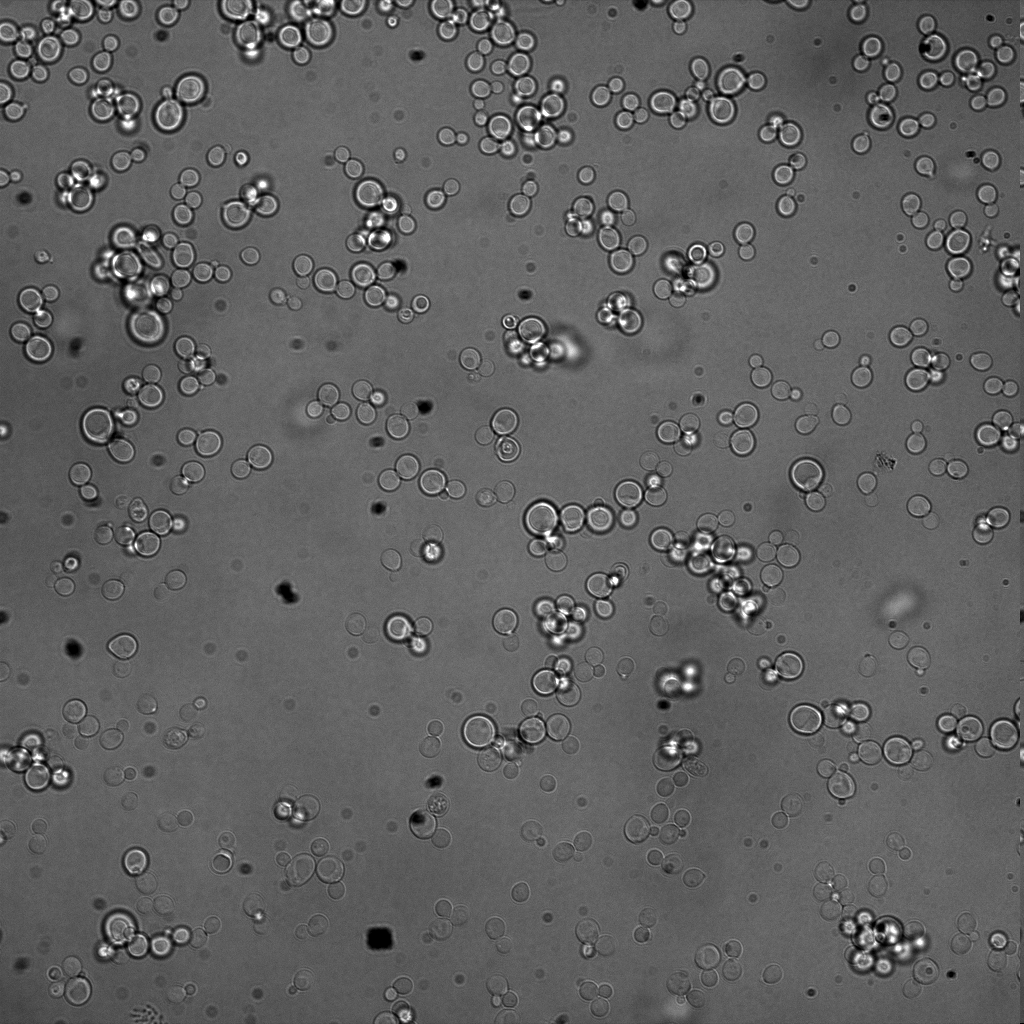

Supplement: Figure 4—source data 4. [file elife-98889-fig4-data4.zip › SourceData-Fig4F/GPP2-NATIVEpr/GPP1_NaCl_log_repeat1_w1Brightfield_s36.tif]

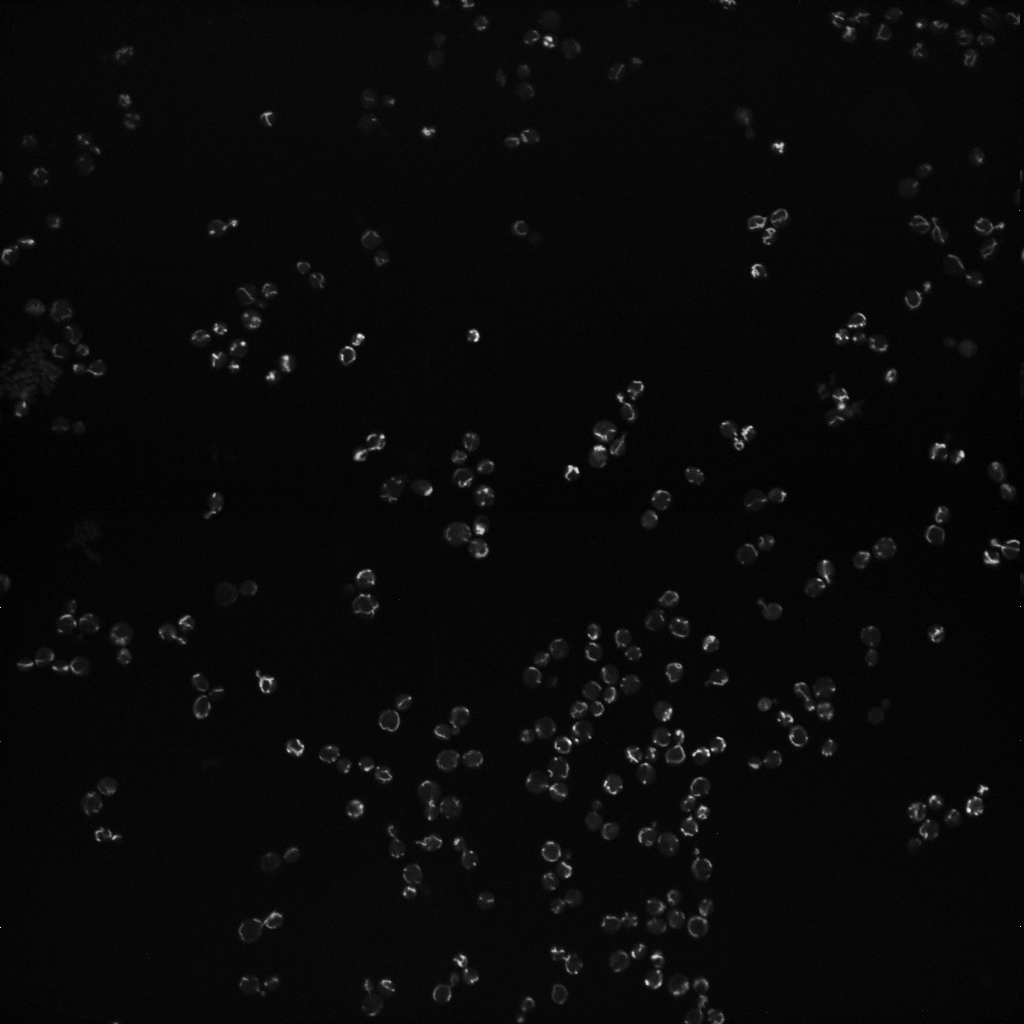

Supplement: Figure 4—source data 4. [file elife-98889-fig4-data4.zip › SourceData-Fig4F/GPP1-TEFpr-MTS-3HA/GPP1_NaCl_log_repeat1_w2confGFP_s20.tif]

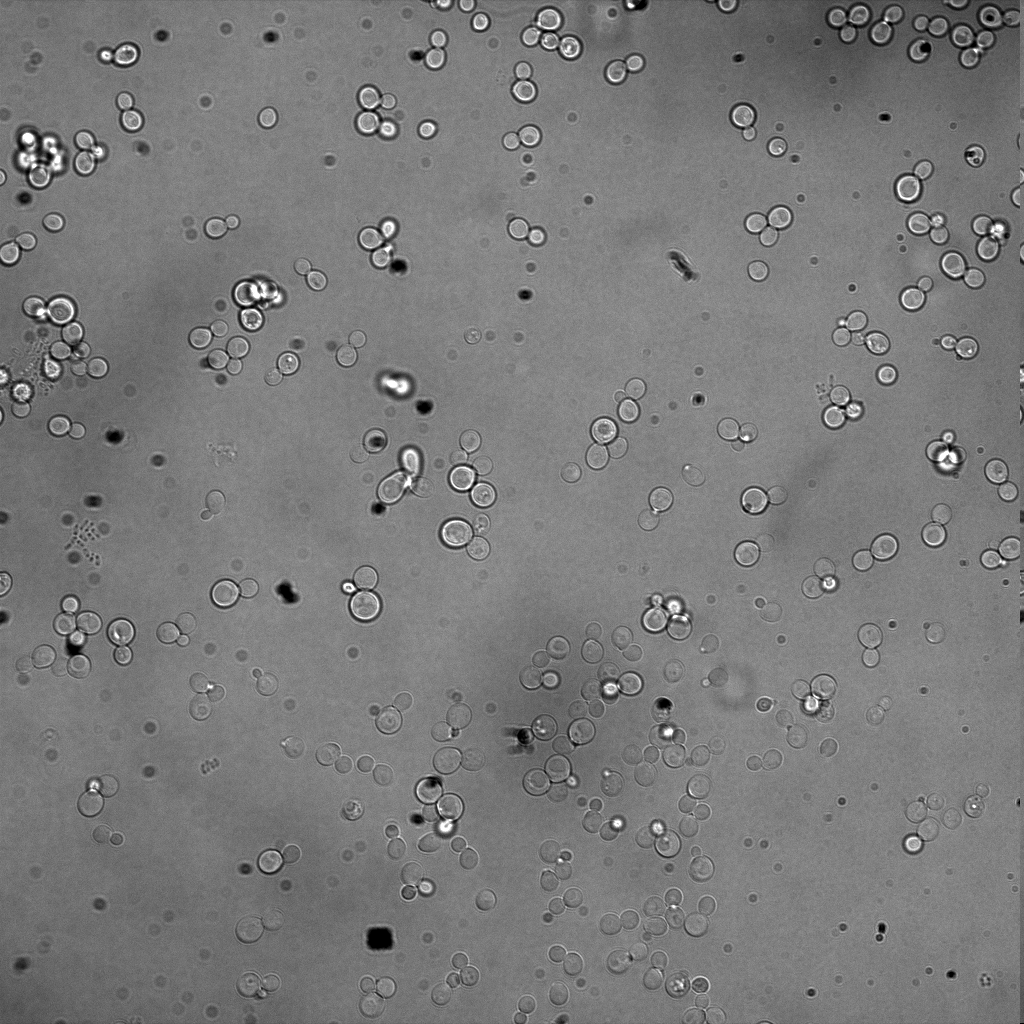

Supplement: Figure 4—source data 4. [file elife-98889-fig4-data4.zip › SourceData-Fig4F/GPP1-TEFpr-MTS-3HA/GPP1_NaCl_log_repeat1_w1Brightfield_s20.tif]

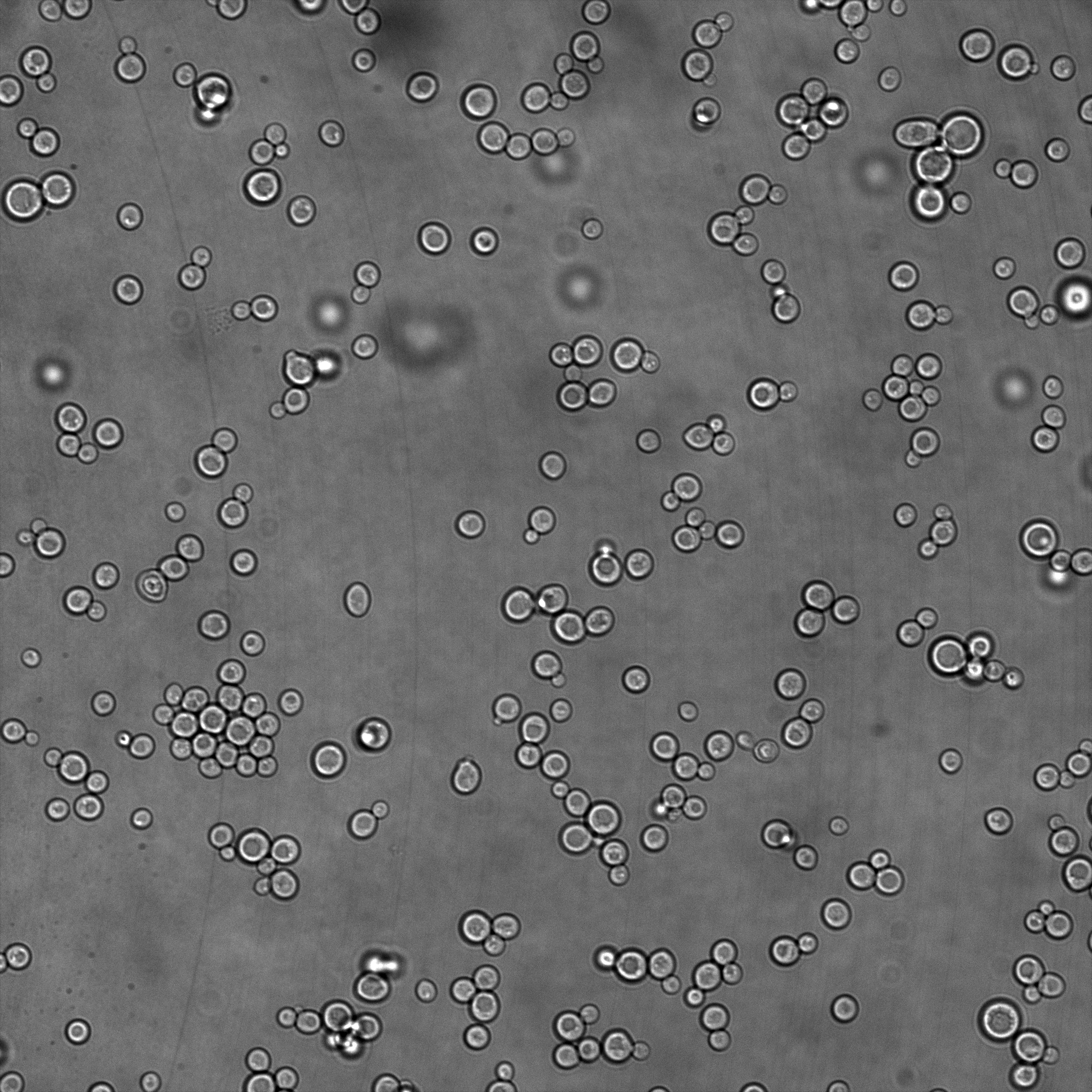

Supplement: Figure 4—source data 5. [file elife-98889-fig4-data5.zip › SourceData-Fig4G/NATIVEpr-GPP1/B13--W00037--P00002--Z00000--T00000--TransCon.tif]

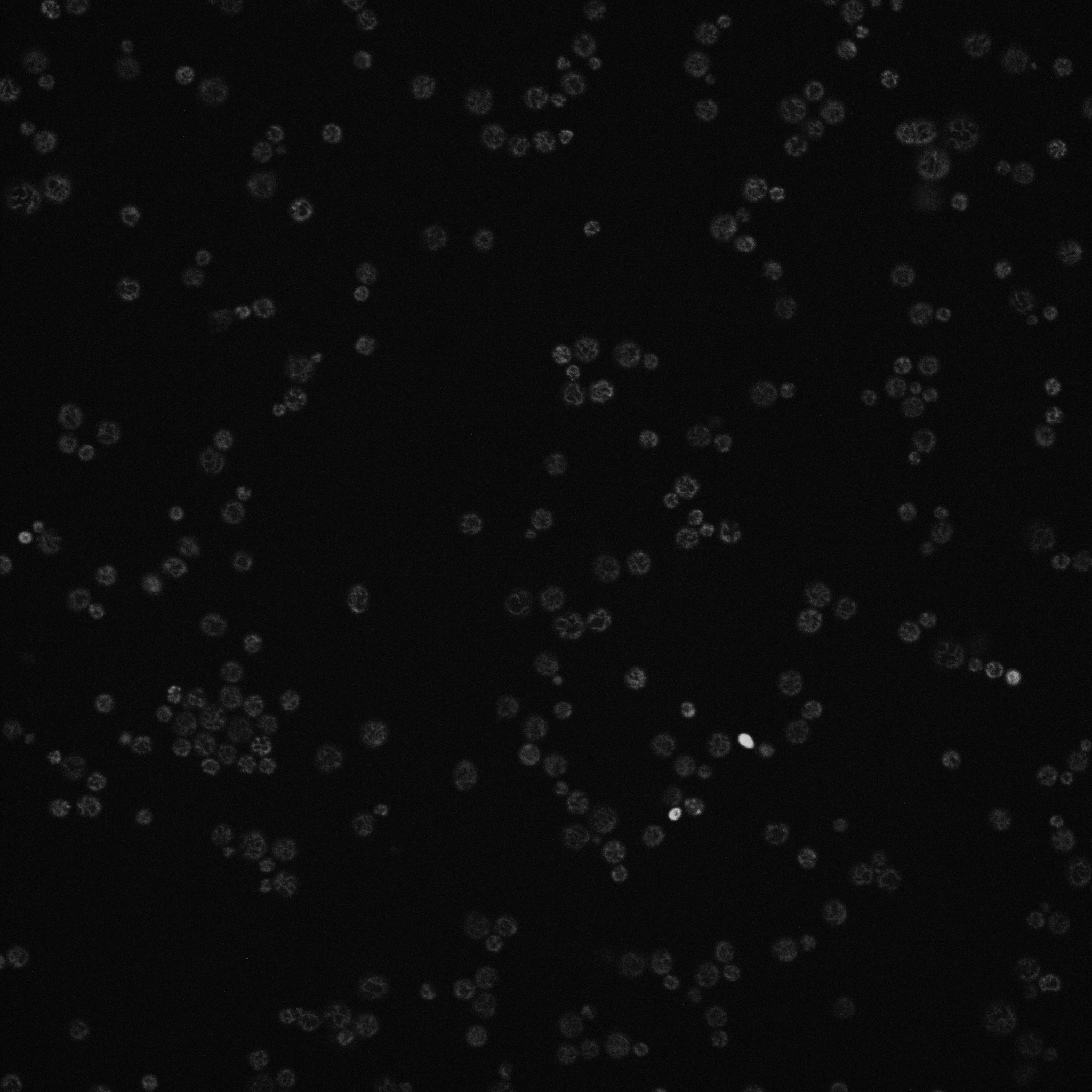

Supplement: Figure 4—source data 5. [file elife-98889-fig4-data5.zip › SourceData-Fig4G/NATIVEpr-GPP1/B13--W00037--P00002--Z00000--T00000--488nm.tif]

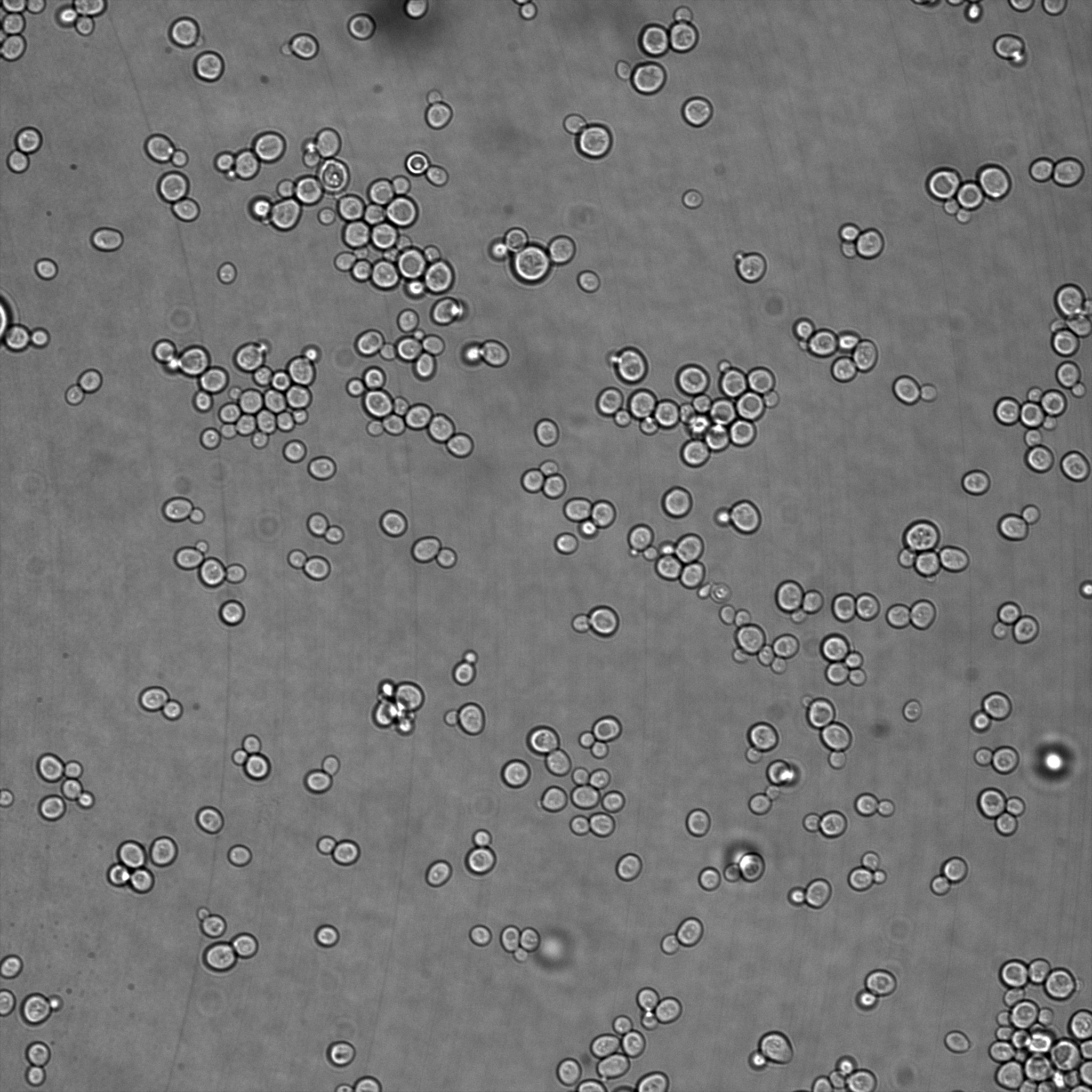

Supplement: Figure 4—source data 5. [file elife-98889-fig4-data5.zip › SourceData-Fig4G/No_GFP_YMS3553/B14--W00038--P00002--Z00000--T00000--TransCon.tif]

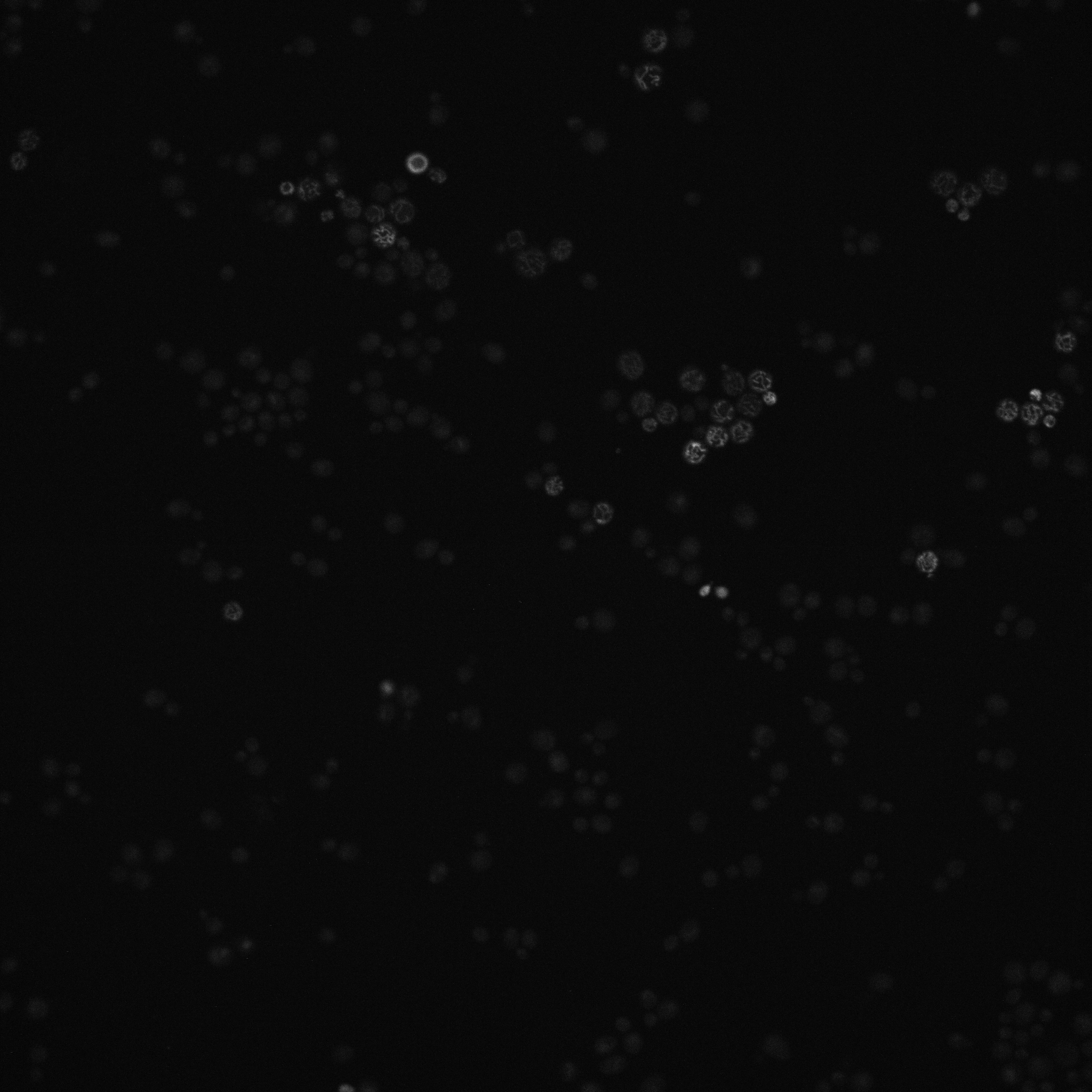

Supplement: Figure 4—source data 5. [file elife-98889-fig4-data5.zip › SourceData-Fig4G/No_GFP_YMS3553/B14--W00038--P00002--Z00000--T00000--488nm.tif]

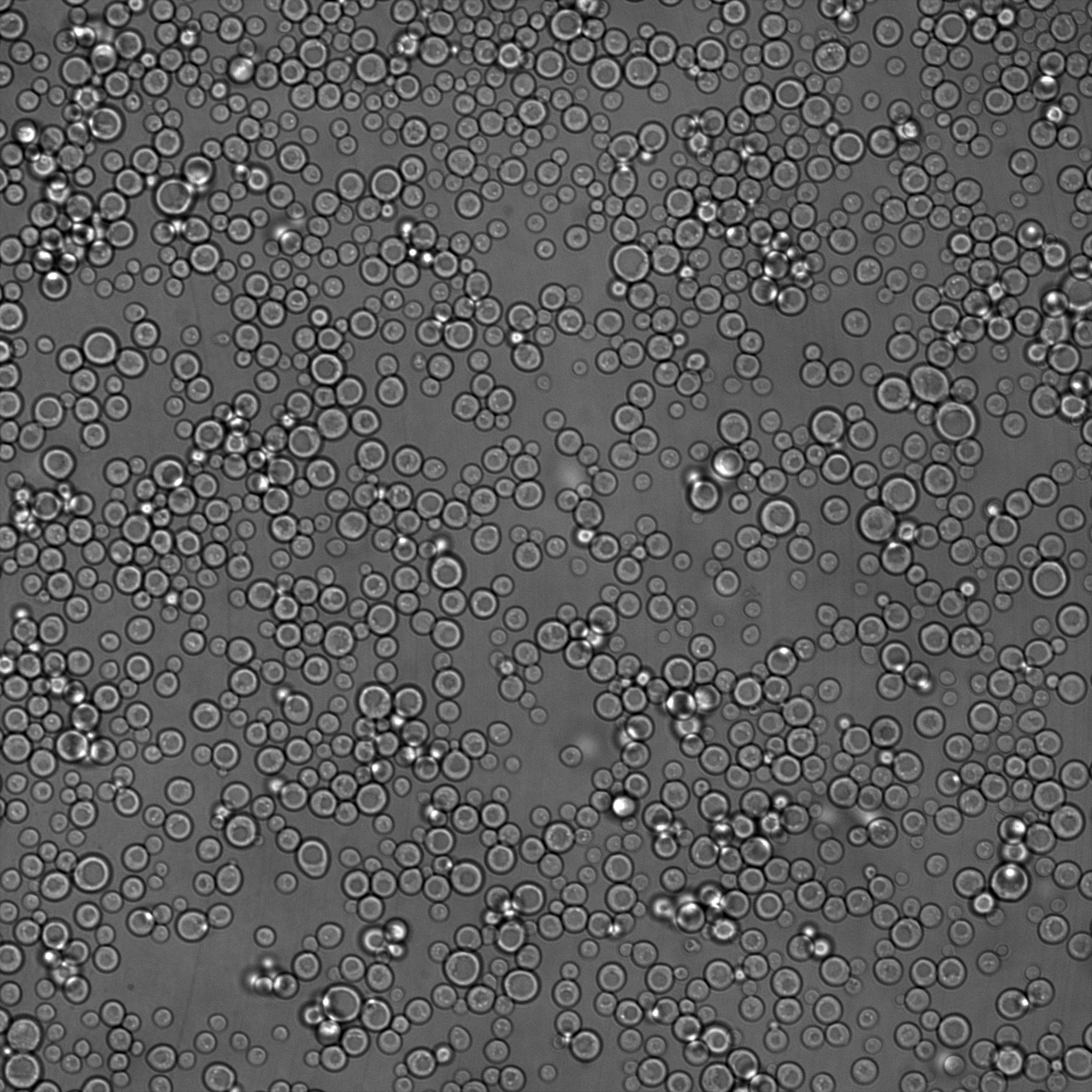

Supplement: Figure 4—source data 5. [file elife-98889-fig4-data5.zip › SourceData-Fig4G/TEF(-39)-GPP1_TF281/B9--W00033--P00001--Z00000--T00000--TransCon.tif]

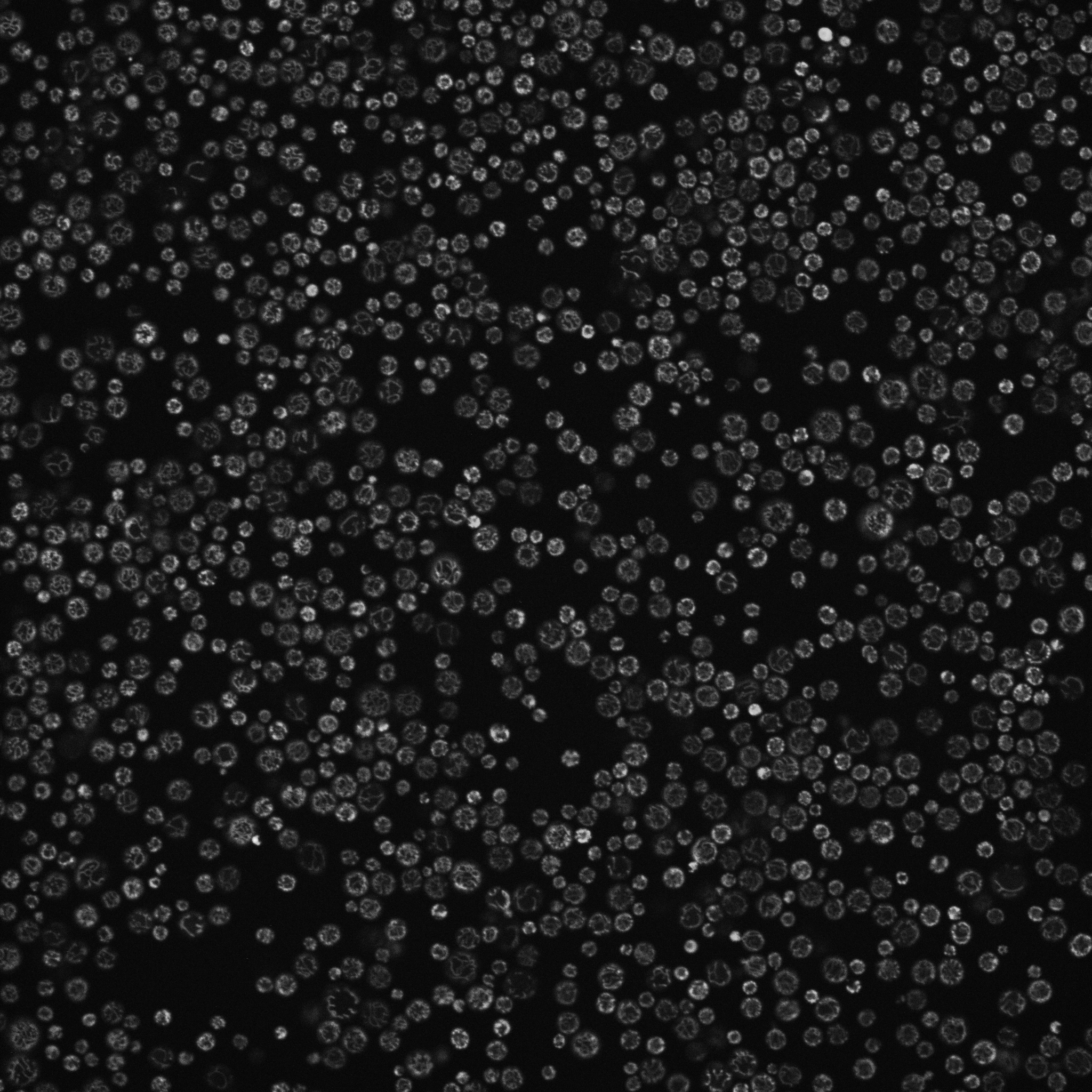

Supplement: Figure 4—source data 5. [file elife-98889-fig4-data5.zip › SourceData-Fig4G/TEF(-39)-GPP1_TF281/B9--W00033--P00001--Z00000--T00000--488nm.tif]

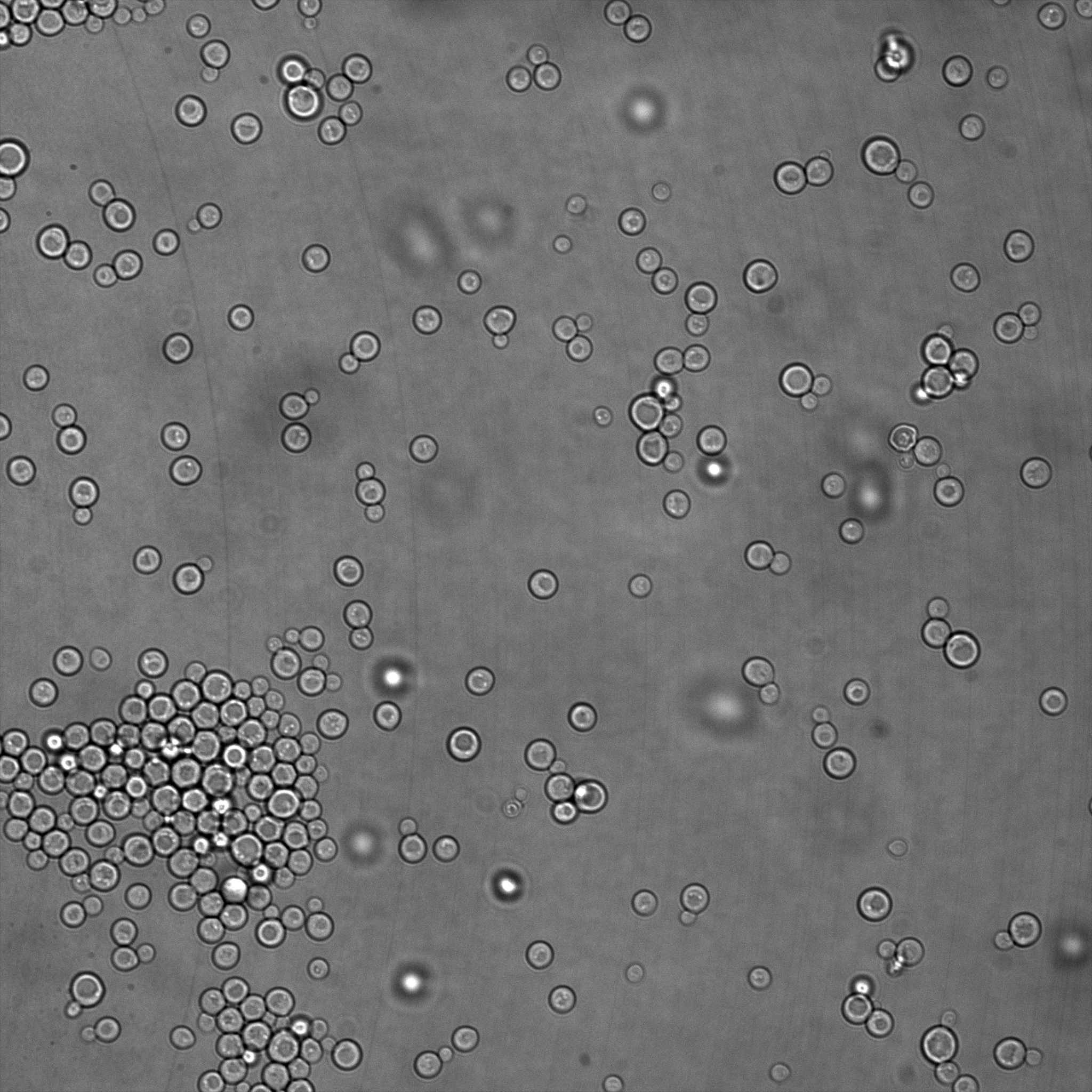

Supplement: Figure 4—source data 5. [file elife-98889-fig4-data5.zip › SourceData-Fig4G/TEF(-21)-GPP1_TF282/B2--W00026--P00001--Z00000--T00000--TransCon.tif]

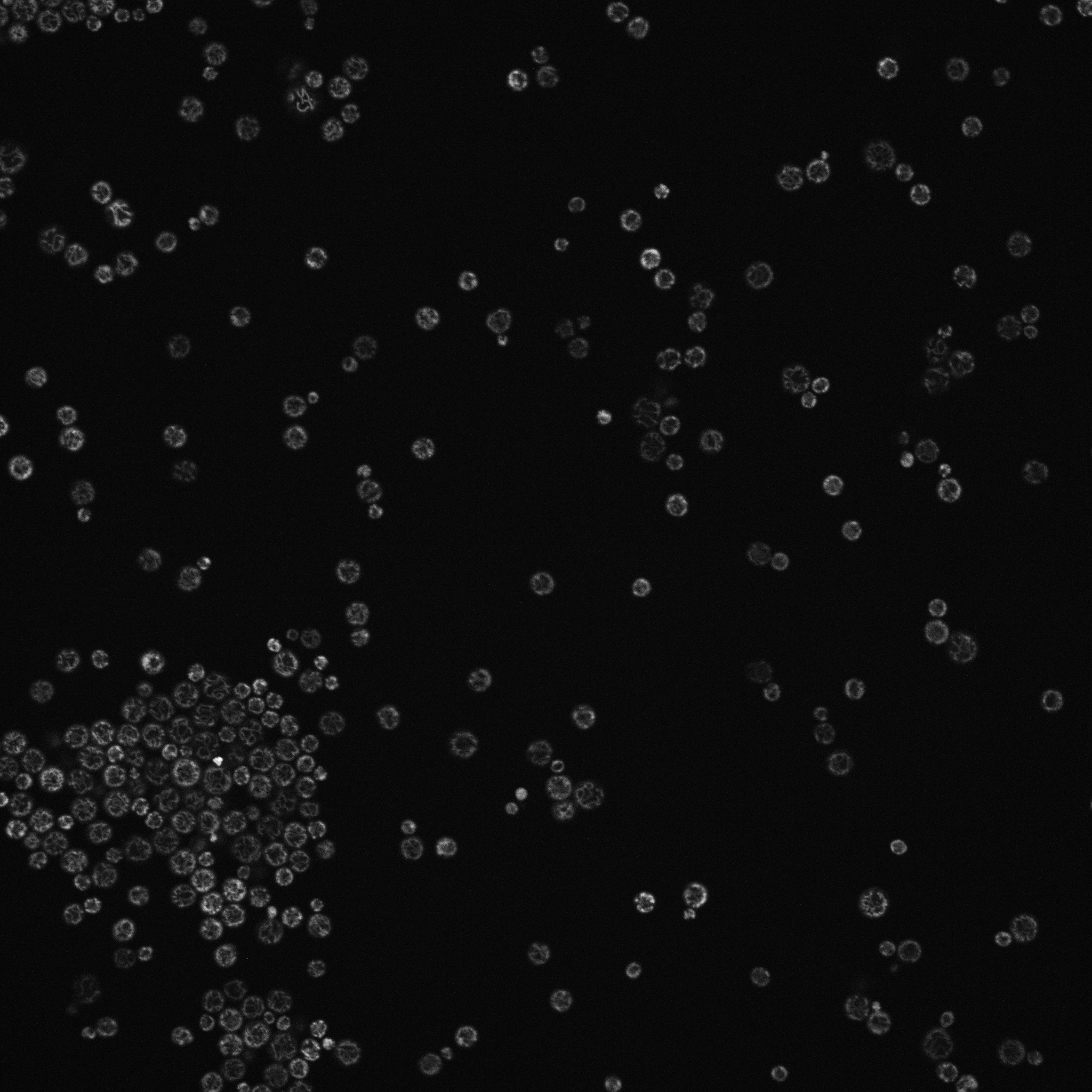

Supplement: Figure 4—source data 5. [file elife-98889-fig4-data5.zip › SourceData-Fig4G/TEF(-21)-GPP1_TF282/B2--W00026--P00001--Z00000--T00000--488nm.tif]
