# Supplementary material for: A systematic bi-genomic split-GFP assay illuminates the mitochondrial matrix proteome and protein targeting routes
Source: eLife. 2025 Dec 16;13:RP98889. doi: 10.7554/eLife.98889 (PMC12707816; doi:10.7554/eLife.98889)
Supplement: Figure 3—figure supplement 2—source data 1. [file elife-98889-fig3-figsupp2-data1.zip › Fig3supp2-sourcedata1/Figure 3, figure supplement 2_Source data 1.pdf]

IB : GFP_{11}

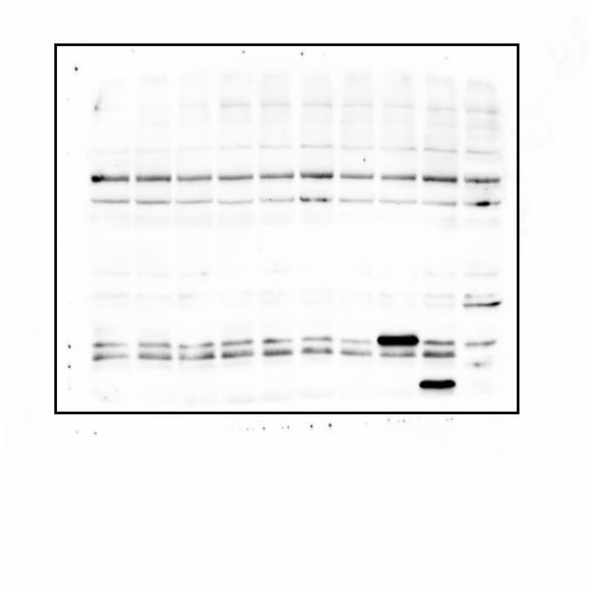

IB : ADH1

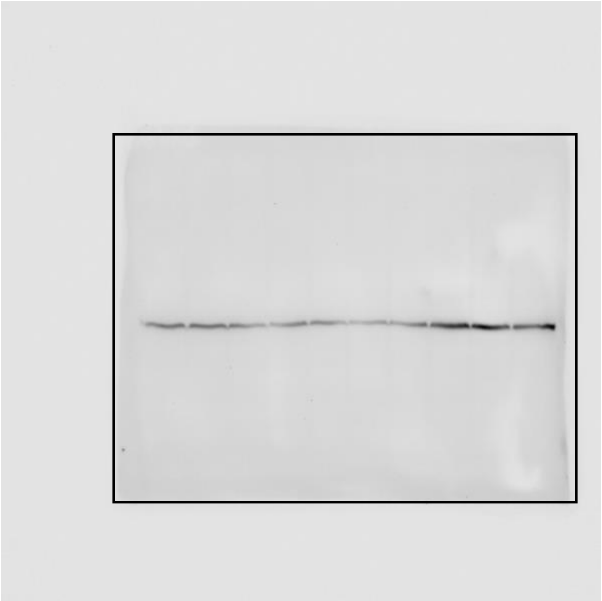

Merge: GFP_{B11}

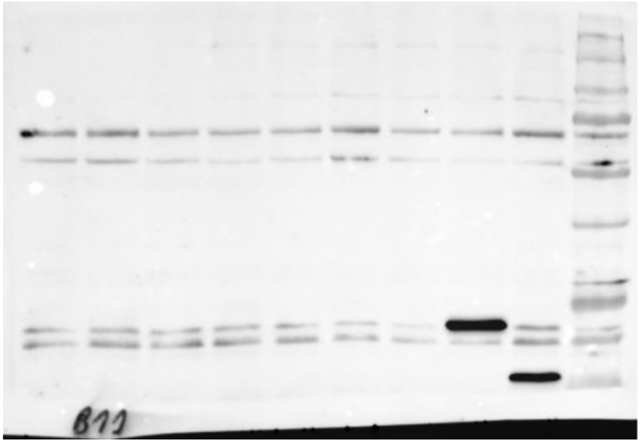

Merge : ADH1

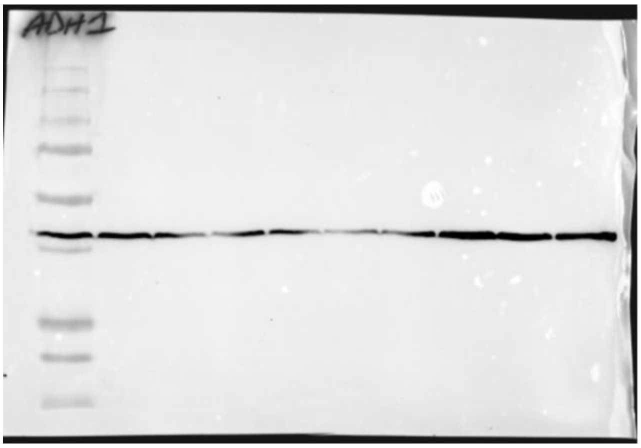

Figure 3, figure supplement 2, Source Data 1. Original membranes (ECL signal) and merge (ladder + ECL signal) corresponding to Figure 3, figure supplement 2, panel B. Precision Plus Protein™ All Blue from Biorad was used as molecular weight marker.
