# Supplementary material for: A systematic bi-genomic split-GFP assay illuminates the mitochondrial matrix proteome and protein targeting routes
Source: eLife. 2025 Dec 16;13:RP98889. doi: 10.7554/eLife.98889 (PMC12707816; doi:10.7554/eLife.98889)
Supplement: Figure 4—source data 1. [file elife-98889-fig4-data1.zip › Fig4sourcedata1/Figure 4_Source data 1.pdf]

IB : GFP_{B1-10}

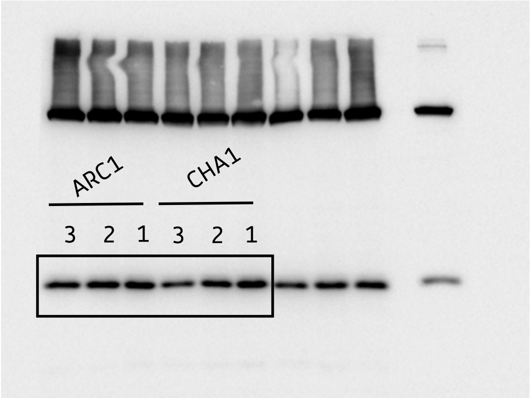

IB : GFP_{B11}

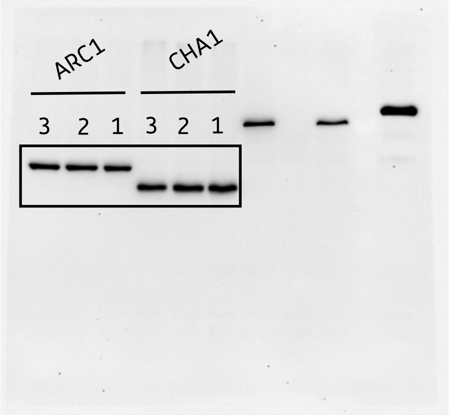

IB : ADH1

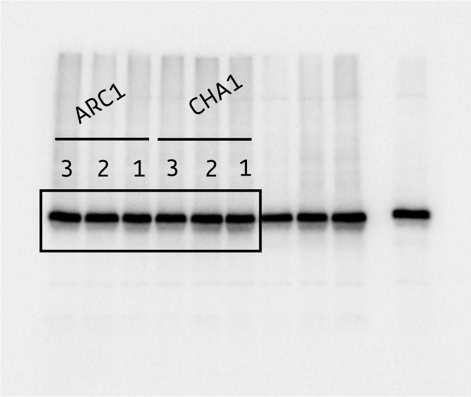

Merge : GFP_{B1-10}

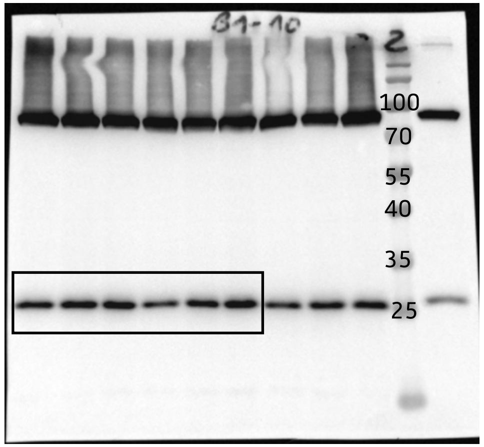

Merge : GFP_{B11}

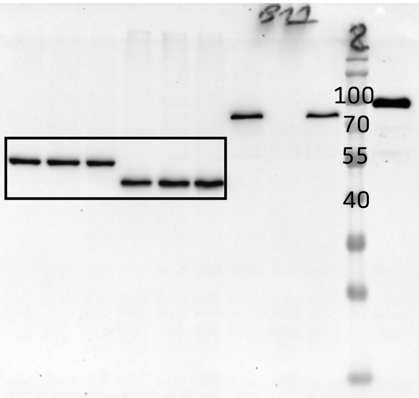

Merge : ADH1

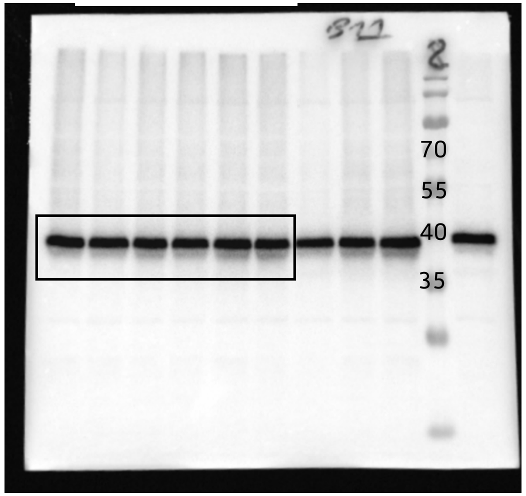

Figure 4, Source Data 1. Original membranes (ECL signal) and merge (ladder + ECL signal) corresponding to Figure 4, panel C. PageRuler™ Prestained Protein Ladder from Thermo Scientific™ was used as molecular weight marker.
