# Supplementary material for: A systematic bi-genomic split-GFP assay illuminates the mitochondrial matrix proteome and protein targeting routes
Source: eLife. 2025 Dec 16;13:RP98889. doi: 10.7554/eLife.98889 (PMC12707816; doi:10.7554/eLife.98889)
Supplement: MDAR checklist [file elife-98889-mdarchecklist1.docx]

Materials Design Analysis Reporting (MDAR)

Checklist for Authors

The MDAR framework establishes a minimum set of requirements in transparent reporting mainly

applicable to studies in the life sciences.

eLife asks authors to provide detailed information within their article to facilitate the interpretation

and replication of their work. Authors can also upload supporting materials to comply with relevant

reporting guidelines for health-related research (see EQUATOR Network), life science research (see

the BioSharing Information Resource), or animal research (see the ARRIVE Guidelines and the

STRANGE Framework; for details, see eLife’s Journal Policies). Where applicable, authors should refer

to any relevant reporting standards materials in this form.

For all that apply, please note where in the article the information is provided. Please note that we

also collect information about data availability and ethics in the submission form.

| **Newly created materials** | **Indicate where provided:**  **section/figure legend** | **N/A** |
| --- | --- | --- |
| The manuscript includes a dedicated "materials availability  statement" providing transparent disclosure about availability  of newly created materials including details on how materials  can be accessed and describing any restrictions on access. | At the end of the methods section there is “Materials and data availability statement”:  “All the created plasmids and yeast strains including the systematic collections are available from the corresponding authors upon request.” |  |

| **Antibodies** | **Indicate where provided:**  **section/figure legend** | **N/A** |
| --- | --- | --- |
| For commercial reagents, provide supplier name, catalogue  number and RRID, if available. | Provided in the Key resources table |  |

| **DNA and RNA sequences** | **Indicate where provided:**  **section/figure legend** | **N/A** |
| --- | --- | --- |
| Short novel DNA or RNA including primers, probes: Sequences  should be included or deposited in a public repository. | Primer sequences are listed in the Supplementary file Table_S6 |  |

| **Cell lines** | **Indicate where provided:**  **section/figure legend** | **N/A** |
| --- | --- | --- |
| Cell lines: Provide species information, strain. Provide  accession number in repository OR supplier name, catalog  number, clone number, OR RRID. | All the yeast strains are listed in the Supplementary file Table_S4. The availability is mentioned in the “Materials availability statement” |  |

| **Experimental animals** | **Indicate where provided:**  **section/figure legend** | **N/A** |
| --- | --- | --- |
| Does not apply | Does not apply |  |

| **Plants and microbes** | **Indicate where provided:**  **section/figure legend** | **N/A** |
| --- | --- | --- |
| Does not apply | Does not apply |  |

| **Human research participants** | **Indicate where provided:**  **section/figure legend** | **N/A** |
| --- | --- | --- |
| Does not apply | Does not apply |  |

**Design:**

| **Study protocol** | **Indicate where provided:**  **section/figure legend** | **N/A** |
| --- | --- | --- |
| Does not applyIf the study protocol has been pre-registered, provide DOI. For  clinical trials, provide the trial registration number OR cite DOI. | The study protocol was not pre-registered |  |

| **Laboratory protocol** | **Indicate where provided:**  **section/figure legend** | **N/A** |
| --- | --- | --- |
| Provide DOI OR other citation details if detailed step-by-step  protocols are available. | Protocols are available upon request |  |

| **Experimental study design (statistics details)*** | | |
| --- | --- | --- |
| **For in vivo studies: State whether and how the following**  **have been done** | **Indicate where provided:**  **section/figure legend. If it could**  **have been done, but was not,**  **write “not done”** | **N/A** |
| Sample size determination | Not done |  |
| Randomisation | Does not apply |  |
| Blinding | Not done |  |
| Inclusion/exclusion criteria | Yes – see the Methods section “Fluorescence quantitation” |  |

| **Sample definition and in-laboratory replication** | **Indicate where provided:**  **section/figure legend** | **N/A** |
| --- | --- | --- |
| State number of times the experiment was replicated in the  laboratory. | Screens were not replicated. Individual experiments were replicated at least three times. Sample sizes are indicated in the figures. |  |

| **Ethics** | **Indicate where provided:**  **section/figure legend** | **N/A** |
| --- | --- | --- |
| Studies involving human participants: State details of authority  granting ethics approval (IRB or equivalent committee(s),  provide reference number for approval. | Does not apply |  |
| Studies involving experimental animals: State details of  authority granting ethics approval (IRB or equivalent  committee(s), provide reference number for approval. | Does not apply |  |
| Studies involving specimen and field samples: State if relevant  permits obtained, provide details of authority approving study;  if none were required, explain why. | Does not apply |  |

| **Dual Use Research of Concern (DURC)** | **Indicate where provided:**  **section/figure legend** | **N/A** |
| --- | --- | --- |
| If study is subject to dual use research of concern regulations,  state the authority granting approval and reference number for  the regulatory approval. | Does not apply |  |

**Analysis:**

| **Attrition** | **Indicate where provided:**  **section/figure legend** | **N/A** |
| --- | --- | --- |
| Describe whether exclusion criteria were pre-established.  Report if sample or data points were omitted from analysis. If  yes, report if this was due to attrition or intentional exclusion  and provide justification. | Methods section “Fluorescence quantitation” |  |

| **Statistics** | **Indicate where provided:**  **section/figure legend** | **N/A** |
| --- | --- | --- |
| Describe statistical tests used and justify choice of tests | Indicated in the Methods section “Fluorescence quantitation” and in the figure legends when applicable. |  |

| **Data availability** | **Indicate where provided:**  **section/figure legend** | **N/A** |
| --- | --- | --- |
| For newly created and reused datasets, the manuscript  includes a data availability statement that provides details for access (or notes restrictions on access). | The image dataset for the quantification of fluorescence is deposited in the BioImage Archive. The statement is indicated in the submission form and in the “Materials and data availability statement” |  |
| When newly created datasets are publicly available, provide  accession number in repository OR DOI and licensing details  where available. | Accession numbers are provided in the manuscript. BioImage Archive S-BIAD2409. |  |
| If reused data is publicly available provide accession number in  repository OR DOI, OR URL, OR citation. | All the reused datasets are cited where appropriate in the Methods section “Additional data analysis” and in the figure legends. |  |

| **Code availability** | **Indicate where provided:**  **section/figure legend** | **N/A** |
| --- | --- | --- |
| For any computer code/software/mathematical algorithms  essential for replicating the main findings of the study,  whether newly generated or re-used, the manuscript includes  a data availability statement that provides details for access or  notes restrictions. | Does not apply |  |
| Where newly generated code is publicly available, provide  accession number in repository, OR DOI OR URL and licensing  details where available. State any restrictions on code  availability or accessibility. If reused code is publicly available provide accession number in  repository OR DOI OR URL, OR citation. | Does not apply |  |
| If reused data is publicly available provide accession number in  repository OR DOI, OR URL, OR citation. | All the reused datasets are cited where appropriate in the Methods section “Additional data analysis” and in the figure legends. |  |

**Reporting:**

| **Adherence to community standards** | **Indicate where provided:**  **section/figure legend** | **N/A** |
| --- | --- | --- |
| State if relevant guidelines (e.g., ICMJE, MIBBI, ARRIVE,  STRANGE) have been followed, and whether a checklist (e.g.,  CONSORT, PRISMA, ARRIVE) is provided with the manuscript. | Does not apply |  |

* We provide the following guidance regarding transparent reporting and statistics; we also refer authors to

Ten common statistical mistakes to watch out for when writing or reviewing a manuscript.

Sample-size estimation

● You should state whether an appropriate sample size was computed when the study was being

designed

● You should state the statistical method of sample size computation and any required assumptions

● If no explicit power analysis was used, you should describe how you decided what sample

(replicate) size (number) to use

Replicates

● You should report how often each experiment was performed

● You should include a definition of biological versus technical replication

● The data obtained should be provided and sufficient information should be provided to indicate the

number of independent biological and/or technical replicates

● If you encountered any outliers, you should describe how these were handled

● Criteria for exclusion/inclusion of data should be clearly stated

● High-throughput sequence data should be uploaded before submission, with a private link for

reviewers provided (these are available from both GEO and ArrayExpress)

Statistical reporting

● Statistical analysis methods should be described and justified

● Raw data should be presented in figures whenever informative to do so (typically when N per group

is less than 10)

● For each experiment, you should identify the statistical tests used, exact values of N, definitions of

center, methods of multiple test correction, and dispersion and precision measures (e.g., mean,

median, SD, SEM, confidence intervals; and, for the major substantive results, a measure of effect

size (e.g., Pearson's r, Cohen's d)

● Report exact p-values wherever possible alongside the summary statistics and 95% confidence

intervals. These should be reported for all key questions and not only when the p-value is less than

0.05.

Group allocation

● Indicate how samples were allocated into experimental groups (in the case of clinical studies,

6please specify allocation to treatment method); if randomization was used, please also state if

restricted randomization was applied

● Indicate if masking was used during group allocation, data collection and/or data analysis
